# Metabolic stability and metabolite profiling of emerging synthetic cathinones

**DOI:** 10.3389/fphar.2023.1145140

**Published:** 2023-03-24

**Authors:** Rita P. Lopes, Raquel A. Ferro, Margarida Milhazes, Margarida Figueira, Maria João Caldeira, Alexandra M. M. Antunes, Helena Gaspar

**Affiliations:** ^1^ Centro de Química Estrutural (CQE), Institute of Molecular Sciences, Departamento de Engenharia Química, Instituto Superior Técnico (IST), Universidade de Lisboa, Lisboa, Portugal; ^2^ BioISI—Biosystems & Integrative Sciences Institute, Faculty of Sciences, University of Lisbon, Lisboa, Portugal; ^3^ Laboratório de Polícia Científica da Polícia Judiciária (LPC/PJ), Novo edifício Sede da Polícia Judiciária, Lisboa, Portugal; ^4^ MARE—Marine and Environmental Sciences Centre—Polytechnic of Leiria, Peniche, Portugal

**Keywords:** 4-CMC, 3-CMC, 4-CIC, 4-MEAP, 3-CIC, 4-MDMB, 4-MNEB and 4-MDMP

## Abstract

Synthetic cathinones constitute the second largest groups of new psychoactive substances (NPS), which are especially popular among adolescents and young adults. Due to their potential toxicity, the recreational use of these NPS constitute a serious worldwide public health problem. However, their fast appearance in the market renders the continuous updating of NPS information highly challenging for forensic authorities. The unavailability of pharmacokinetic data for emerging NPS is critical for forensic and clinical verifications. With the ultimate goal of having a proactive approach towards the NPS issue, high resolution mass spectrometry was used in the current work to assess preliminary pharmacokinetic data for 8 selected cathinones: 4 reported substances (**4-CIC**, **3-CMC**, **4-CMC** and **4-MEAP**) and 4 previously unreported ones (**3-CIC**, **4-MDMB**, **4-MNEB** and **4-MDMP**) for which the emergence on the NSP market is expected to be eminent, were also included in this study. Based on the calculation of pharmacokinetic parameters, half-life and intrinsic clearance, **4-CMC** and **4-MDMB** are low and high clearance compounds, respectively, and all the remaining cathinones included in this study are intermediate clearance compounds. This fact anticipates the key role of metabolites as suitable biomarkers to extend detection windows beyond those provided by the parent cathinones. Reduction of the keto group and hydroxylation on the alkyl chains were the common metabolic pathways identified for all cathinones. However, the relative importance of these metabolic transformations is dependent on the cathinone substituents. The glucuronic acid conjugation to metabolites stemming for keto group reduction constituted the sole Phase II transformation identified. To our knowledge, this study constitutes the first metabolite profiling of the already reported synthetic cathinones **4-CIC**, **3-CMC** and **4-CMC**. Noteworthy is the fact that **3-CMC** accounts for almost a quarter of the quantity of powders seized during 2020. The analytical methods developed, and the metabolites characterized, are now available to be included in routine screening methods to attest the consumption of the 8 cathinones studied.

## Introduction

The emergence of new psychoactive substances (NPS) in the recreational drug market constitutes a risk in terms of public health and pose challenges at forensic and health contexts. This prompt changes in drug policies (EMCDDA, 2020) which have already led to the reduction of number of first detections of NPS. However, 52 novel NPS were still first reported in Europe in 2021, and 880 NPS are currently monitored (EMCDDA, 2022a).

Synthetic cathinones constitute the first largest group of NPS seized in Europe, and are the second largest group in terms of the number of controled substances. At the end of 2021, the European Monitoring Centre for Drugs and Drug Addiction (EMCDDA, 2020) was monitoring 162 cathinones (EMCDDA, 2022a; EMCDDA, 2022b). Notably, the quantity of seized cathinones powders have been increasing in recent years. In 2020, cathinones constituted 65% of the material seized (3.3 tonnes) and large seizures have been reported in 2021 and 2022 (EMCDDA, 2022a; EMCDDA, 2022b).

The stimulant effects of these β-keto phenethylamines resemble that of methamphetamine, cocaine, or MDMA (ecstasy) (Baumann et al., 2018). The low price of these recreational drugs together with their toxic effects have led to increased acute intoxications and deaths (La Maida et al., 2021). However, and despite their health risk, most of the new cathinones often remain undetected/uncontrolled by routine drug screening methods (Ellefsen et al., 2016). This reflects the unavailability of suitable analytical methods and reference standards of the parent cathinone, and of its metabolites, to support forensic and clinical identifications. Thus, the large number of emerging cathinones makes difficult updating synthetic cathinone’s information by legal authorities. Additionally, the metabolic degradation of these substances adds one additional layer of difficulty for the legal/clinical control of these NPS. This is more critical in the case of cathinones that undergo extensive/fast metabolic degradation, for which the identification of metabolites in biofluids constitutes the only possible way of attesting their consumption, in forensic and clinical contexts (Uralets et al., 2014). In fact, metabolites can act as consumption biomarkers, extending the detection window beyond that allowed by the parent cathinone. Additionally, the metabolite profile can also shed some light into the mechanisms of toxicity, thereby opening avenues for the development of effective therapeutic options for the management of non-fatal intoxication cases. Therefore, pharmacokinetic information about this class of NPS is key at clinical and legal contexts.

**FIGURE 1:**
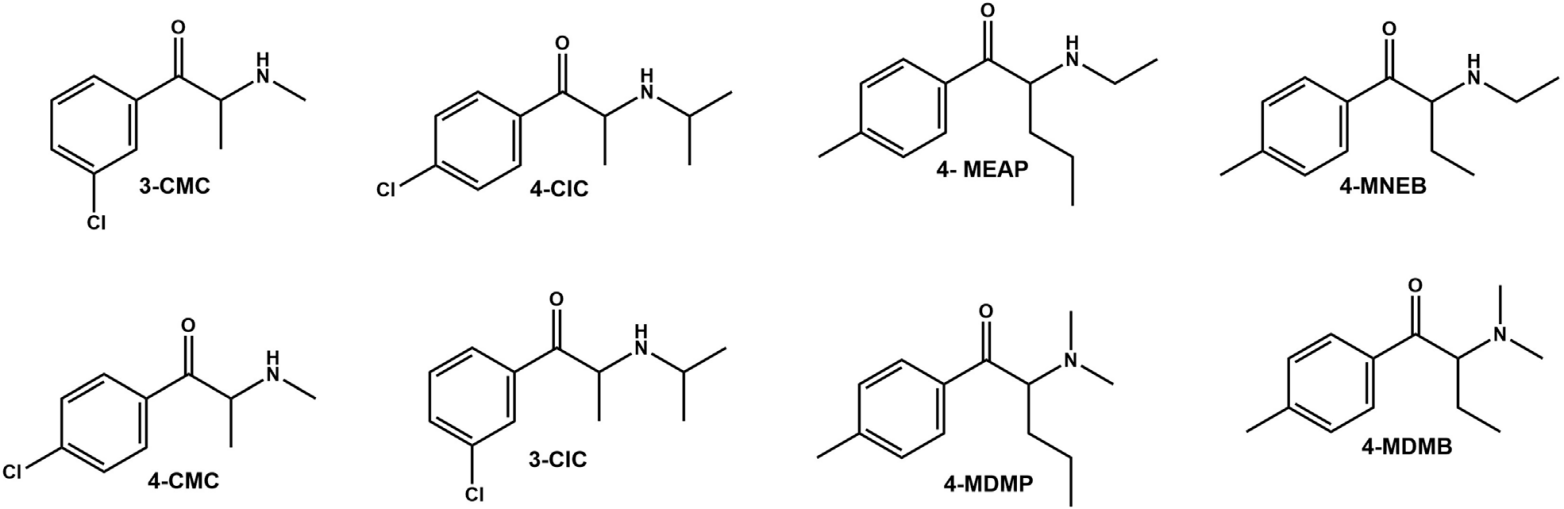
Structures of the **cathinones** used in the current study.

As part of our program aimed at tackling the NPS problem, we decided to determine the metabolic stabilities and metabolite profiles of multiple cathinones, using high resolution mass spectrometry (HRMS). The synthetic cathinones included in this study were (Figure 1): 3′-chloro-*N*-isopropylcathinone (**3-CIC**), 4′-chloro-*N*-isopropylcathinone (**4-CIC**), 3-chloromethcathinone (**3-CMC**), 4′-chloromethcathinone (**4-CMC**), 4′-methyl-*N*-ethylnorpentedrone (also known as 4-methyl-α-ethylamino pentiophenone, **4-MEAP**), 4′-methyl-*N*-ethylbufedrone (**4-MNE B**), 4′-methyl-*N*,*N*-di methylpentedrone (**4-MDMP**), and 4′-meth yl-*N*,*N*-dimethy lbufedrone (**4-MDMB**).

From the selected cathinones, only for **4-MEAP** it was already reported its metabolite profile (Benedicte et al., 2020; Manier et al., 2020). However, pharmacokinetic data on its metabolic stability is yet to be provided. **4-MEAP** was first reported in Luxemburg in 2014, and since then have been related to some fatality cases (Varma et al., 2017). Additionally, **4-CMC** is a drug covered by the 1971 United Nations Convention on Psychotropic Substances, and, **3-CMC** and **4-CIC** have already been reported in Europe (EMCDDA-Europol, 2015; EMCDDA-Europol, 2017; EMCDDA, 2021). Of note is the fact that the halogenated methcathinone **4-CMC** is related to a series of overdose cases (Tomczak et al., 2018; WHO, 2019). Its isomer, **3-CMC**, accounted for almost a quarter of the quantity of cathinones seized during 2020 in Europe (EMCDDA, 2022a; EMCDDA, 2022b) and in 2022, the EU adopted a proposal to control this NPS, based on risk assessments (EMCDDA, 2022c; EMCDDA, 2022d). The other four synthetic cathinones selected for this study (**3-CIC**, **4-MDMB**, **4-MNEB** and **4-MDMP**) are yet to be reported. The choice of these cathinones was based on the following arguments: 1) **3-CIC** is a new compound that is an isomer of the already reported **4-CIC**; 2) **4-MDMP** is a dimethylcathinone isomeric structure of the already reported **4-MEAP**; 3) **4-MNEB** is structurally similar to the already reported **4-MEAP**, but lacking one carbon at the alkyl substituent; and 4) **4-MDMB** is *N*,*N*-dimethyl isomer of **4-MNEB**. The emergence in the NSP market, of these 4 previously unreported cathionones, is expected to be eminent, due not only to their structural resemblance to other already reported synthetic cathinones but also due to their easy synthesis, from available raw material.

## Material and methods

### Chemicals and biochemicals

Human liver microsomes (HLM) and Vivid^®^ regeneration system were obtained from Thermo Fisher Scientific-Gibco. All other commercially available reagents were acquired from Sigma-Aldrich Química, S.A. and used as received.

The hydrochloride salts of all synthetic cathinones used in this study were obtained by synthetic procedures (Kalendra et al., 2003; Santali et al., 2011; Julio et al., 2022). The 4-methylcathinones selected for this work, **4-MDMB, 4-MNEB**, **4-MDMP**, and **4-MEAP**, were synthesized under the scope of our previous work (Julio et al., 2022), while chloro-cathinones were prepared for this study by synthetic procedures reported in literature (Kalendra et al., 2003; Santali et al., 2011; Julio et al., 2022). Briefly, **3-CMC**, **4-CMC**, **3-CIC**, **4-CIC**, and **bupropion** in the form of free base were obtained upon addition of the appropriate amine to 3-chloro-bromoketone or 4-chloro-bromoketone. These intermediate compounds were synthesized from the respective starting ketones (3-chloroketone or 4-chloroketone) by bromination under acidic catalysis (Kalendra et al., 2003; Santali et al., 2011; Julio et al., 2022). To obtain **3-CMC** and **4-CMC**, a 2 M solution of dimethylamine in tetrahydrofuran (THF) was added to the respective bromoketone dissolved in dry THF (14 mmol of amine to 3.4 mmol of bromoketone) (Hyde et al., 1928; McDermott et al., 2011; Julio et al., 2022). For **3-CIC**, **4-CIC**, and **bupropion** the proper cooled 50% (V/V) solution of isopropylamine or *tert*-butylamine in dry THF was added dropwise to the respective bromoketone and maintained in an ice bath during the addition (1 mmol of bromoketone to 2 mmol of amine) (Hyde et al., 1928; Julio et al., 2022). The reaction mixtures were left overnight, and the respective workup was performed as previously described (Santali et al., 2011; Julio et al., 2022). The afforded free base cathinones were treated with 3 mol dm^−3^ ethereal HCl until the precipitation of solid ceased. The resulting precipitates were purified by successive washes with diethyl ether and cooled acetone (Meltzer et al., 2006; Julio et al., 2022), yielding the desired hydrochlorides salts of **3-CMC**, **4-CMC**, **3-CIC**, **4-CIC**, and **buproprion** a yield of 17%, 21%, 78%, 65%, and 48%, respectively. The purity of **4-MDMB, 4-MNEB**, **4-MDMP**, and **4-MEAP** (94%–98%) was assessed through the GC-FID methodology, as previously described (Julio et al., 2022). The purity of **3-CMC** (99%), **4-CMC** (97%), **3-CIC** (90%), and **4-CIC** (93%) was determined by the same methodology. The NMR data obtained in DMSO-*d*_6_, for **4-CMC** and bupropion, was in accordance with previously reported data (Perrine et al., 2000; Taschwer et al., 2014; Khan et al., 2016; Nycz et al., 2016). Non-etheless, since the full ^1^H and ^13^C NMR assignments in DMSO-*d*_6_ for **3-CIC, 4-CIC** and **3-CMC** were yet to be presented, they are reported herein for the first time (Supplementary Table S1).

### Instrumentation

LC-HRMS/MS analyses were performed on Elute UPLC system (Bruker, Bremen, Germany) connected with a Bruker Impact II quadrupole time-of-flight mass spectrometer equipped with an ESI source (Bruker Daltoniks, Bremen, Germany). For the chromatographic separations a Luna C18 (2) column (150 mm × 2.0 mm inner diameter; 3.0 µm particle size, Phenomenex) equipped with a C18 (Phenomenex) guard disk (2 × 4 mm) was used. The mobile phase consisted of 0.1% formic acid in water (mobile phase A) (pH 2.6) and 0.1% formic acid in acetonitrile (mobile phase B) at a flow rate of 170 μL/min. A 15 min gradient was used as follows: 5%–50% B for 6 min; 50%–100% B for 4 min; isocratic elution with 100% B for 5 min; 100%–5% B for 4 min; and finally, 5% B for 9 min; The injection volume was 10 μL. The column and the autosampler were maintained at 40°C and 8°C, respectively. HRMS spectra were acquired in the positive electrospray ionization modes ESI (+) and ESI (−) using the mass spectrometric conditions previously described (Lopes et al., 2021). For raw data processing DataAnalysis 4.1 software (Bruker Daltoniks) was used.

The ^1^H NMR spectra of chlorocathinones hydrochloride salts were performed on a Bruker Avance spectrometer, in DMSO-*d*_6_, operating at 400.1 MHz. ^13^C NMR were obtained on the same instrument at 100.6 MHz. Unequivocal assignments of all proton and carbon signals were achieved by 1D (^1^H, ^13^C APT) and 2D (COSY, HMBC and HSQC) NMR experiments. Chemical shifts (δ) are reported in ppm and referenced to the DMSO-*d*_6_ signal (δ_H_ = 2.50 ppm and δ_C_ = 39.50 ppm); coupling constants are reported in Hz.

### Cathinones incubation with human liver microsomes

#### Generation of Phase I metabolites

For the generation of Phase I metabolite, the selected cathinones were incubated in human liver microsomes (HLM) by adaptation of a previous described method (Lopes et al., 2021). Briefly, selected synthetic cathinones, at a concentration of 10 μM, were incubated with HLM (1 mg/mL), Vivid^®^ regeneration system (1 μL), NADPH (1 mM, 1 μL), for a total incubation volume of 500 μL in 50 mM ammonium bicarbonate (ABIC) buffer at pH 7.4. Each incubation was run in duplicate. Control incubations were conducted in the same conditions: 1) using water as a negative control, in the absence of cathinone; 2) in the absence of the NADPH cofactor; 3) using heat-denatured (90°C, 15 min) microsomes; and 4) using bupoprion, as a positive control incubation, to attest the experimental conditions used. The mixtures were incubated at 37°C and 50 μL aliquots were collected following at 0, 5, 10, 15, 20, 25, 30, 45, 60, 75, 90, 120, and 180 min of incubation (except for 3-CMC, for which the 180 min time-point was not collected). A cold 2.5 µM reserpine solution (50 μL, internal standard) in acetonitrile was then added to each aliquot to quench the reactions. Following centrifugation at 10,000 *g* for 15 min at room temperature, the supernatants were collected and analyzed by LC-HRMS/MS.

#### Generation of Phase II metabolites

For the generation of Phase II metabolites, alamethicine-induced HLM (Fisher et al., 2000) were used in the presence of Phase I and and glucuronidation co-factor. Briefly, HLM (1 mg/mL) were preincubated for 15 min, in ice, with alamethicin (25 μg/mL) in 50 mM ABIC buffer at pH 7.4, for a total incubation volume of 200 μL. Following the addition of MgCl_2_ (2 mM; 1 μL) and selected cathinone (10 μM final concentration), the resulting solution was incubated for 5 min at 37°. NADPH (5 mM) and UDPGA (5 mM) were then added to start the Phase I and II reactions. Incubations were run in duplicate. Additionally, the following control incubations were conducted in the same conditions: 1) Negative control, using water in the absence of cathinone; 2) Cofactor control, run in the absence of the NADPH and UDPGA; 3) Denatured control, using heat-denatured (90°C, 15 min) microsomes; and 4) Positive control using, **bupoprion** instead of the selected cathinones, to attest the viability of the experimental conditions used. The mixtures were incubated at 37°C and a 100 μL aliquot was collected following 2 h of incubation A cold 2.5 µM reserpine solution (100 μL, internal standard) in acetonitrile was then added to each aliquot to quench the reactions. Following centrifugation at 10,000 *g* for 15 min at room temperature, the supernatants were collected and analyzed by LC-HRMS/MS.

### Metabolic stability calculations

Following LC-ESI-HRMS/MS analysis of aliquots obtained from Phase I, a depletion plot was obtained by plotting the time of incubation against the natural logarithmic of the relative area (the peak area ratio of the cathinone and of the internal standard, reserpine). From the linear regression through the initial (linear) part of this curve it was obtained the slope (k) that was then used in Eq. 1, for the calculation of the half-life, assuming a first-order kinetic trend.

$$t_{1/2}=\frac{\ln2}{k}$$ (1)

The intrinsic clearance (CL_int_) was calculated by Eq. 2 (McNaney et al., 2008), using 26 g of liver per kg b.w. (Słoczyńska et al., 2019):

$${CL}_{\mathrm{int}}=\frac{\ln2}{t_{\frac{1}{2}}}\;x\;\frac{mL\;of\;incubation}{mg\;of\;protein}\;x\;\frac{45\;mg\;of\;microsomal\;protein}{g\;of\;liver\;weight}\;x\;\frac{g\;of\;liver\;weight}{Kg\;b.w.}$$ (2)

### Metabolite identification

An in-house accurate mass library was first built, considering the metabolic pathways already known for other structurally similar cathinones, and a targeted screening was then carried out by performing extracted ion chromatograms (EIC), with a mass window of ±5 ppm, for the protonated/deprotonated molecules of the expected metabolites in the full scan spectra. Isotope cluster analysis was also used to identify chlorinated metabolites. All spectra corresponding to metabolites were then manually checked. The mass deviation from the accurate mass of the identified cathinone metabolites remained below 5 ppm for the precursor and below 10 ppm for product ions. Candidates present in control incubations and/or without MS/MS data were excluded. The MS/MS spectra of the selected cathinones and their identified metabolites are displayed in the Supplementary Material (Supplementary Figures S1–S54). Metabolites were ranked based on their relative abundance over incubation time (Figures 2–9). The relative abundance was calculated by the ratio of the relative area of each metabolite and the sum of the relative areas obtained for all metabolites and for the respective parent cathinone.

**FIGURE 2:**
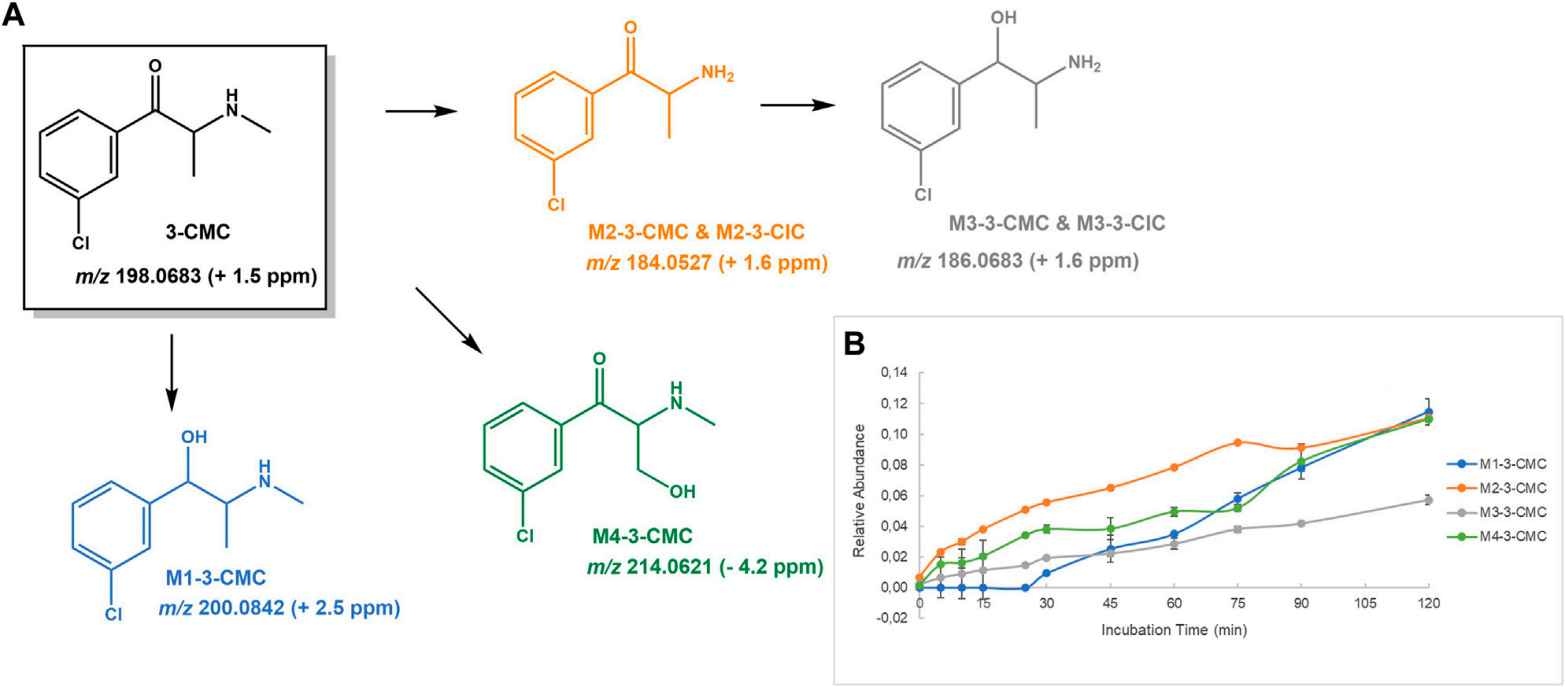
**(A)** Proposed structures of the **3-CMC** Phase I metabolites identified by LC-HRMS/MS analysis in HLM incubation; and **(B)** Relative abundance of **3-CMC** Phase I metabolites over HLM incubation time.

**FIGURE 3:**
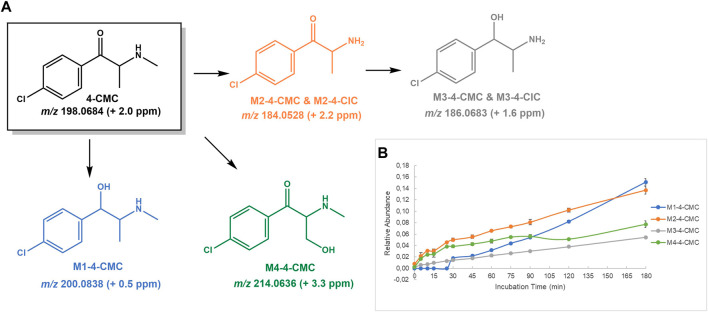
**(A)** Proposed structures of the **4-CMC** Phase I metabolites identified by LC-HRMS/MS analysis in HLM incubation; and **(B)** Relative abundance of **4-CMC** Phase I metabolites over HLM incubation time.

**FIGURE 4:**
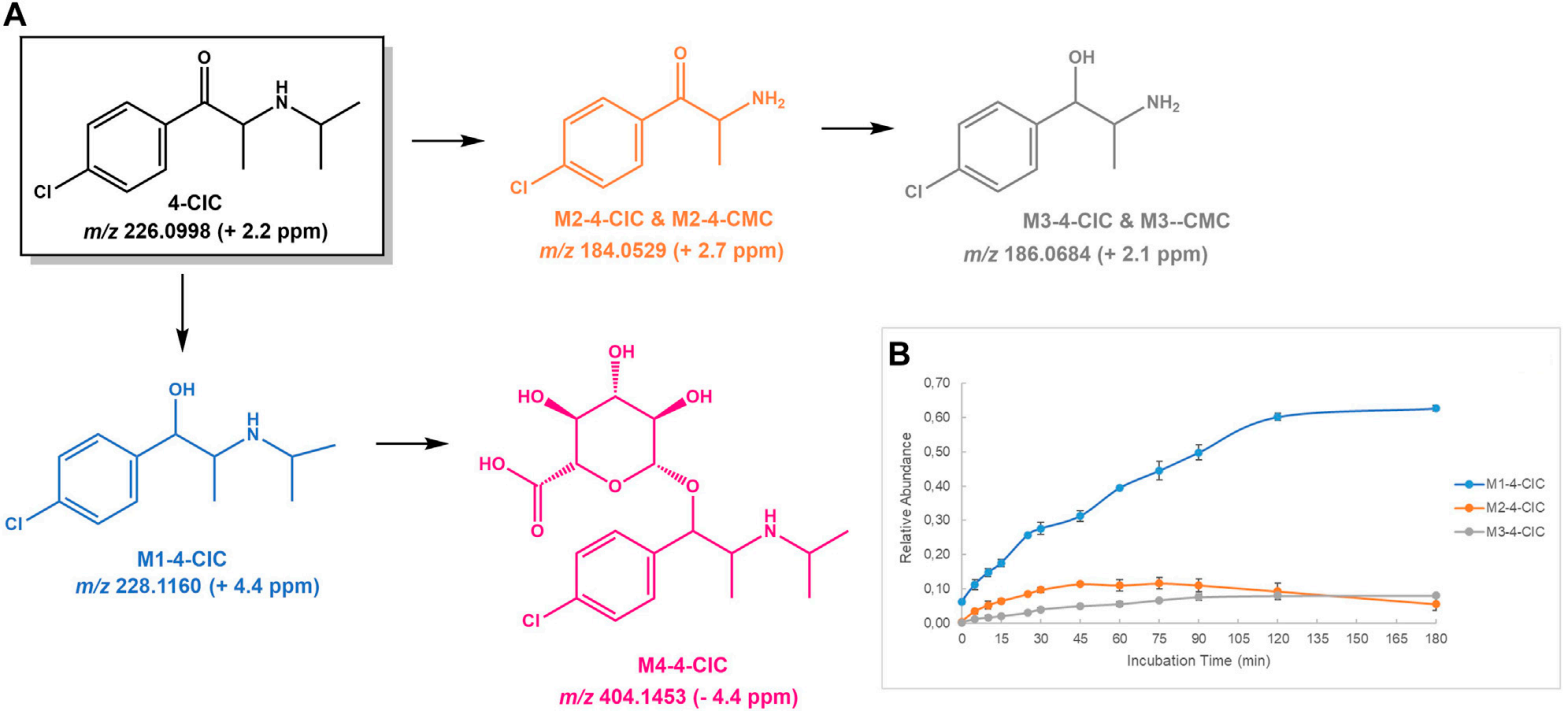
**(A)** Proposed structures of the **4-CIC** Phase I and II metabolites identified by LC-HRMS/MS analysis in HLM incubation; and **(B)** Relative abundance of **4-CIC** Phase I metabolites over HLM incubation time.

**FIGURE 5:**
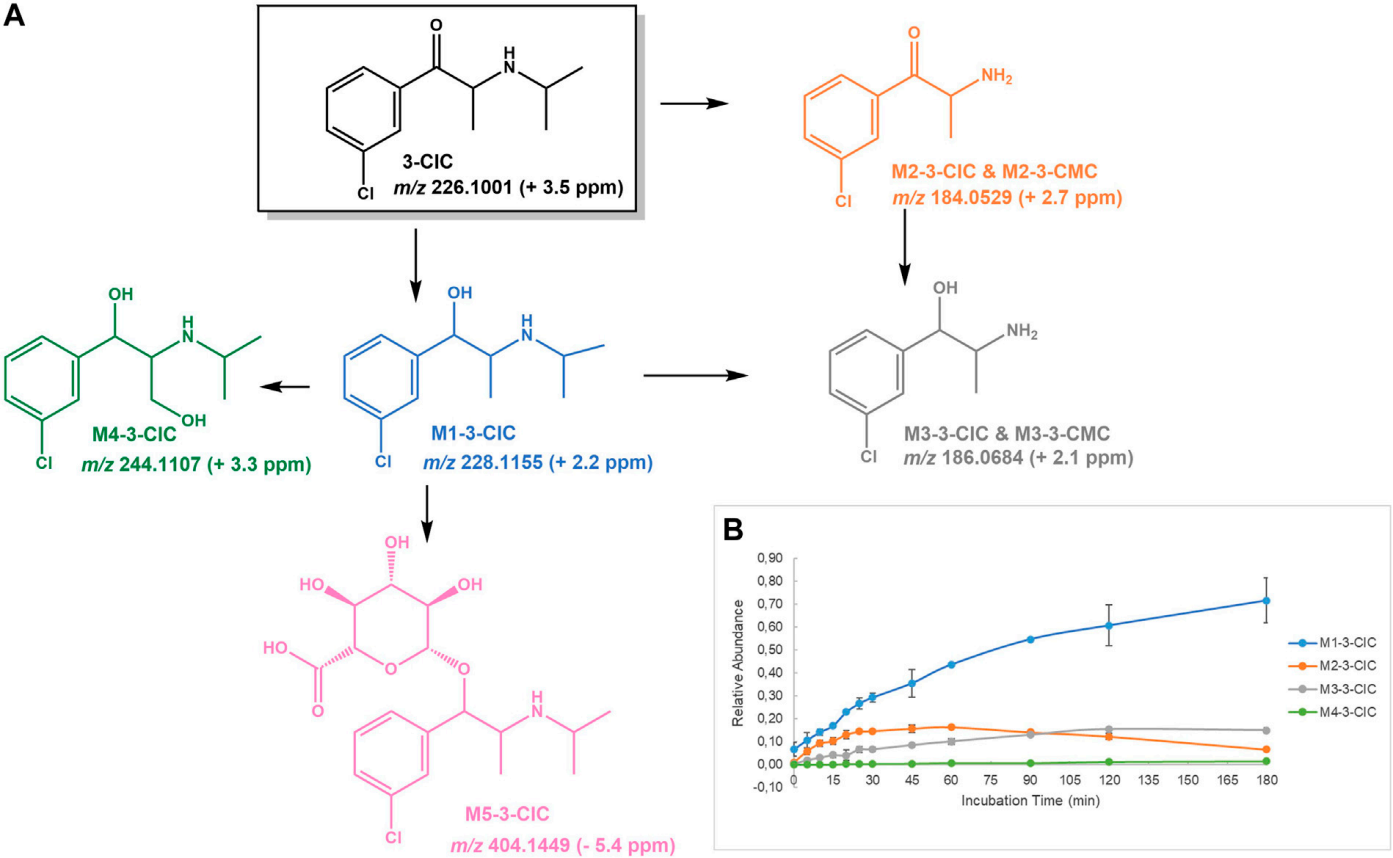
**(A)** Proposed structures of the **3-CIC** Phase I and II metabolites identified by LC-HRMS/MS analysis in HLM incubation; and **(B)** Relative abundance of **3-CIC** Phase I metabolites over HLM incubation time.

**FIGURE 6:**
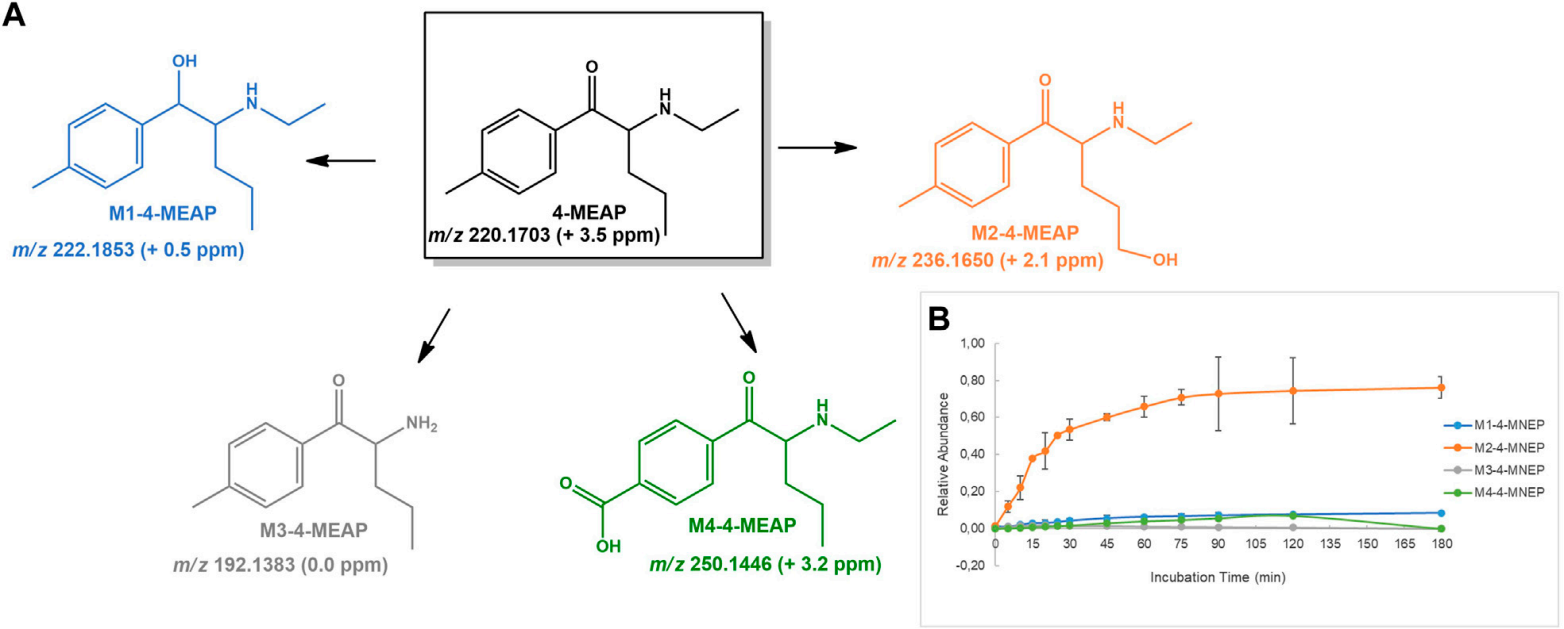
**(A)** Proposed structures of the **4-MEAP** Phase I metabolites identified by LC-HRMS/MS analysis in HLM incubation; and **(B)** Relative abundance of **4-MEAP** Phase I metabolites over HLM incubation time.

**FIGURE 7:**
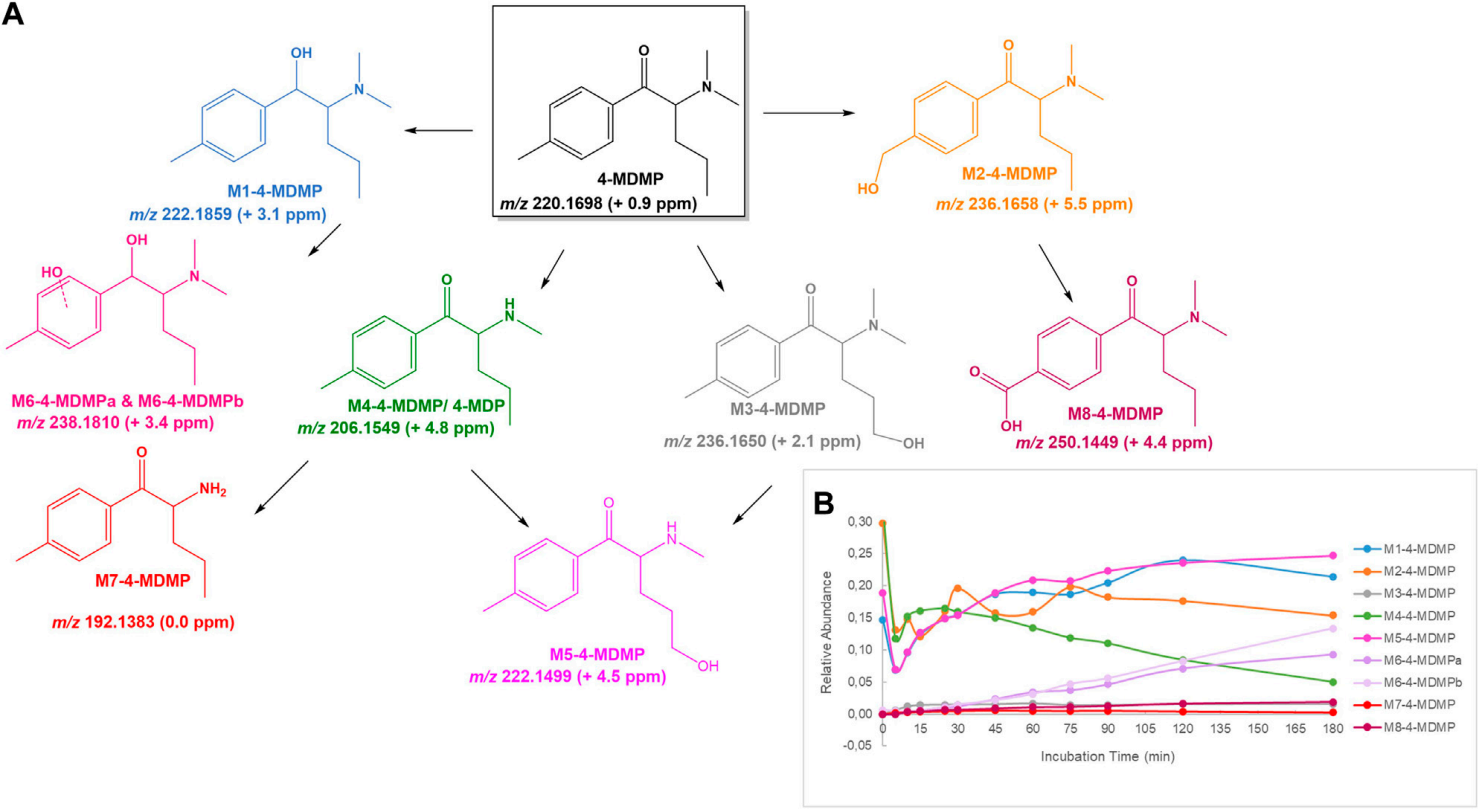
**(A)** Proposed structures of the **4-MDMP** Phase I metabolites identified by LC-HRMS/MS analysis in HLM incubation; and **(B)** Relative abundance of **4-MDMP** Phase I metabolites over HLM incubation time.

**FIGURE 8:**
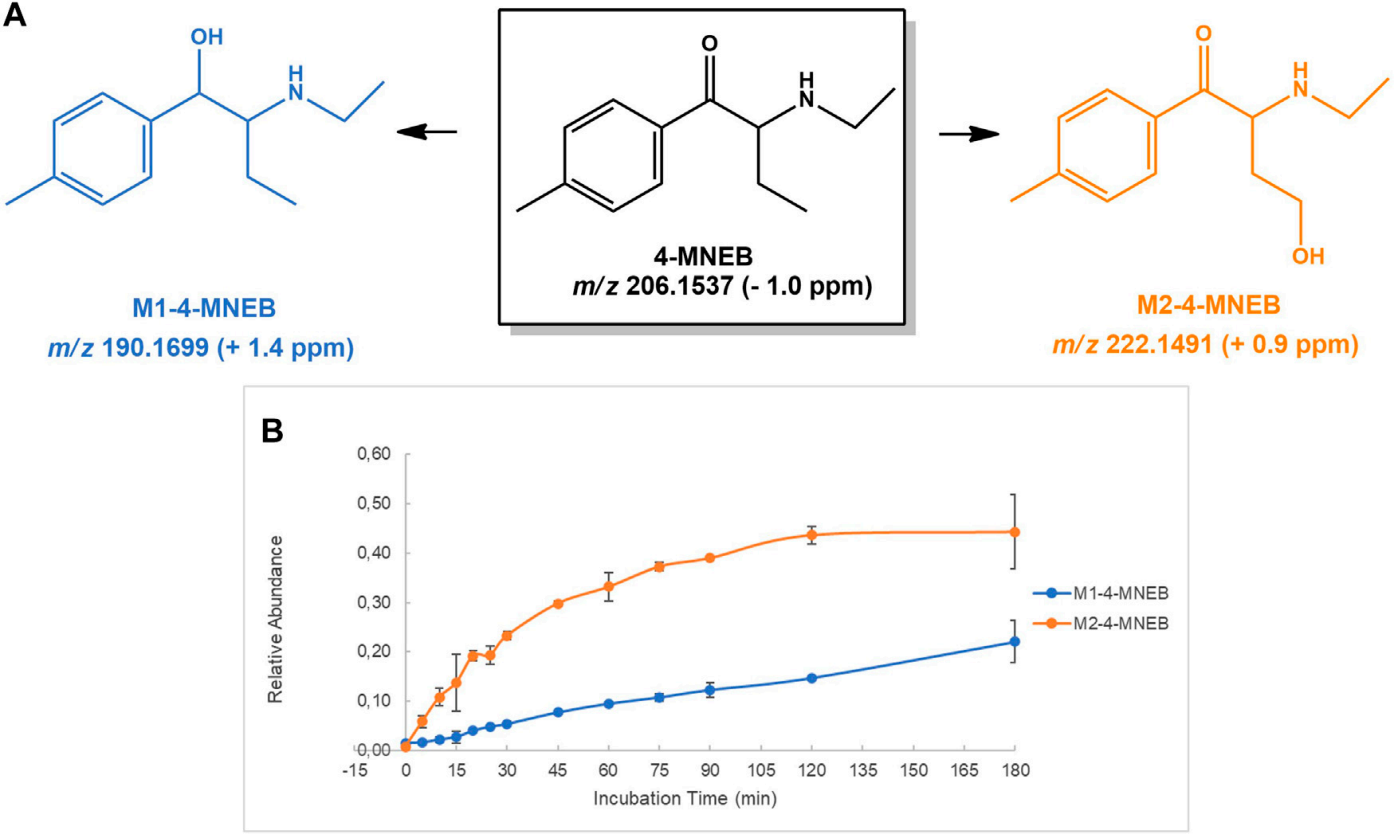
**(A)** Proposed structures of the **4-MNEB** Phase I metabolites identified by LC-HRMS/MS analysis in HLM incubation; and **(B)** Relative abundance of **4-MNEB** Phase I metabolites over HLM incubation time.

**FIGURE 9:**
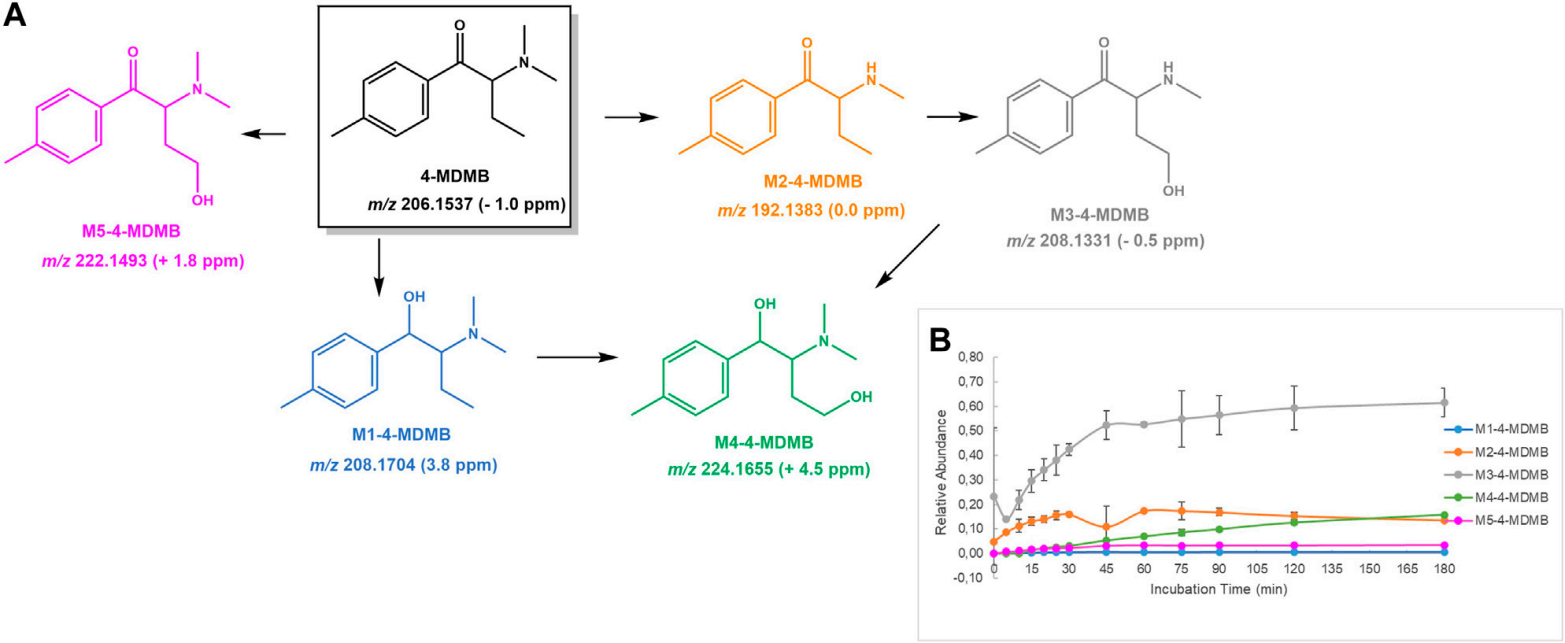
**(A)** Proposed structures of the **4-MDMB** Phase I metabolites identified by LC-HRMS/MS analysis in HLM incubation; and **(B)** Relative abundance of **4-MDMB** Phase I metabolites over HLM incubation time.

## Results and Discussion

### Metabolic stability and pharmacokinetics parameters

Metabolic stability, which gives a measure of the susceptibility to biotransformation, can be estimated from HLM incubations-based half-life and intrinsic clearance calculations.

The results obtained for all cathinones included in the study are displayed in Table 1. According to the scoring proposed by McNaney et al. (2008), based on t_1/2_ values, **4-CMC** is estimated to be a low clearance compound (t_1/2_ > 60 min), **4-MDMB** is a fast clearance compound (t_1/2_ < 20 min) and all the remaining cathinones included in this study are intermediate clearance compounds (60 ≥ t_1/2_ ≥ 20 min). Interestingly, 3-chloro substituted cathinones displayed lower half-lives, when compared with their corresponding 4-chloro isomers. Additionally, all tested cathinones presented lower half-lives than the one reported for **4-CEC** (t_1/2_ 105 min) (Wagmann et al., 2020), thereby suggesting their higher liability towards metabolic degradation. Despite the unavailability of *in vivo* pharmacokinetic data for cathinones selected for this study, the low clearance label obtained for **4-CMC** contrasts with a forensic report, where this cathinone is suggested to have a very fast metabolism (Wiergowski et al., 2017). This is in line with Chiba et al.(2009), suggestion that *in vitro* metabolic clearance obtained from HLM under-predicts the *in vivo* metabolic clearance. Therefore, the results obtained anticipate the key role of metabolites as suitable biomarkers to extend detection windows beyond those provided by parent cathinone.

**TABLE 1:** Pharmacokinetic parameters, half-life (t_1/2_) and intrinsic clearance (CL_int_), calculated for the 8 cathinones selected for this study, obtained in HLM incubations.

	t_1/2_ (min)	CL_int_ (mLmin^−1^ kg^−1^)
**3-CIC**	24	34
**4-CIC**	36	22
**3-CMC**	54	15
**4-CMC**	78	10
**4-MEAP**	24	33
**4-MNEB**	55	16
**4-MDMP**	46	18
**4-MDMB**	15	54

### Metabolite profiling

As already stated, the metabolite profiling of emerging cathinones is key towards the identification of potential consumption biomarkers. *In vitro* studies using HLM, have demonstrated their efficiency in generating the major metabolites identified *in vivo* for several cathinones. This justifies the ample use of this the cost-effective, easy to implement, metabolic competent system, to characterize the Phase I and Phase II (glucuronidation) metabolite profile of synthetic cathinones (Meyer et al., 2010; Meyer and Maurer, 2010; Meyer et al., 2012). High resolution mass spectrometry-based QTOF technology constitutes one the most powerful analytical platforms for the screening and identification of new drug metabolites, in general, and of cathinones, in particular (Helfer et al., 2015; Manier et al., 2018). Therefore, we decided to use this analytical methodology to identify the Phase I and II (glucuronidation) metabolite profiles, generated in HLM incubations, for the 8 selected cathinones. Thus, in addition to Phase I hydroxylation of alkyl and aromatic positions, reduction of β-keto group and *N*-dealkylation, glucuronidation constitutes the main Phase II conjugation pathway reported for cathinones (Zaitsu, 2018). In either instance, incubations with bupropion were performed to attest the viability of the experimental conditions used to generate the major metabolites of cathinones *in vivo* (Supplementary Figure S1). This strategy was used since bupropion is a synthetic cathinone in clinic use as antidepressant, and, therefore, its metabolic profile is extensively studied (Costa et al., 2019). In particular, the identification of the Phase I metabolites stemming (Supplementary Figures S2–S4) from the reduction β-keto group (**M2-buproprion**) and hydroxylation of the *t*-butyl moiety, followed by the intramolecular hemiacetal formation (**M3-bupropion**), in incubations attested the viability of the experimental conditions used to generate the major metabolites of this cathinone reported *in vivo*. The identification of **M4-buproprion**, the glucuronide conjugate of **M1-bupropion**, further substantiated this viability.

A direct relationship between the relative intensities of electrospray MS signals generated by electrospray ionization (ESI) and the effective concentration of the multiple metabolites formed in incubations cannot be obtained. This stems not only from potential distinct abilities to ionize but also from the unavailability of synthetic standards of the multiple metabolites. Non-etheless, the use of an internal standard allowed us to follow the relative abundance of the parent cathinone and of their metabolites over time, thereby providing a picture of their metabolic fate. A full characterization of the parent cathinones was performed by HRMS/MS (*c.f.* Supplementary Material), which was very valuable for the identification of their metabolite profiles.

#### 3-CMC

**3-CMC** is not only associated to the increase of cathinones powders seized in Europe observed during the pandemic year of 2020, but also to multiple fatal and non-fatal intoxication cases. This prompt the EMCDDA to issue a warning on the risk posed by this cathinone (EMCDDA, 2022c; EMCDDA, 2022d). While *in vivo* pharmacokinetic data are yet to be reported for **3-CMC**, a relatively extensive metabolic degradation can be inferred from the half-life and intrinsic clearance values obtained in the current study (Table 1). The following Phase I metabolic pathways were identified in **3-CMC** HLM incubations (Figure 2): 1) Reduction of the β-keto group, yielding **M1-3-CMC**; 2) *N*-demethylation, affording **M2-3-CMC**, followed by reduction of the β-keto group, yielding **M3-3-CMC**; and 3) Hydroxylation of the *N*-methyl substituent to afford **M4-3-CMC**. No Phase II metabolites were identified for this cathinone in incubations run with alamethecin-induced HLMs in the presence of UDPGA.

**M1-3-CMC** presents a mass increment of 2 u, when compared with the parent cathinone, which is compatible with the product obtained upon reduction of β-keto group. The protonated molecule of this metabolite is observed at *m/z* 200.0842 (+2.5 ppm) and the base peak of its tandem mass spectrum (Supplementary Figure S9) is observed at *m/z* 182.0738 (+3.8 ppm), stemming for water loss. The subsequent loss of HCl and methyl amine yields the fragment ion at *m/z* 115.0544 (+1.7 ppm).

The protonated molecule of the *N*-demethylated metabolite, **M2-3-CMC** is observed at *m/z* 184.0532 (+4.3 ppm) and shows a fragmentation mechanism (Supplementary Figure S10) very similar to the one suggested by Pozo et al. (2015) for the mephedrone’s *N*-demethylation metabolite, involving three main fragmentation mechanisms; 1) the formation of an indole ring, afforded as the result of an intramolecular rearrangement accompanied with water loss, yielding the fragment ions at *m/z* 166.0420 (+1.2 ppm), which upon loss of chlorine radical, yields the fragment ion at *m/z* 131.0727 (−2.3 ppm); 2) the consecutive neutral losses of NH_3_ and CO from the parent protonated molecule, yields the fragment ion at *m/z* 139.0310 (+0.7 ppm), which upon subsequent loss of HCl yields the fragment ion at *m/z* 103.0541 (−1.0 ppm); and 3) the consecutive neutral losses of oxygen, NH_3_ and HCl from the parent protonated molecule, yielding the fragment ions at *m/z* 168.0577 (+1.2 ppm), 151.0305 (−2.6 ppm) and 115.0544 (+1.7 ppm).

The protonated molecule of **M3-3-CMC** is displayed at *m/z* 186.0683 (+1.6 ppm), which is compatible with product of two metabolic steps of *N*-demethylation and reduction. The base peak of its tandem mass spectrum is observed at *m/z* 168.0583 (+4.2 ppm), stemming from the water loss from the parent protonated molecule. This product ion undergoes two parallel fragmentation pathways (Supplementary Figure S11): 1) Two subsequent neutral losses of NH_3_ and HCl, yielding the product ion at *m/z* 115.0544 (+1.7 ppm); and 2) intramolecular rearrangement, (Pozo et al., 2015), followed by the loss of the chlorine radical, which explains the formation of the fragment ion at *m/z* 131.0733 (+3.1 ppm). **M4-3-CMC** presents a mass increment of 15.9944 u from the protonated molecule of the parent cathinone and is observed at *m/z* 214.0621 (−3.7 ppm), which is compatible with the occurrence of a hydroxylation. The location of the hydroxyl group at the aromatic position is discarded by the observation of the fragment ion at *m/z* 138.9951 (+4.3 ppm), corresponding to the oxonium ion, which corresponds to the base peak of its tandem mass spectrum (Supplementary Figure S12). Whereas the location of the hydroxyl substituent at the methyl alkyl chain is suggested for this metabolite, its location at the *N*-methyl substituent cannot be discarded based on the lack of further diagnostic fragment ions.

#### 4-CMC

**4-CMC** is associated with multiple and serious adverse effects (Grifell et al., 2017; Tomczak et al., 2018). Whereas to the best of our knowledge the present study constitutes the first report on the metabolite profile of this drug, its rapid metabolism can be inferred by a previous *in vivo* report (Wiergowski et al., 2017), which anticipates the usefulness of its metabolites as potential biomarkers of consumption. As expected, the metabolite profile of this cathinone was very similar to the one observed for its isomer, **3-CMC**. Four Phase I metabolite were identified (Figure 3): 1) at *m/z* 200.0838 (+0.5 ppm), **M1-4-CMC**, stemming from reduction of the 4-CMC keto group; 3) at *m/z* 184.0528 (+2.2 ppm), **M2-4-CMC**, the metabolite formed by *N*-demethylation, which subsequently affords **M3-4-CMC** upon reduction of α-keto group, observed at *m/z* 186.0683; and 3) at *m/z* 214.0636 (3.3 ppm), **M4-4-CMC**, the metabolite afforded upon hydroxylation of the methyl side chain. Similarly, to what was observed for the isomer **3-CMC**, no Phase II metabolite was identified in the HLM incubations of **4-CMC** run in the presence of UDPGA.

The base peak of the tandem mass spectrum of the protonated molecule of **M1-4-CMC** (at *m/z* 200.0838) corresponds to the neutral loss of water and is observed at *m/z* 182.0734 (+3.8 ppm) (Supplementary Figure S15). Two other fragment ions are observed at *m/z* 145.0883 (−2.1 ppm) and 115.0542 (0.0 ppm), which are also observed in the corresponding **M1-3-CMC** isomer. The protonated molecule of **M2-4-CMC** (at *m/z* 184.0528) displays three main fragmentation pathways (Supplementary Figure S16): 1) the neutral losses of water, NH_3_ and HCl to produce the fragment ion at *m/z* 115.0548 (+5.2 ppm); 2) the neutral of CO and NH_3_ yields the fragment ion at *m/z* 139.0315 (4.3 ppm), which upon the subsequent loss of HCl leads to the fragment ion at *m/z* 103.0543; and 3) the concomitant intramolecular rearrangement with water loss yields the indolic fragment ion at *m/z* 166.0426 (+4.8 ppm), which upon loss of chlorine radical yields the ion at *m/z* 131.0733 (+2.3 ppm). The base peaks of the tandem mass spectrum of **M3-4-CMC** (at *m/z* 186.0683) are observed at *m/z* 168.0575 (0.0 ppm) and 115.0540 (−1.7 ppm) and are explained by the loss of water from the protonated molecules followed by the losses of NH_3_ and HCl, respectively (Supplementary Figure S17): One additional main fragment ion is observed at *m/z* 131.0728 (−1.5 ppm) and can be explained by an intramolecular rearrangement (Pozo et al., 2015). **M4-4-CMC** (Supplementary Figure S18) presents a mass increment compatible with a hydroxylation product observed at *m/z* 214.0636 (+3.3 ppm). Similarly, to what was observed to the corresponding **M4-3-CMC** isomer (Supplementary Figure S12), the location of the hydroxyl substituent at the aromatic moiety is discarded by the observation of the oxonium fragment ion at *m/z* 138.9949 (+2.9 ppm). However, we cannot accurately determine whether hydroxylation occurred on the alkyl chain or on the *N*-substituent, due to the lack of diagnostic ions to support this attribution.

#### 4-CIC

For the already reported NPS **4-CIC**, in addition to **M1-4-CIC**, the metabolite stemming from the reduction of the keto group, two other Phase I metabolites were identified (Figure 4): **M2-4-CIC**, stemming from loss of the *N*-isopropyl substituent, and **M3-4-CIC**, which stems from the loss of *N*-isopropyl substituent from **M1-4-CIC** and/or reduction of **M2-4-CIC**. Noteworthy is the fact that **M2-4-CIC** and **M3-4-CIC** correspond to two metabolites (**M2-4-CMC** and **M3-4-CMC**, respectively) also identified for **4-CMC**. **M4-4-CIC**, formed upon conjugation of glucuronic acid to **M1-4-CIC**, was the sole Phase II metabolite identified for this NPS. Therefore, these results suggest that, in addition to the parent cathinone, only **M1-4-CIC** and its Phase II metabolite **M4-4-CIC**, can act as selective biomarkers of **4-CIC** consumption.

The protonated molecule of **M1-4-CIC** was observed at *m/z* 228.1160 (+4.4 ppm), which presents the expected chlorine isotopic pattern. The initial fragmentation step of this ion corresponds to the loss of water, which affords the fragment ion at *m/z* 210.1053 (+4.3 ppm) (Supplementary Figure S21): The subsequent losses of *N*-isopropyl substituent, NH_3_ and HCl afford the fragment ions at *m/z* 168.0578 (+2.4 ppm), 151.0309 (+0.2 ppm) and 115.0543 (+0.9 ppm), respectively. The Phase II metabolite **M4-4-CIC** is observed at *m/z* 404.1453 (−4.5 ppm) and its tandem mass spectrum exhibits the expected neutral loss of 194.0421 u, (Pozo et al., 2015), corresponding the loss of the glucuronic acid moiety yielding the fragment ion at *m/z* 210.1033 (−5.2 ppm) (Supplementary Figure S22). This fragment ion is then subjected to the same fragmentation pathways observed for **M1-4-CIC**.

#### 3-CIC

As mentioned above, while **3-CIC** to our knowledge this cathinone is yet to be reported in the illicit drug market, the fact that it is an isomer of the already reported **4-CIC** makes its emergence highly probable. Additionally, the easy synthesis of this cathinone from available raw materials further substantiates this possibility. The following metabolic transformations were identified for this cathinone (Figure 5): 1) Reduction of β-keto group to afford **M1-3-CIC**; 2) *N*-dealkylation to afford **M2-3-CIC**, which corresponds to **M2-3-CMC** metabolite identified in **3-CMC** incubations; 3) the two consecutive steps of *N*-dealkylation and β-keto reduction affords **M3-3-CIC**, which corresponds to **M3-3-CMC** also detected in **3-CMC** incubations; 4) Hydroxylation of **M1-3-CIC** yields two metabolites, one of which is **M4-3-CIC** that corresponds to the hydroxylation product at the alpha-methyl position; and 5) the Phase II glucuronic acid conjugation of the Phase I metabolite **M1-3-CIC**, to afford **M5-3-CIC**. Importantly, the metabolite profile identified shows that **M2-3-CIC** and **M3-3-CIC** cannot act as **3-CIC** (or **3-CMC**)-specific biomarkers of exposure.

The protonated molecule **of M1-3-CIC** was observed at *m/z* 228.1155 (−2.3 ppm), and similarly to what was observed for the reduction metabolite of **4-CIC**, the fragmentation pattern of this ion showed the following consecutive neutral losses (Supplementary Figure S25): 1) loss of water, to afford the fragment ion at *m/z* 210.1052 (−3.6 ppm); 2) loss of isopropyl *N*-alkyl substituent, to yield the fragment ion at *m/z* 168.0579 (+2.4 ppm); 3) loss of NH_3_, affording the ion at *m/z* 151.0310 (−0.3 ppm); and 4) loss of HCl, yielding the fragment ion at *m/z* 115.0544 (+1.4 ppm). Interestingly, in addition to the three metabolic pathways identified for **4-CIC**, the **M1-3-CIC** metabolite isomer showed to be also prone to hydroxylation. Coherently, the extracted ion chromatogram at *m/z* 244.1099, showed two very closely eluting signals, whose full scan mass spectrum exhibited the expected chlorine isotopic pattern, compatible with the formation of two isomeric metabolites resulting from the introduction of a hydroxyl group into the reduction product **M1-3-CIC**. Whereas this suggests the formation of two hydroxylation isomers, only for the first eluting metabolite **M4-3-CIC**, it was possible to assign the location of the hydroxyl group, since only this ion was chosen for fragmentation. The observation of the fragment ion at *m/z* 103.0539, which is also observed for most of the chlorinated cathinones and corresponding metabolites, excludes the aromatic position as possible hydroxylation site (Supplementary Figure S26). The base peak of the tandem mass spectrum of this metabolite corresponds to the loss of water from the parent ion, and the diagnostic ion at *m/z* 184.0531 (+3.8 ppm), proposed to be formed upon the subsequent loss of the amine moiety, supports the α-methyl group as the hydroxylation site. This assignment is further corroborated by the observation of the fragment ion at *m/z* 131.0493 (+1.5 ppm), which is proposed to be formed upon subsequent of amine group from the former ion. The additional hydroxylation metabolite identified in incubations might result from the hydroxylation at the *N*-alkyl substituent or at an aromatic position.

#### 4-MEAP

**4-MEAP** have been reported for the first time in 2014 in Luxemburg (EMCDDA-Europol, 2015), and at least two fatal cases associated with (co-)misuse of this cathinone have been described (Varma et al., 2017; Lelièvre et al., 2019). The metabolite profile of **4-MEAP** in HLM have already been described in two independent studies (Benedicte et al., 2020; Manier et al., 2020) and the metabolite profile identified in the current work is in accordance with the metabolic pathways previously reported for this cathinone, comprising (Figure 6): 1) reduction of the cathinone oxo group to **M1-4-MEAP**; 2) hydroxylation of the alkyl chain to **M2-4-MEAP**; 3) *N*-deethylation, to **M3-4-MEAP**; 4) carboxylic acid formation at the benzylic position to **M4-4-MEAP**. The great novelty is the identification of the hydroxylation of the alkyl chain as the main metabolic pathway, which is shown by the comparative relative intensity over the incubation time of each metabolite. Additionally, the fact that no product of benzylic hydroxylation was found in incubations suggests that once formed this metabolite is rapidly oxidized to its oxo derivative, **M4-4-MEAP**. This is in accordance with the fact that Benedict et al. (2020) only identified trace amounts of **M3-4-MEAP** and hydroxy benzyl **4-MEAP** in human urine, collected from a fatal case of intoxication. Therefore, **M4-4-MEAP** is likely to act as a better biomarker of exposure to this cathinone. Also noteworthy is the fact that no glucuronide metabolite was identified for **4-MEAP** in incubations run in the presence of Phase I and Phase II co-factors. This suggests that neither **4-MEAP** nor its Phase I metabolite are prone to this Phase II metabolic transformation.

**M1-4-MEAP**, **M2-4-MEAP** and **M3-4-MEAP** are observed at *m/z* 222.1853 (+0.5 ppm) 236.1650 (+2.1 ppm) and 192.1383 (0.0 ppm), respectively, and their tandem mass spectra present a fragmentation pattern similar to the one described by Manier et al. (2020) for these three metabolites (Supplementary Figures S30–S32). The protonated molecule of the carboxylic acid derivative **M4-4-MEAP** is observed at *m/z* 250.1446 (+3.2 ppm). While its tandem mass spectrum shows all main fragment ions previously described by for this metabolite (Manier et al., 2020), we would like to emphasize on additional fragment ion, at *m/z* 100.1127 (+6.0 ppm) that was not mentioned by Manier et al. (2020). This fragment ion corresponds to the protonated molecule of *N-*ethyl-*N*-but-1-en-1-amine, which is also observed in the tandem mass spectrum of the parent **4-MEAP** and acts as a diagnostic ion for the occurrence of the metabolic transformation at the aromatic moiety.

#### 4-MDMP

Whereas to our knowledge the *N*,*N*-dimethyl cathinone **4-MDMP** is yet to be reported in the drug of abuse market, this cathinone is a structural isomer of **4-MEAP**. In addition to this structural feature its easy synthesis from available raw materials makes its possible emergence probable.

The metabolite profile identified in HLM incubations of this cathinone showed multiple metabolic pathways (Figure 7): 1) β-keto group reduction, yielding **M1-4-MDMP**; 2) Hydroxylation at alkyl chains of aromatic and alpha position moieties, affording **M2-4-MDMP** and **M3-4-MDMP**, respectively; 3) *N*-demethylation to afford **M4-4-MDMP**, which corresponds to 4-methylpentedrone (**4-MPD**) that was reported as NPS in 2014 (EMCDDA–Europol, 2015); 4) Hydroxylation of the propylic chain to afford **M5-4-MDMP**, which has been already reported as a metabolite of **4-MPD** (Gavrilović et al., 2022); 5) Hydroxylation of the *N*-methyl position of **M1-4-MDMP**, yielding **M6-4-MDMP**; 6) *N*-demethylation of **M4-4-MDMP** (or **4-MPD**) to afford **M7-4-MDMP**, which has been reported as a metabolite of **4-MPD** (Gavrilović et al., 2022); and 7) Oxidation of the aromatic hydroxymethyl substituent to afford the carboxylic acid derivative **M8-4-MDMP**. These results show that **4-MDMP** can act as a prodrug of **4-MPD**. The report of a fatal case of intoxication with this cathinone (Cartiser et al., 2021), might constitute an issue of potential toxicity concern associated with **4-MDMP** use. This also implies that those metabolites that are related to **4-MPD** (**M4-MDMP** and **M5-MDMP**) cannot act as specific biomarkers of **4-MDMP** exposure. No Phase II metabolites were identified for this cathinone.

The protonated molecule of **4-MDMP** is observed at *m/z* 220.1698 (+0.9 ppm) and its more significative fragment ions correspond to the oxonium ion at *m/z* 119.0484 (−5.9 ppm) and the loss of the amine moiety at *m/z* 175.1120 (+1.7 ppm) (Supplementary Figure S35). **M1-4-MDMP**, as a product of the keto group reduction, presents a mass increment of 2 u, when compared with the parent cathinone and is observed at *m/z* 222.1859 (+3.1 ppm) (Figure 7). The base peak of the tandem mass spectrum corresponds to the loss of water from the parent protonated molecule at *m/z* 204.1751 (+2.0 ppm) (Supplementary Figure S36). Two ions, eluting at 6.7 and 7.4 min, were assigned to products of the direct hydroxylation of **4-MDMP**, based on the mass increment of 16 u relative to the parent cathinone. The protonated molecules of **M2-4-MDMP** and **M3-4-MDMP** were observed at *m/z* 236.1658 (+5.5 ppm) and 236.1650 (+2.1 ppm), respectively. These metabolites were assigned to the benzyl and alkyl hydroxylated products, respectively, based on the observation of key diagnostic fragment ions in their MS/MS spectra. In fact, the tandem mass spectrum of **M2-4-MDMP** presents at *m/z* 135.0447 (+4.4 ppm) the fragment ion (Supplementary Figure S37), corresponding to the aryloxonium ion bearing a hydroxyl substituent, which attests the aromatic moiety as the hydroxylation position. The fact that the benzyl group is the aromatic position more prone to this metabolic transformation has led to our structural proposal. The propyl alkyl group as the hydroxylation position of **M3-4-MDMP** was assign based on the observation of the diagnostic fragment ion at *m/z* 161.0957 (−2.5) (Supplementary Figure S38). This fragment ion stemms from the initial loss of CO, (Pozo et al., 2015), frequently observed for other cathinones, followed by the loss of the amine moiety. The hydroxylation of the aromatic moiety is discarded for this metabolite by the observation of the non-hydroxylated oxonium ion at *m/z* 119.0491 (0.0 ppm). The protonated molecule of **M4-4-MDMP** is observed at *m/z* 206.1549 (+4.8 ppm) and, as mentioned before, it corresponds to the protonated molecule of the previously reported **4-MPD**. The fact that we obtained a very similar fragmentation pattern (Supplementary Figure S39) to the one previously described for **4-MPD** (Apirakkan et al., 2018) further substantiates this assignment. Two signals at *m/z* 222.1499 that eluted at different retention times but shared very similar fragmentation pattern were assigned to **M5-4-MDMP**: hydroxylated metabolites of **4-MPD**. While an exact match with the tandem mass spectrum reported for the product of benzyl hydroxilation was observed (Gavrilović, et al., 2022), we believe that one of these ions might correspond to the product of hydroxylation at the ethyl alkyl moiety. As shown in Supplementary Figure S40, the fragmentation pattern observed is also compatible with this possibility.

Two other ions were detected at *m/z* 238.1810 and eluting at 3.5 and 6.6 min, **M6-4-MDMPa** and **M6-4-MDMPb**, respectivelly (Supplementary Figure S41A). Compatible with the hydroxylation of **M1-4-MDMP**, these two metabolites were assigned to two isomeric products of the hydroxylation of the aromatic moiety. The observation of the fragment ion at *m/z* 110.1125 (+4.0 ppm), corresponding to the *N*-but-1-en-*N*,*N*-dimethyl-amine ion discards the alkyl moieties as possible hydroxylation positions.

The protonated molecule of **M7-4-MDMP** is observed at *m/z* 192.1383 and presents a fragmentation pattern (Supplementary Figure S42) identical to the one described for the *N*-demethylation product of **4-MPD** (Apirakkan et al., 2018). The protonated molecule of **M8-4-MDMP** is observed at *m/z* 250.1449 (+4.4 ppm) and the observation of the fragment ion at *m/z* 149.0245 (+8.0 ppm) compatible with aryloxonium ion bearing a carboxylic acid substituent, substantiates our assignment (Supplementary Figure S43).

#### 4-MNEB

Two Phase I metabolites were identified for **4-MNEB**: **M1-4-MNEB**, stemming from reduction of the keto moiety of the parent cathinone, and **M2-4-MNEB**, afforded upon hydroxylation of the alkyl substituent (Figure 8). No Phase II metabolites were identified in incubation run in the presence of Phase I and Phase II co-factors. For the structural assignment of these Phase I metabolites, it was key to understand the mechanisms of fragmentation of the previously undescribed parent cathinone. The protonated molecule of **4-MNEB** is observed at *m/z* 206.1537 (−1.0 ppm) and the base peak of its tandem mass spectrum arises upon loss of water at *m/z* 188.1432 (−1.1 ppm) (Supplementary Figure S45). Two main fragment ions are subsequently obtained following subsequent loss of ethyl and methyl radicals from the base peak fragment ion, at *m/z* 159.10 44 (+0.6 ppm) and 144.0808 (0.0 ppm), respectively. **M1-4-MNEB** is observed at *m/z* 208.1699 (+1.4 ppm), presenting a 2 u mass increment when compared with the protonated molecule of the parent cathinone, compatible with the occurrence of the keto reduction. This metabolic transformation is further corroborated by the observation of the tandem base peak at *m/z* 190.1599 (+4.7 ppm), which presents the same 2 u mass increment when compared with the tandem base peak of the parent cathinone (Supplementary Figure S46). Two ions were observed at *m/z* 222.1495 (eluting at 3.1 and 6.2 min), which present a mass increment of 16 u when compared with the parent cathine, compatible with the formation of a hydroxylated metabolite (Supplementary Figure S47). We suggest that these two metabolites correspond to products of hydroxylation at the benzyl and alkyl positions. The fragmentation pattern observed is compatible with these two possible structures.

#### 4-MDMB

Five distinct Phase I metabolites were identified in **4-MDMP** HLM incubations (Figure 9): 1) **M1-4-MDMB**, the product of keto group reduction; 2) **M2-4-MDMB**, stemming from *N*-demethylation from the parent cathinone; 3) **M3-4-MDMB**, yielded upon hydroxylation of **M2-4-MDMB**; 4) **M5-4-MDMB**, the product of direct hydroxylation of the parent cathinone, at the alkyl position; and 5) **M4-MDMB** arising from the alkyl hydroxylation of the reduced metabolite, **M1-4-MDMB**, and/or reduction of **M5-4-MDMB**. No Phase II metabolite was detected for this cathinone.

The protonated molecule of **4-MDMB** is observed at *m/z* 206.1537 (−1.0 ppm). Similarly to what it was observed for the other *N*,*N*-dimethyl cathinone included in this study, **4-MDMP**, its tandem mass spectrum (Supplementary Figure S49) is governed by the loss of the alkyl and/or amine moieties from the protonated molecule. In fact, the base peak is observed at *m/z* 86.0961 (−3.5 ppm), which corresponds to *N*,*N*-dimethylprop-1-en-1-amonium, and the fragmentation of ion at *m/z* 161.095 (−1.2 ppm), obtained following loss of the amine moiety from the protonated molecule, explains the other main fragment ions: at *m/z* 133.1014, upon CO loss, and *m/z* 105.0689 (−9.5 ppm) following the subsequent neutral loss of ethylene. The reduction product **M1-4-MDMB** present a mass increment of 2 u in relation to the parent cathinone and is observed at *m/z* 208.17 (+3.8 ppm). The base peak of its tandem mass spectrum stems from the water loss from the protonated molecule at *m/z* 190.1601 (+5.8 ppm) (Supplementary Figure S50). The subsequent losses of ethyl radical and *N*,*N*-dimethylprop-1-en-1-amine afford the main fragment ions at *m/z* 161.1204 (+3.1 ppm) and 105.0703 (+3.8 ppm), respectively. A very similar tandem mass fragmentation behavior was observed for the ion corresponding to the *N*-demethylated metabolite, **M2-4-MDMB**, at *m/z* 192.1389 (+3.1 ppm). A mass increment of 16 u, *m/z* 208.1331 (−0.5 ppm), when compared with **M2-4-MDMB** was observed for **M3-4-MDMB**, which is compatible with the hydroxylation of the *N*-demethylated metabolite. The alkyl ethyl chain is suggested for the location of the hydroxylation based on the observation of fragment ion at *m/z* 177.1146 (−1.1 ppm) which is explained by the intramolecular rearrangement with the concomitant loss of an oxygen and methyl radical. **M4-4-MDMB** and **M5-4-MDMB** present a 16 u mass increment when compared with the reduction metabolite **M1-4-MDMB** and the parent **4-MDMB**, respectively, which suggests their hydroxylation. **M4-4-MDMB** is observed at *m/z* 224.1655 (+4.5 ppm), and the base peak of its tandem mass spectrum stems from water loss at *m/z* 206.1550 (+5.1 ppm). The location of the hydroxyl group at the ethyl chain is only suggested based on the identification of the previously identified hydroxylated metabolites (we cannot exclude hydroxylation at the aromatic moiety). Non-etheless the *N*-methyl substituent cannot be discarded as the location of this metabolic transformation, based on the fragmentation pattern observed for this metabolite. Similarly, for **M5-4-MDMB**, observed at *m/z* 222.1493 (+1.8 ppm), whose tandem base peak at *m/z* 119.0492 (+0.8 ppm), the aryl oxonium ion, which does not bear an hydroxyl substituent, the only hydroxylation location that can be discarded is the aromatic moiety.

## Conclusion

The metabolic stability and the metabolite profile of the synthetic cathinones **4-CMC**, **3-CMC**, **4-CIC**, **4-MEAP**, **3-CIC**, **4-MDMB**, **4-MNEB** and **4-MDMP** were determined in HLM incubations, by LC-HRMS/MS. Based on the t_1/2_ values obtained, **4-CMC** and **4-MDMB** were classified as low and high clearance compounds, respectively. All the remaining cathinones included in this study are intermediate clearance compounds. A total of 34 Phase I metabolites were identified for the 8 cathinones selected for this study. Hydroxylation of alkyl and benzyl positions, reduction of β-keto group and *N*-dealkylation constituted the main metabolic transformations identified for all cathinones included in this study. Non-etheless, the structural features of the parent cathinone revealed to have a key role determining the main metabolic products. In fact, for the *N*-ethyl cathinones, **4-MEAP** and **4-MNEB**, the main metabolic pathway was the hydroxylation of the parent substance at the alkyl position, while for the *N*,*N*-dimethyl cathinone **4-MDMB**, the demethylation followed by hydroxylation of the alkyl chain constituted the main metabolic transformation. Reduction of the β-keto group constituted the main metabolic transformation of the chlorinated cathinones **3-CIC** and **4-CIC**. However, for **3-CMC** and **4-CMC**, the hydroxylation of alkyl chain and *N*-dealkylation were suggested as equally important metabolic reactions as the reduction of the β-keto group. Only two glucuronide metabolites were detected in this study, stemming from glucuronic acid conjugation of the reduced metabolites of **4-CIC** and **3-CIC**.

In summary, these results can help to update routine screening methods to attest the consumption of cathinones selected for this study, in forensic and clinical contexts.

## Data Availability

The original contributions presented in the study are included in the article/Supplementary Materials, further inquiries can be directed to the corresponding authors.

## Abbreviations

3-CIC, 3′-chloro-*N*-isopropylcathinone; 4-CIC, 4′-chloro-*N*-isopropylcathinone; 3-CMC, 3′-chloromethcathinone; 4-CMC, 4′-chloromethcathinone; ESI, electrospray ionization; HLM, human liver microsomes; LC-HRMS/MS, liquid chromatography coupled to tandem high-resolution mass spectrometry; 4-MEAP, 4′-methyl-*N*-ethylnorpentedrone; 4-MNEB, 4′-methyl-*N*-ethylbufedrone; 4-MDMP, 4′-methyl-*N*,*N*-dimethylpentedrone; 4-MDMB, 4′-methyl-*N*,*N*-dimethylbufedrone; 4-MPD, 4′-methylpendredrone; MS/MS, tandem mass spectrometry; *m/z*, mass-to-charge ratio; NADPH, 5′-phosphate adenosine 2′,5′-bisphosphate in its reduced form; QTOF, hybrid quadrupole time-of-flight; THF, tetrahydrofuran; UDPGA, uridine diphosphate glucuronic acid.
