# Supplementary material for: Metabolic stability and metabolite profiling of emerging synthetic cathinones
Source: Front Pharmacol. 2023 Mar 24;14:1145140. doi: 10.3389/fphar.2023.1145140 (PMC10080127; doi:10.3389/fphar.2023.1145140)
Supplement: Supplementary file 1 [file DataSheet1.PDF]

Supplementary Material

Metabolic stability and metabolite profiling of emerging synthetic cathinones

Rita P. Lopes,[1,2] Raquel A. Ferro,[2] Margarida Milhazes,[1] Margarida Figueira,[1] Maria João Caldeira,[3] Alexandra M. M. Antunes, [1*†] Helena Gaspar [2,4*†]

[1] Centro de Química Estrutural (CQE), Institute of Molecular Sciences, Departamento de Engenharia Química, Instituto Superior Técnico (IST), ULisboa, Avenida Rovisco Pais, 1049-001 Lisboa, Portugal

[2] BioISI – Biosystems & Integrative Sciences, Faculty of Sciences, University of Lisbon, Institute, Campo Grande, C8, 1749-016 Lisboa, Portugal

[3] Laboratório de Polícia Científica da Polícia Judiciária (LPC/PJ), Novo edifício Sede da Polícia Judiciária, Rua Gomes Freire 1169-007 Lisboa, Portugal

[4] MARE - Marine and Environmental Sciences Centre - Polytechnic of Leiria, Peniche, Portugal, Avenida do Porto de Pesca, 2520-630 Peniche, Portugal

Page	Content
4	**Figure S1**: Metabolic pathways identified for **bupropion**
5	**Figure S2:** Tandem mass spectrum obtained for **bupropion** and proposed structures for the main fragment ions observed.
6	**Figure S3:** Tandem mass spectrum obtained for **M1-bupropion** and proposed structures for the main fragment ions observed.
7	**Figure S4:** Tandem mass spectrum obtained for **M2-bupropion** and proposed structures for the main fragment ions observed.
8	**Figure S5:** Tandem mass spectrum obtained for **M3-bupropion** and proposed structures for the main fragment ions observed.
9	**Figure S6:** Tandem mass spectrum obtained for **M4-bupropion** and proposed structures for the main fragment ions observed.
10	**Figure S7: A.** Depletion Plot; and **B.** ln (relative area) *vs* Time (min) obtained for **3-CMC** incubations in HLM.
11	**Figure S8:** Tandem mass spectrum obtained for **3-CMC** and proposed structures for the main fragment ions observed.
12	**Figure S9:** Tandem mass spectrum obtained for **M1-3-CMC** and proposed structures for the main fragment ions observed.
13	**Figure S10:** Tandem mass spectrum obtained for **M2-3-CMC** & **M2-3-ClC** and proposed structures for the main fragment ions observed.
14	**Figure S11:** Tandem mass spectrum obtained for **M3-3-CMC** & **M3-3-ClC** and proposed structures for the main fragment ions observed.
15	**Figure S12:** Tandem mass spectrum obtained for **M4-3-CMC** and proposed structures for the main fragment ions observed
16	**Figure S13: A.** Depletion Plot; and **B.** ln (relative area) *vs* Time (min) obtained for **4-CMC** incubations in HLM.
17	**Figure S14:** Tandem mass spectrum obtained for **4-CMC** and proposed structures for the main fragment ions observed
18	**Figure S15:** Tandem mass spectrum obtained for **M1-4-CMC** and proposed structures for the main fragment ions observed.
19	**Figure S16:** Tandem mass spectrum obtained for **M2-4-CMC & M2-4-ClC** and proposed structures for the main fragment ions observed.
20	**Figure S17:** Tandem mass spectrum obtained for **M3-4-CMC** and proposed structures for the main fragment ions observed.
21	**Figure S18:** Tandem mass spectrum obtained for **M4-4-CMC** and proposed structures for the main fragment ions observed.
22	**Figure S19: A.** Depletion Plot; and **B.** ln (relative area) *vs* Time (min) obtained for **4-ClC** incubations in HLM.
23	**Figure S20:** Tandem mass spectrum obtained for **4-ClC** and proposed structures for the main fragment ions observed.
24	**Figure S21:** Tandem mass spectrum obtained for **M1-4-ClC** and proposed structures for the main fragment ions observed.
25	**Figure S22:** Tandem mass spectrum obtained for **M4-4-ClC** and proposed structures for the main fragment ions observed.
26	**Figure S23: A.** Depletion Plot; and **B.** ln (relative area) *vs* Time (min) obtained for **3-ClC** incubations in HLM.
27	**Figure S24:** Tandem mass spectrum obtained for **3-ClC** and proposed structures for the main fragment ions observed.
28	**Figure S25:** Tandem mass spectrum obtained for **M1-3-ClC** and proposed structures for the main fragment ions observed.
29	**Figure S26:** Tandem mass spectrum obtained for **M4-3-ClC** and proposed structures for the main fragment ions observed.
30	**Figure S27:** Tandem mass spectrum obtained for **M5-3-ClC** and proposed structures for the main fragment ions observed.
31	**Figure S28: A.** Depletion Plot; and **B.** ln (relative area) *vs* Time (min) obtained for **4-MEAP** incubations in HLM in the presence of NADPH.
32	**Figure S29:** Tandem mass spectrum obtained for **4-MEAP** and proposed structures for the main fragment ions observed.

Page	Content
33	**Figure S30:** Tandem mass spectrum obtained for **M1-4-MEAP** and proposed structures for the main fragment ions observed.
34	**Figure S31:** Tandem mass spectrum obtained for **M2-4-MEAP** and proposed structures for the main fragment ions observed.
35	**Figure S32:** Tandem mass spectrum obtained for **M3-4-MEAP** and proposed structures for the main fragment ions observed.
36	**Figure S33:** Tandem mass spectrum obtained for **M4-4-MEAP** and proposed structures for the main fragment ions observed.
37	**Figure S34: A.** Depletion Plot; and **B.** ln (relative area) *vs* Time (min) obtained for **4-MDMP** incubations in HLM.
38	**Figure S35:** Tandem mass spectrum obtained for **4-MDMP** and proposed structures for the main fragment ions observed.
39	**Figure S36:** Tandem mass spectrum obtained for **M1-4-MDMP** and proposed structures for the main fragment ions observed.
40	**Figure S37:** Tandem mass spectrum obtained for **M2-4-MDMP** and proposed structures for the main fragment ions observed.
41	**Figure S38:** Tandem mass spectrum obtained for **M3-4-MDMP** and proposed structures for the main fragment ions observed.
42	**Figure S39:** Tandem mass spectrum obtained for **M4-4-MDMP** and proposed structures for the main fragment ions observed.
43	**Figure S40: A.** Extracted ion chromatogram; **B.** Tandem mass spectrum obtained for **M5-4-MDMP;** and **C**. Proposed structures for the main fragment ions observed.
44	**Figure S41: A.** Extracted ion chromatogram at *m/z* 238.1810; **B.** Tandem mass spectra obtained for the isobaric ions **M6-4-MDMPa** and **M6-4-MDMPa;** and **C.** proposed structures for the main fragment ions observed.
45	**Figure S42:** Tandem mass spectrum obtained for **M7-4-MDMP** and proposed structures for the main fragment ions observed.
46	**Figure S43:** Tandem mass spectrum obtained for **M8-4-MDMP** and proposed structures for the main fragment ions observed.
47	**Figure S44: A.** Depletion Plot; and **B.** ln (relative area) *vs* Time (min) obtained for **4-MNEB** incubations in HLM.
48	**Figure S45:** Tandem mass spectrum obtained for **4-MNEB** and proposed structures for the main fragment ions observed.
49	**Figure S46:** Tandem mass spectrum obtained for **M1-4-MNEB** and proposed structures for the main fragment ions observed.
50	**Figure S47: A.** Extracted ion chromatogram; **B.** Tandem mass spectrum obtained for **M2-4-MNEB;** and C. proposed structures for the main fragment ions observed.
51	**Figure S48: A.** Depletion Plot; and **B.** ln (relative area) *vs* Time (min) obtained for **4-MDMB** incubations in HLM.
52	**Figure S49:** Tandem mass spectrum obtained for **4-MDMB** and proposed structures for the main fragment ions observed.
53	**Figure S50:** Tandem mass spectrum obtained for **M1-4-MDMB** and proposed structures for the main fragment ions observed.
54	**Figure S51:** Tandem mass spectrum obtained for **M2-4-MDMB** and proposed structures for the main fragment ions observed.
55	**Figure S52:** Tandem mass spectrum obtained for **M3-4-MDMB** and proposed structures for the main fragment ions observed.
56	**Figure S53:** Tandem mass spectrum obtained for **M4-4-MDMB** and proposed structures for the main fragment ions observed.
57	**Figure S54:** Tandem mass spectrum obtained for **M5-4-MDMB** and proposed structures for the main fragment ions observed.
58	**Figure S55:** Relative abundance of Phase I metabolites over HLM incubation time, for the 8 selected cathinones
59	**Table S1**: NMR spectroscopic data of the chloro-cathinones, obtained in DMSO-d6
60	**Figure S56: 3-CMC** ^{1}H NMR and ^{13}C NMR spectra, recorded in DMSO-d6.
61	**Figure S57: 4-CMC** ^{1}H NMR and ^{13}C NMR spectra, recorded in DMSO-d6.
62	**Figure S58: 3-CIC** ^{1}H NMR and ^{13}C NMR spectra, recorded in DMSO-d6.
63	**Figure S59: 4-CIC** ^{1}H NMR and ^{13}C NMR spectra, recorded in DMSO-d6.
64	**Figure S60: 4-MDMB** ^{1}H NMR and ^{13}C NMR spectra, recorded in DMSO-d6.
65	**Figure S61: 4-MDMP** ^{1}H NMR and ^{13}C NMR spectra, recorded in DMSO-d6.
66	**Figure S62: 4-MNEB** ^{1}H NMR and ^{13}C NMR spectra, recorded in DMSO-d6.
67	**Figure S63: 4-MEAP** ^{1}H NMR and ^{13}C NMR spectra, recorded in DMSO-d6.

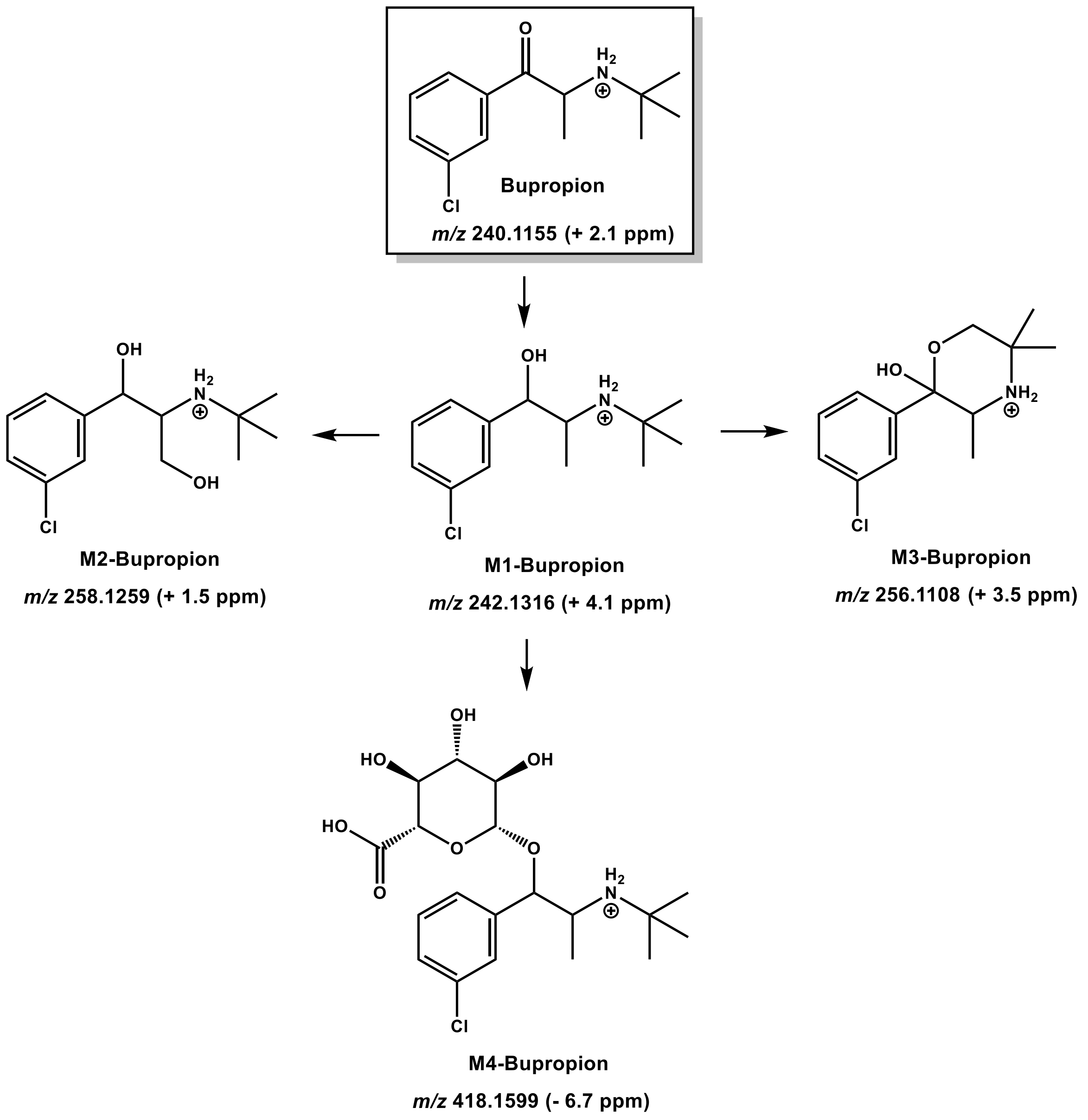

Figure S1: Metabolic pathways identified for Bupropion

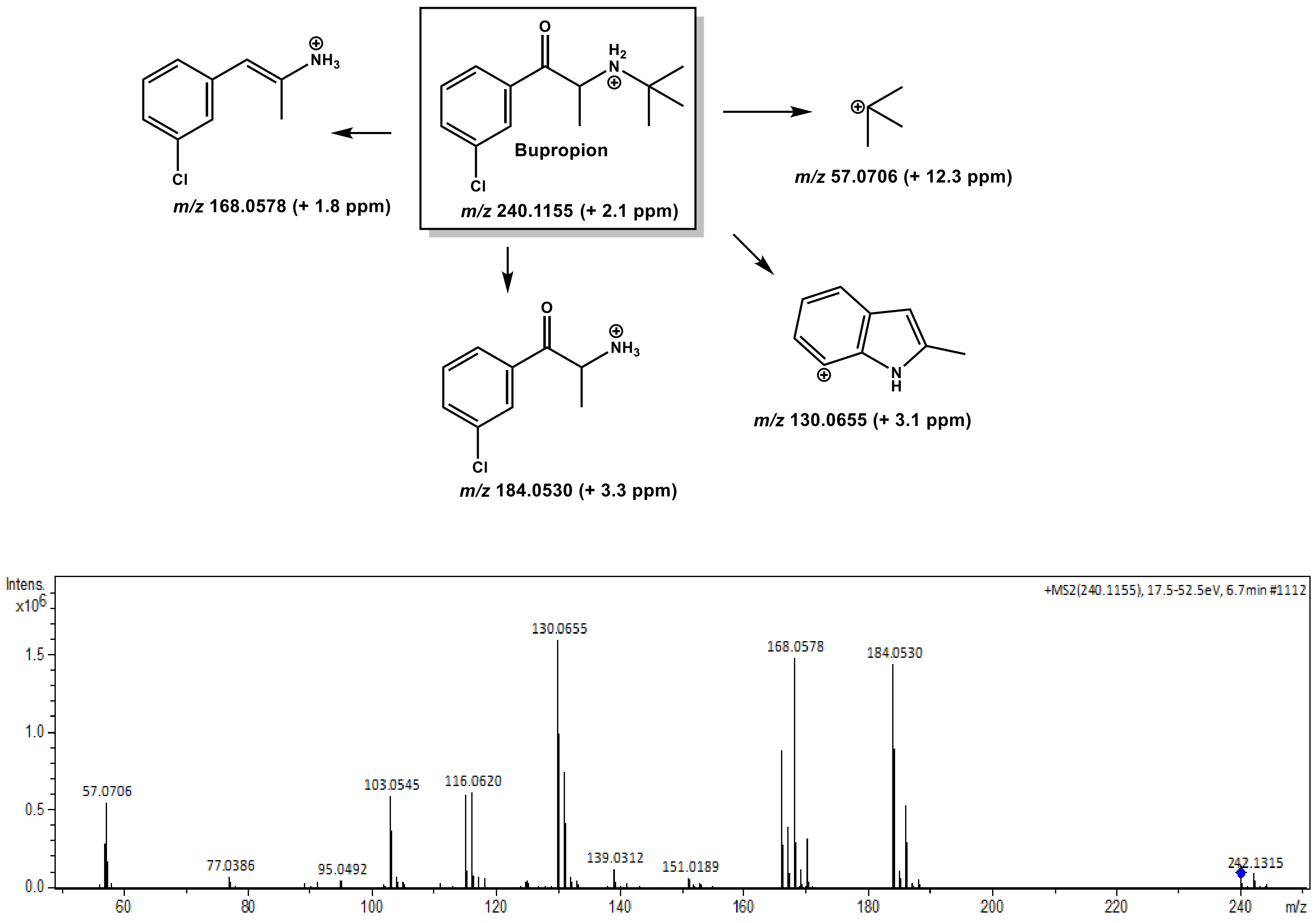

Figure S2: Tandem mass spectrum obtained for **bupropion** and proposed structures for the main fragment ions observed.

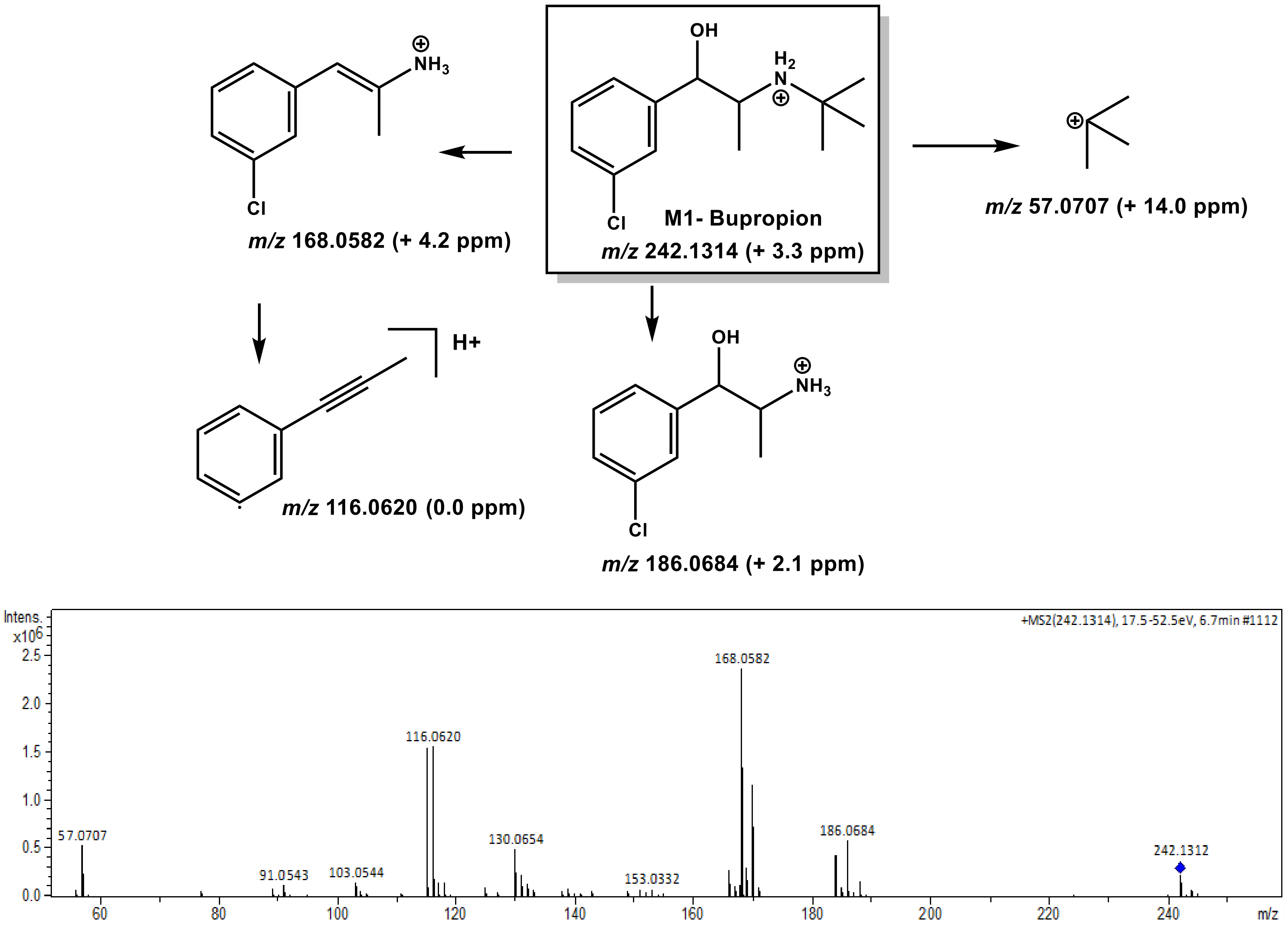

Figure S3: Tandem mass spectrum obtained for **M1-bupropion** and proposed structures for the main fragment ions observed.

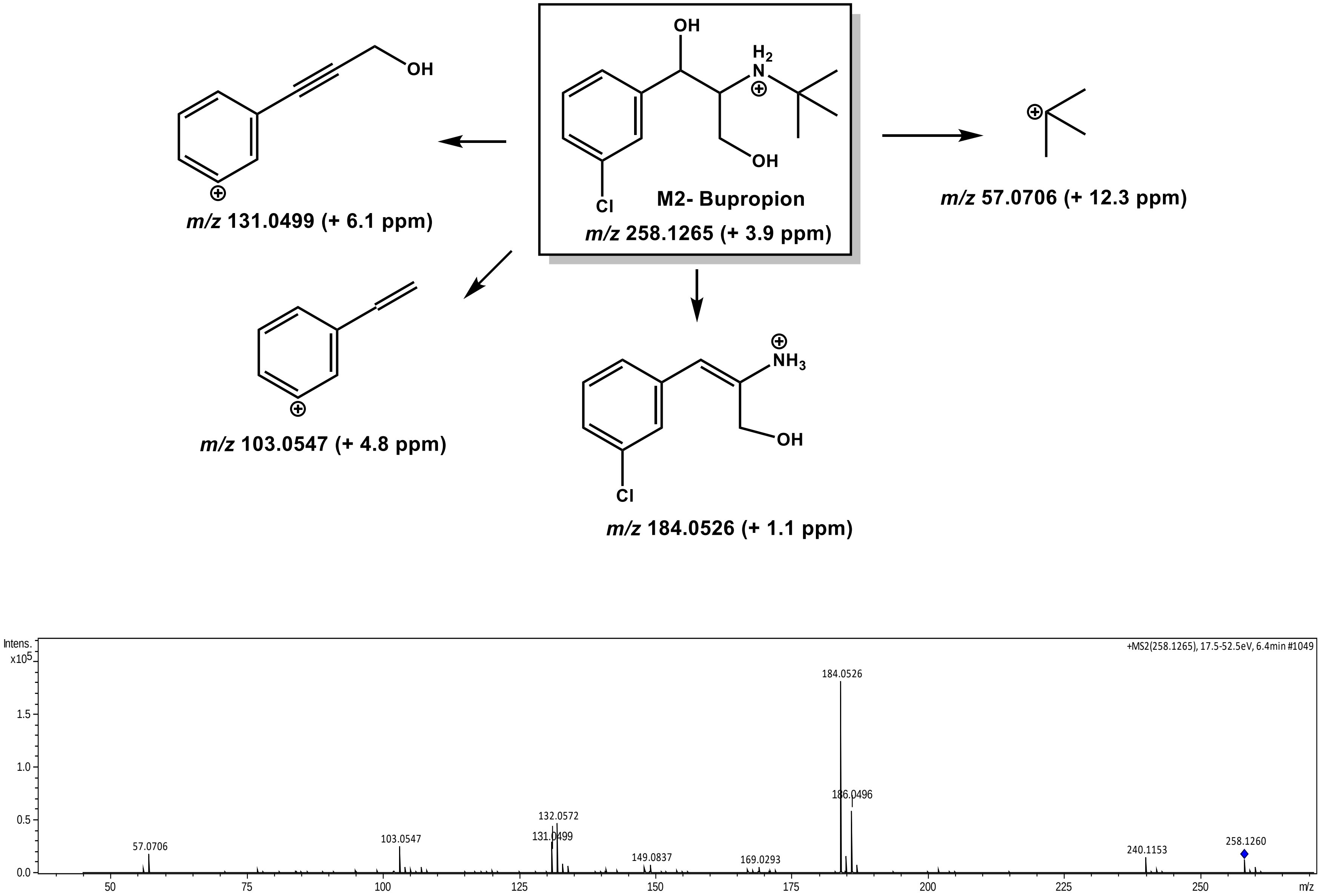

Figure S4: Tandem mass spectrum obtained for **M2-bupropion** and proposed structures for the main fragment ions observed.

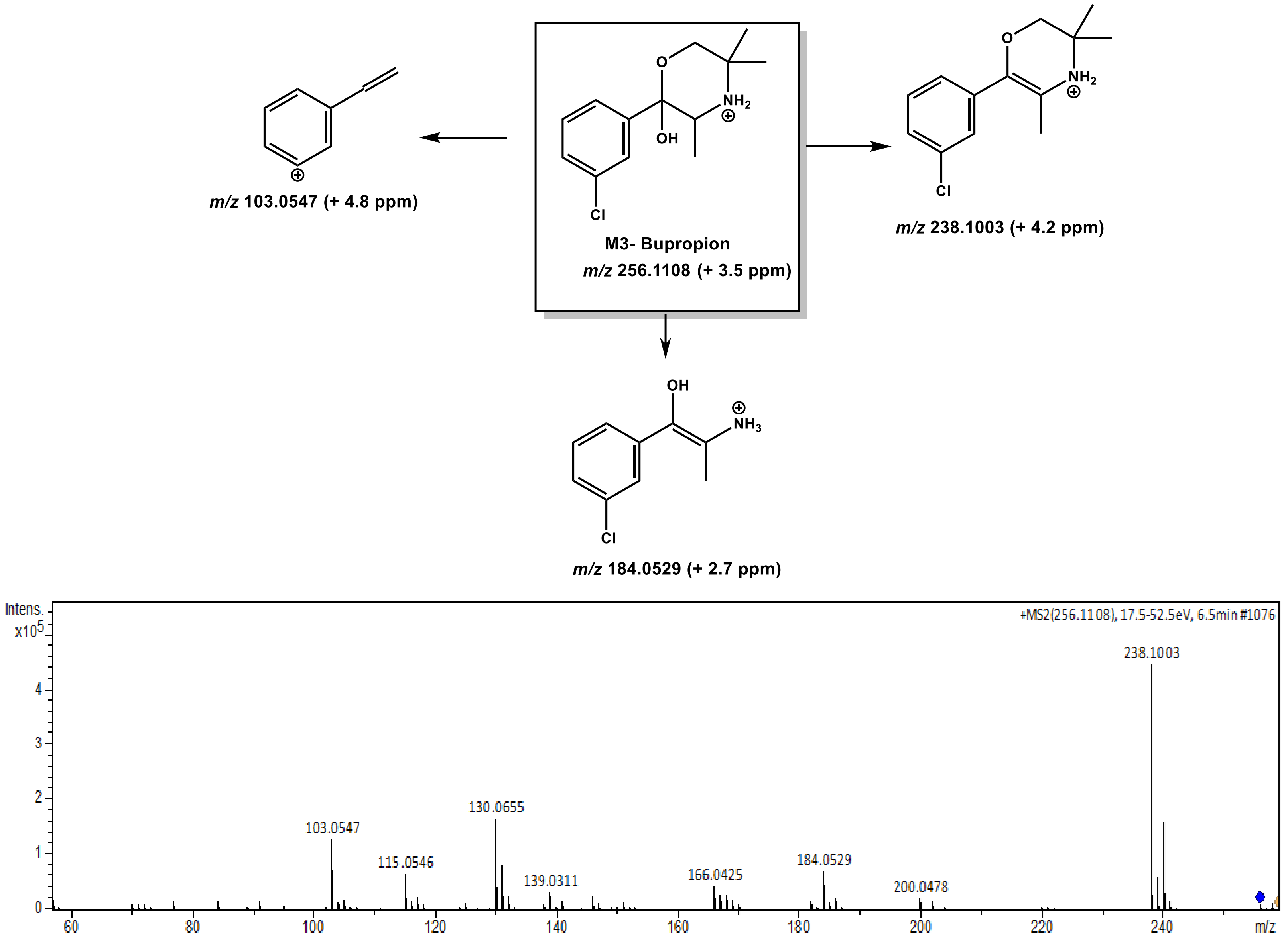

Figure S5: Tandem mass spectrum obtained for **M3-bupropion** and proposed structures for the main fragment ions observed.

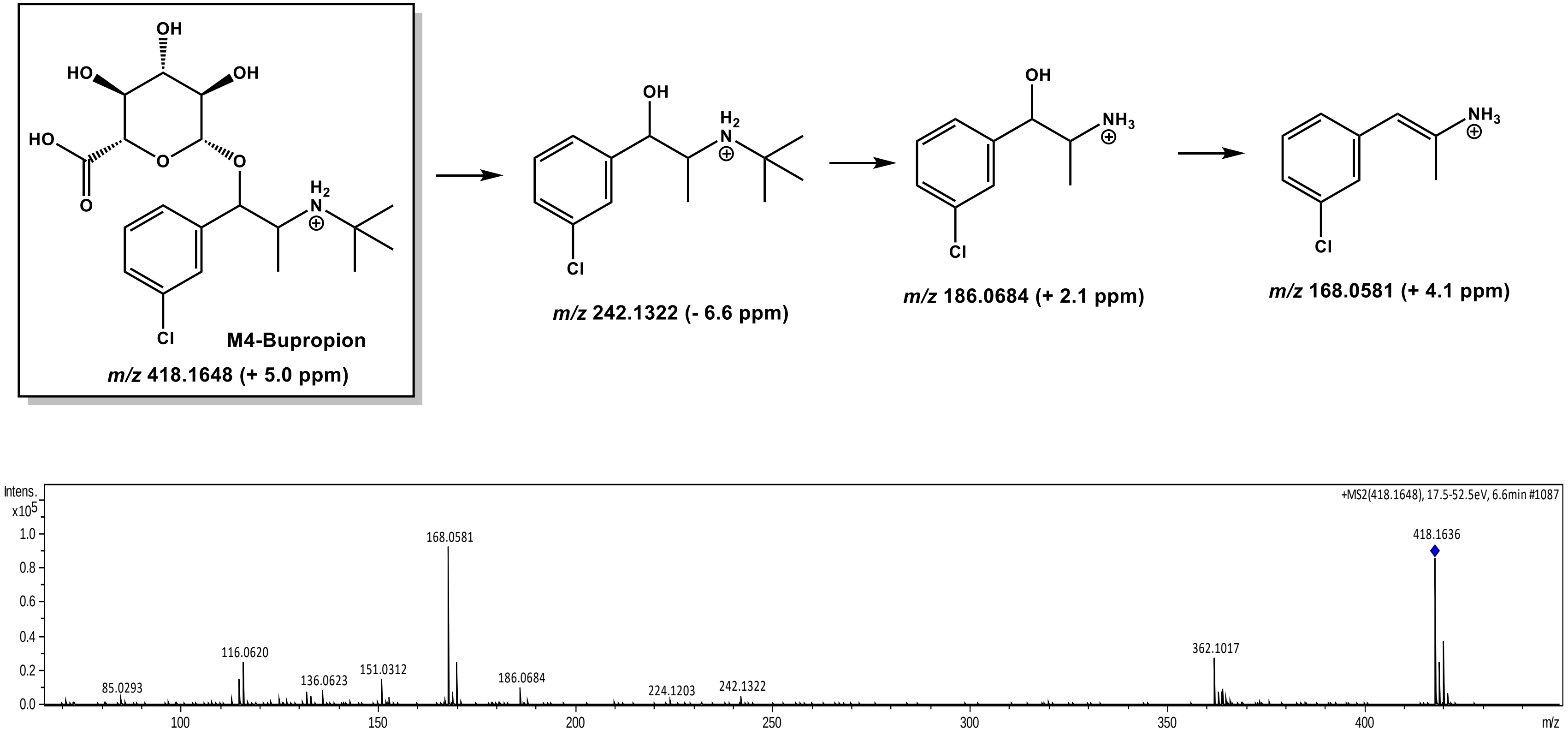

Figure S6: Tandem mass spectrum obtained for **M4-bupropion** and proposed structures for the main fragment ions observed.

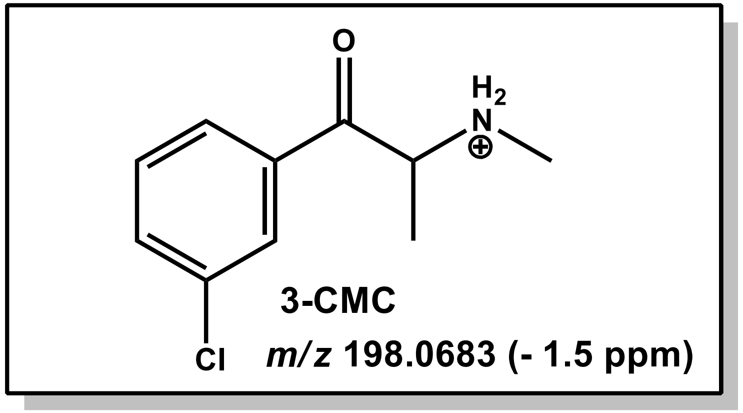

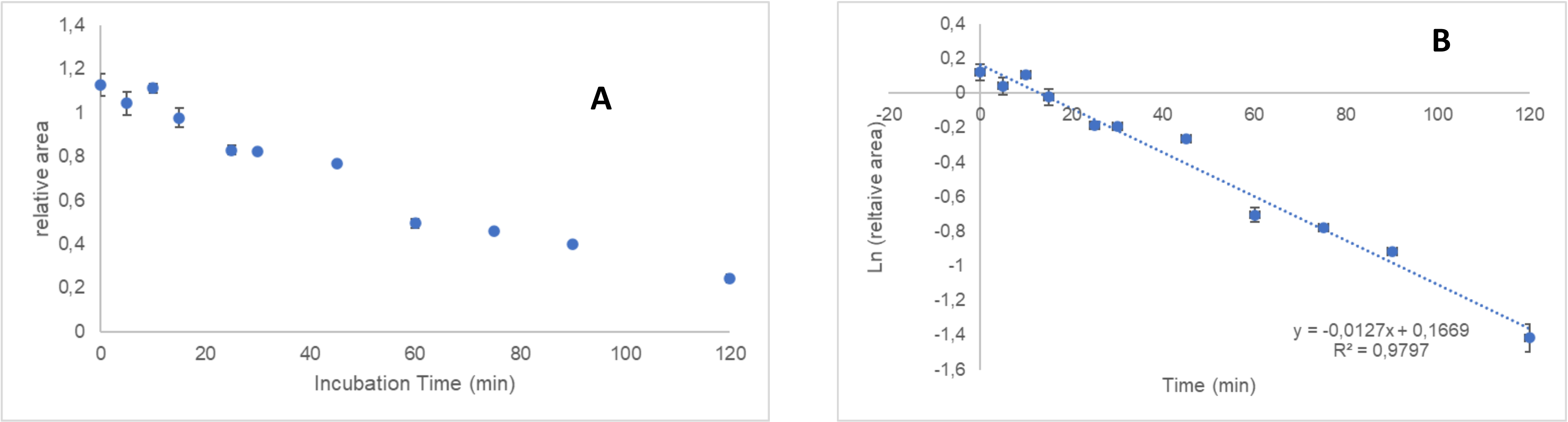

Figure S7: A. Depletion Plot; and **B.** ln (relative area) *vs* Time (min) obtained for **3-CMC** incubations in HLM.

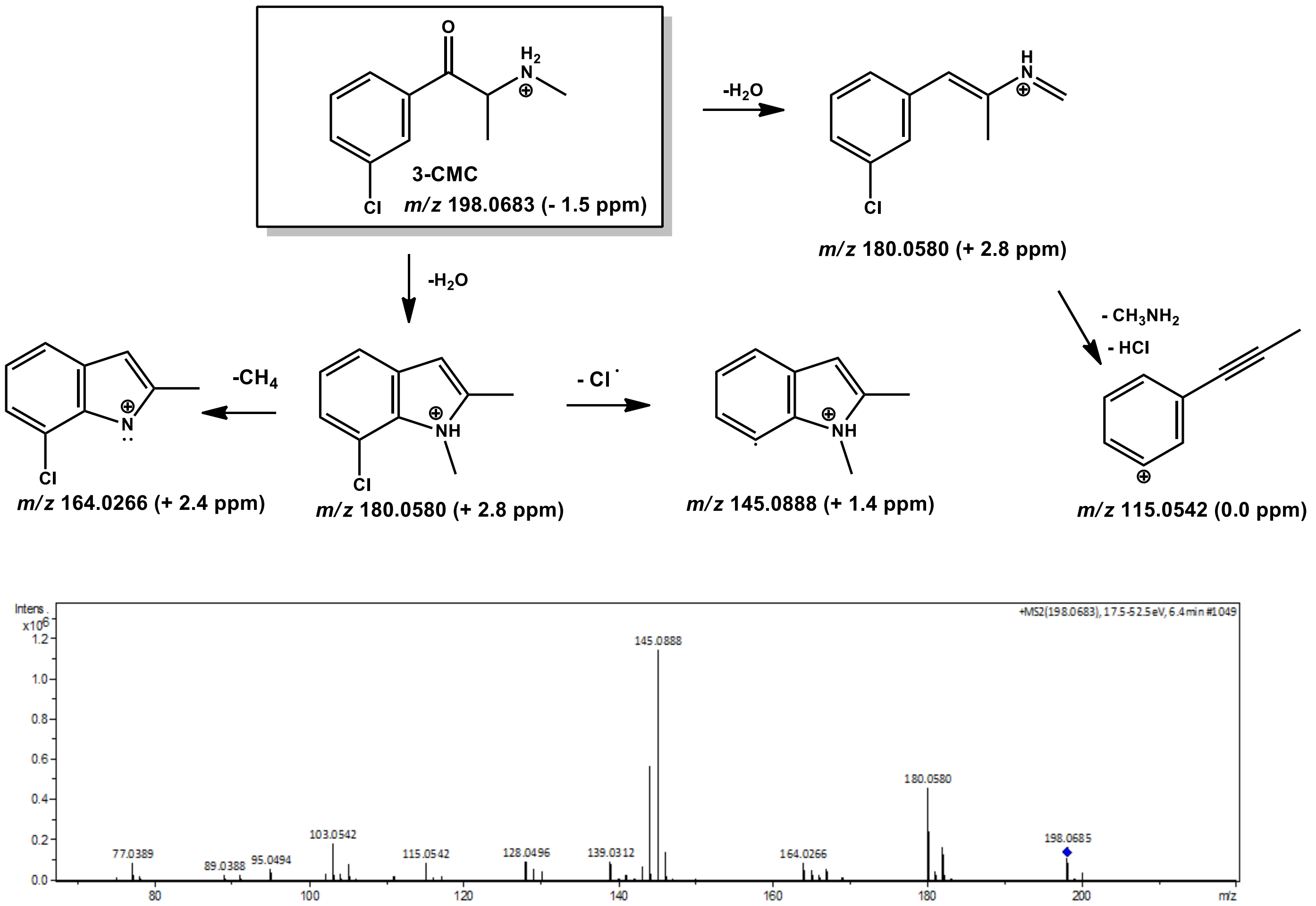

Figure S8: Tandem mass spectrum obtained for **3-CMC** and proposed structures for the main fragment ions observed.

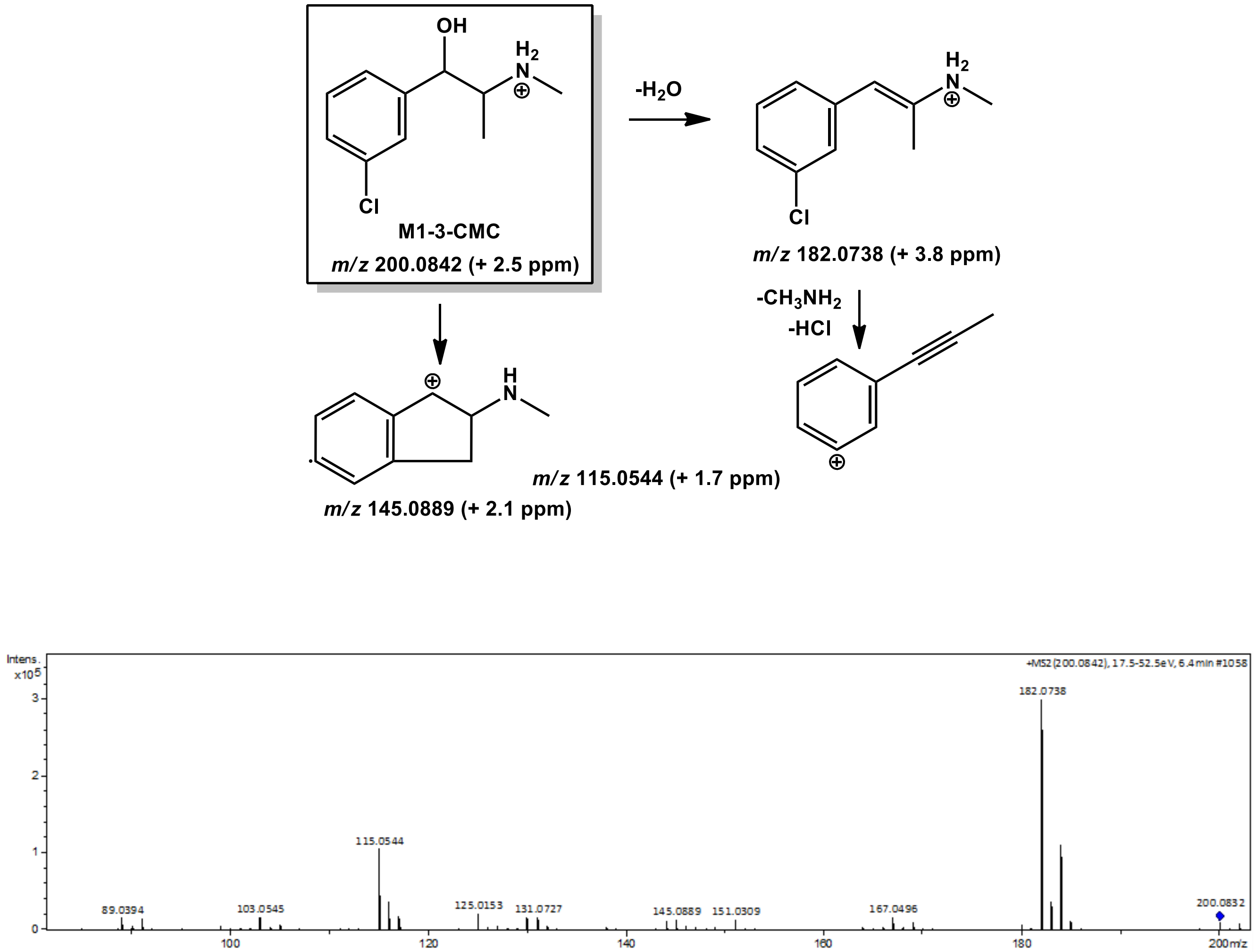

Figure S9: Tandem mass spectrum obtained for **M1-3-CMC** and proposed structures for the main fragment ions observed.

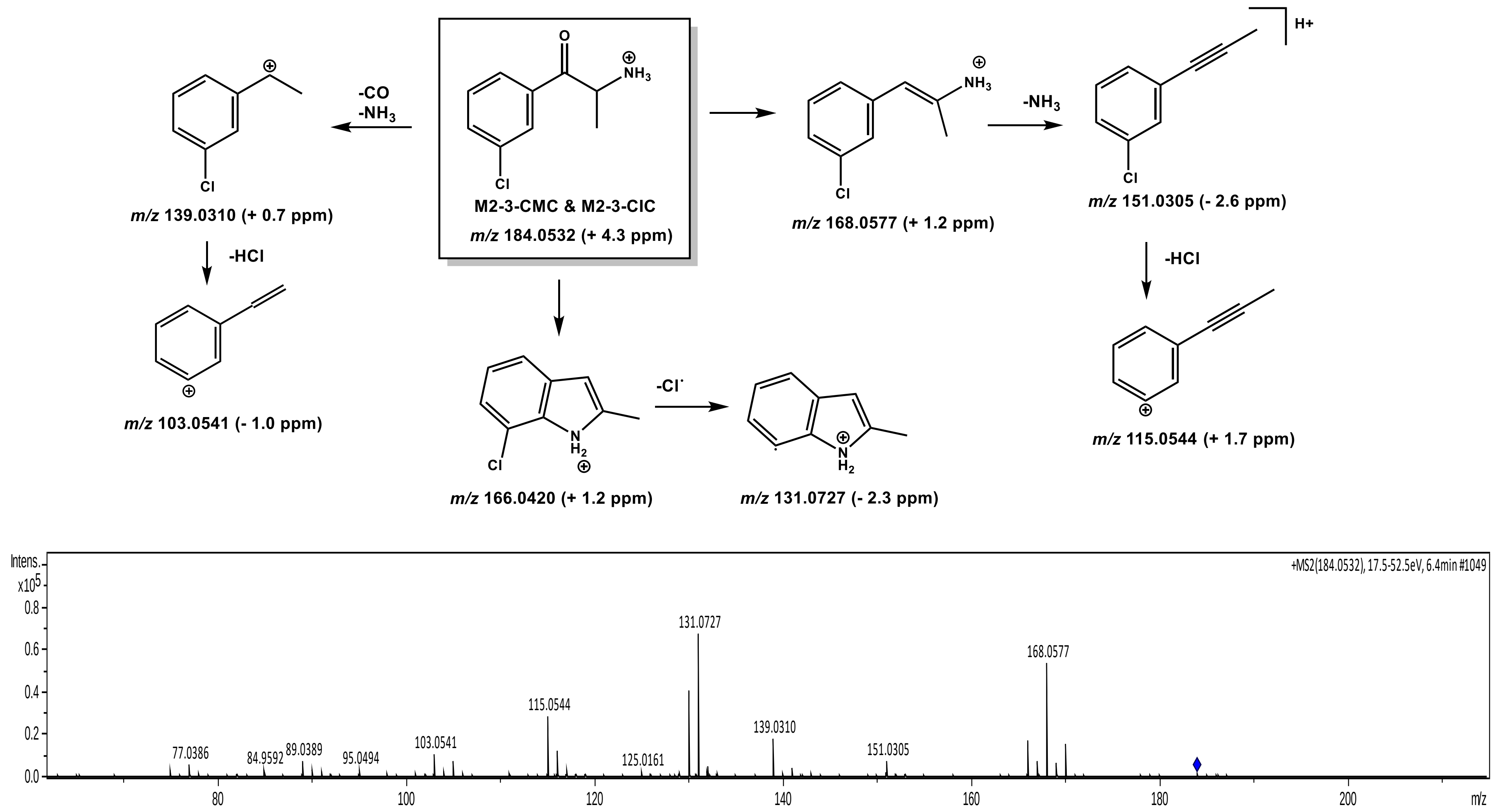

Figure S10: Tandem mass spectrum obtained for **M2-3-CMC** & **M2-3-ClC** and proposed structures for the main fragment ions observed.

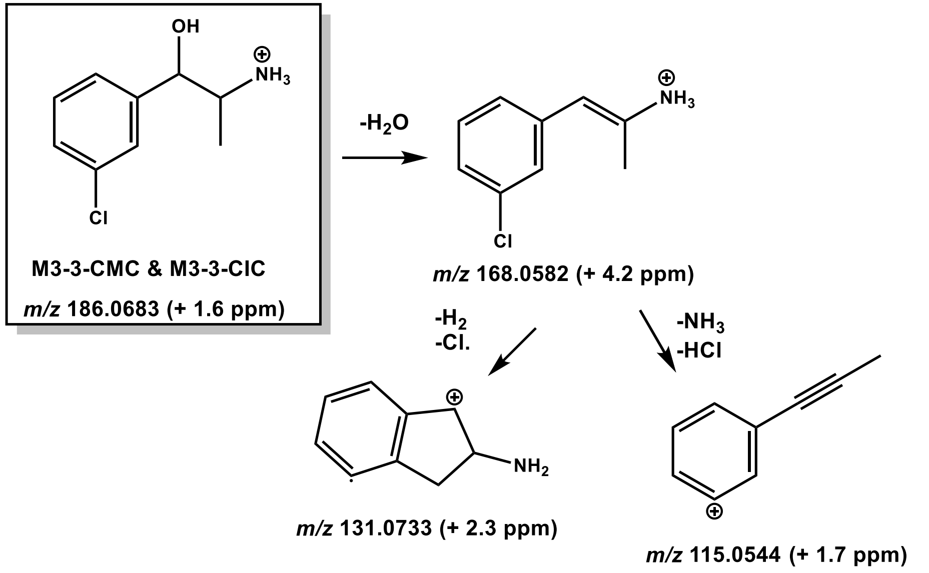

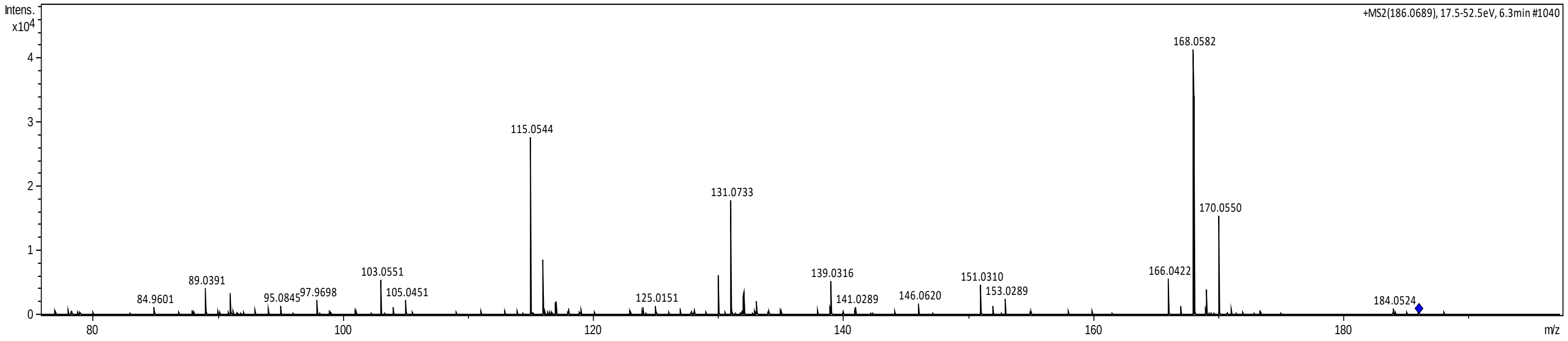

Figure S11: Tandem mass spectrum obtained for **M3-3-CMC** & **M3-3-ClC** and proposed structures for the main fragment ions observed.

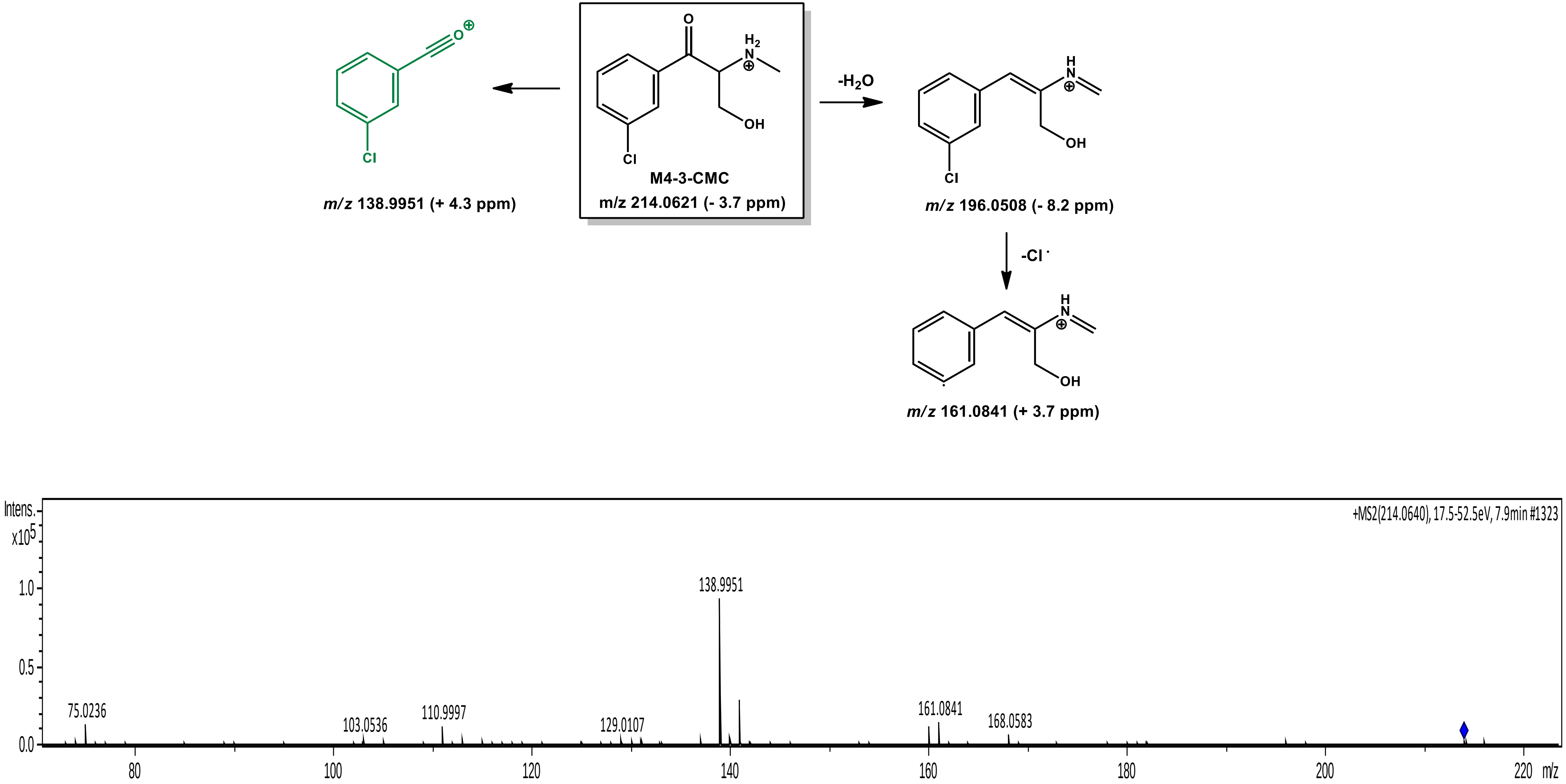

Figure S12: Tandem mass spectrum obtained for **M4-3-CMC** and proposed structures for the main fragment ions observed.

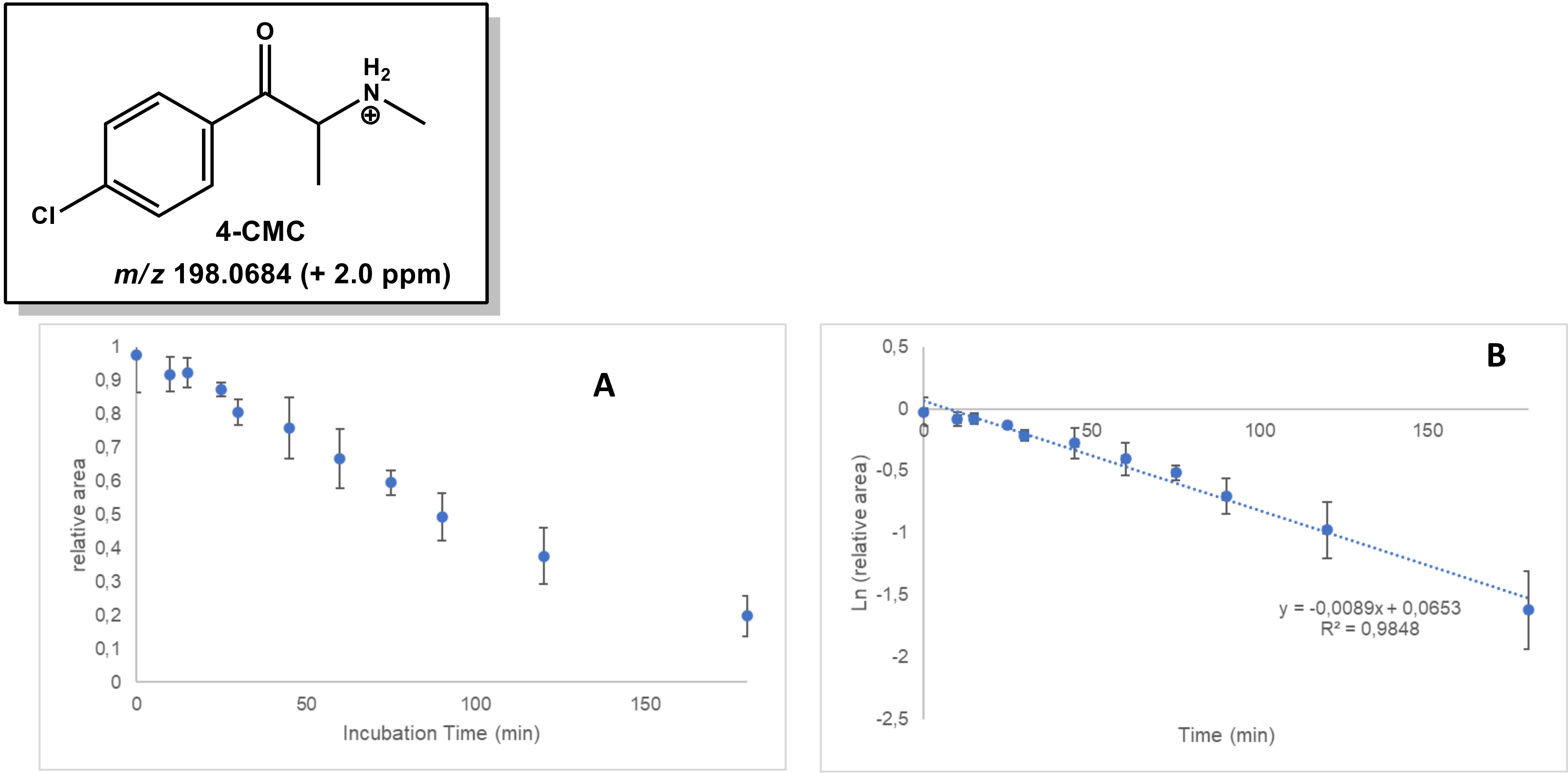

Figure S13: A. Depletion Plot; and **B.** ln (relative area) *vs* Time (min) obtained for **4-CMC** incubations in HLM.

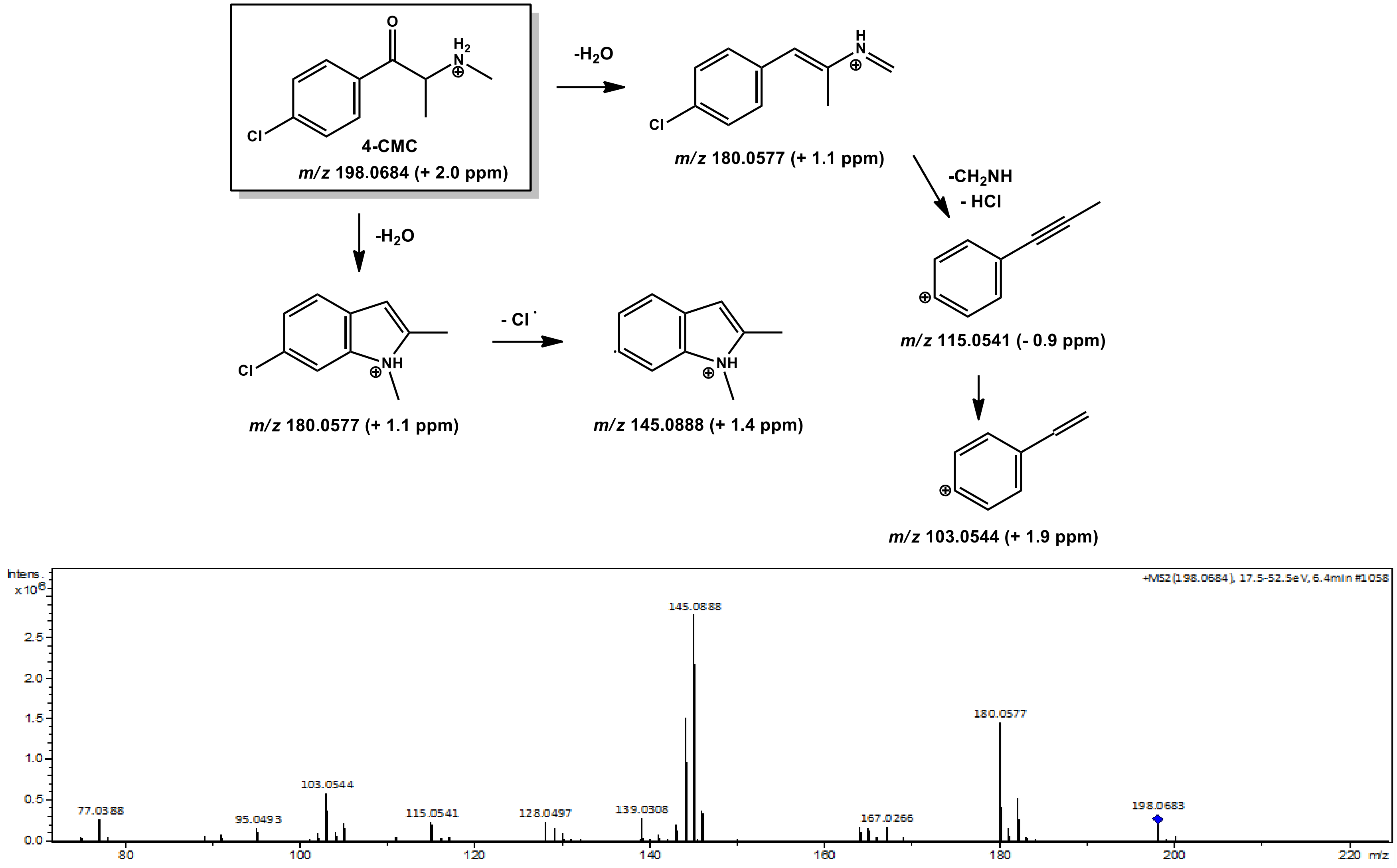

Figure S14: Tandem mass spectrum obtained for **4-CMC** and proposed structures for the main fragment ions observed.

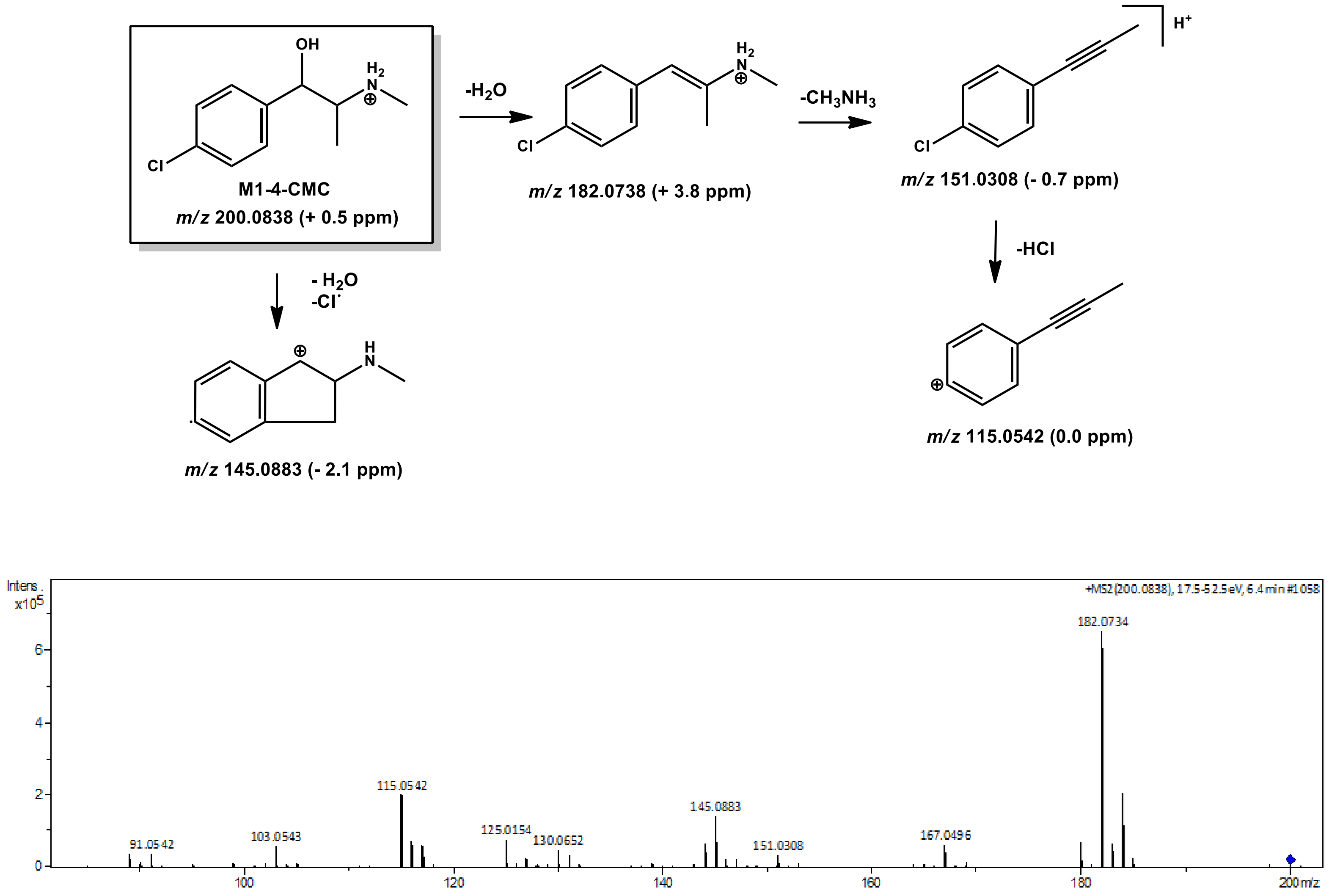

Figure S15: Tandem mass spectrum obtained for **M1-4-CMC** and proposed structures for the main fragment ions observed.

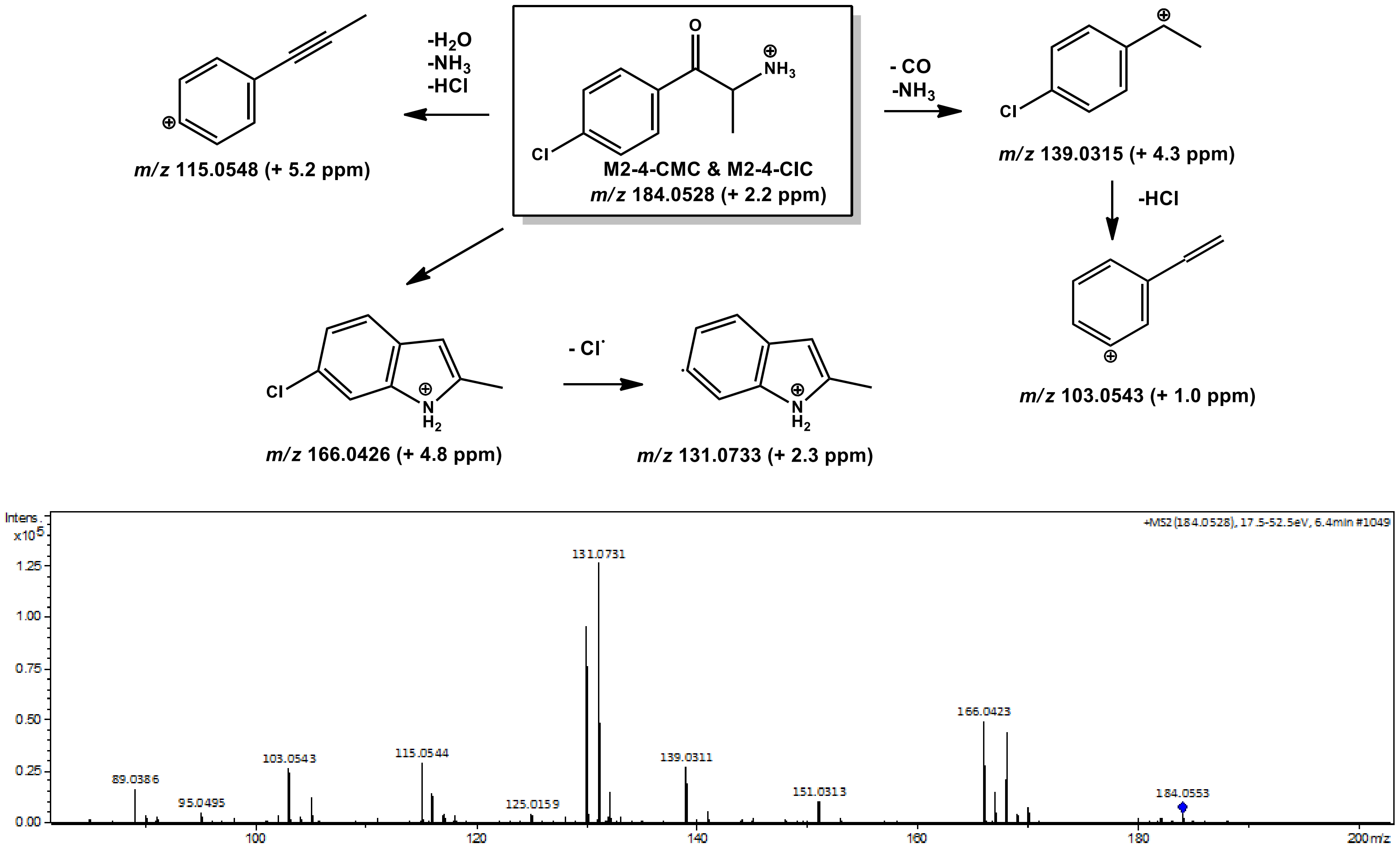

Figure S16: Tandem mass spectrum obtained for **M2-4-CMC & M2-4-ClC** and proposed structures for the main fragment ions observed.

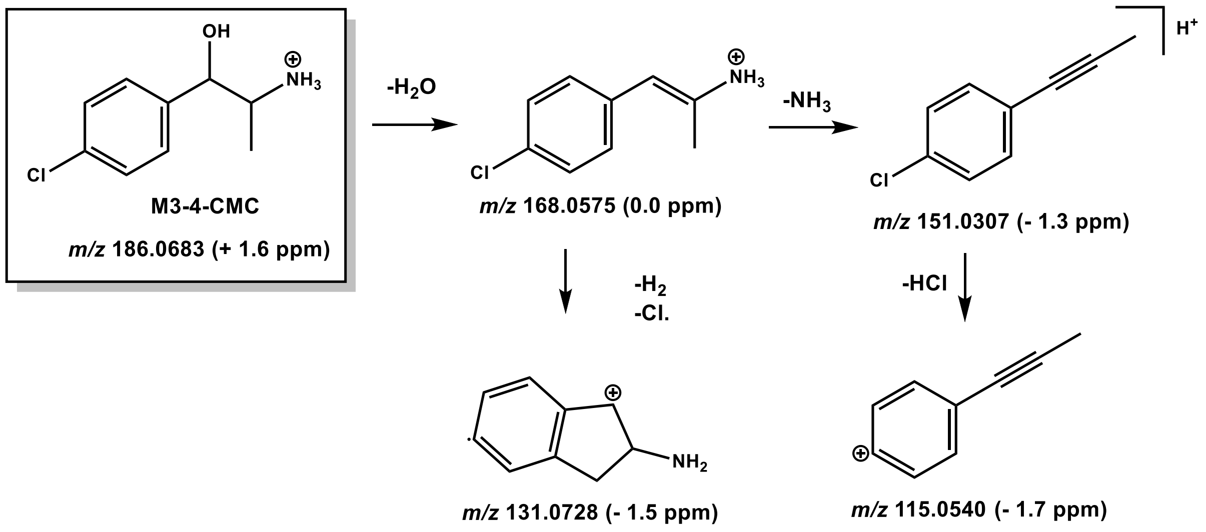

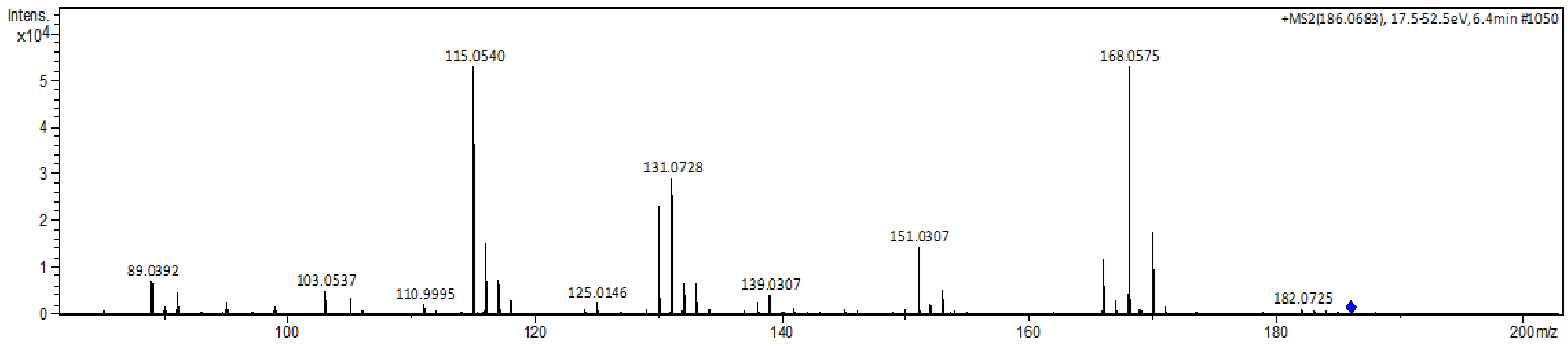

Figure S17: Tandem mass spectrum obtained for **M3-4-CMC** and proposed structures for the main fragment ions observed.

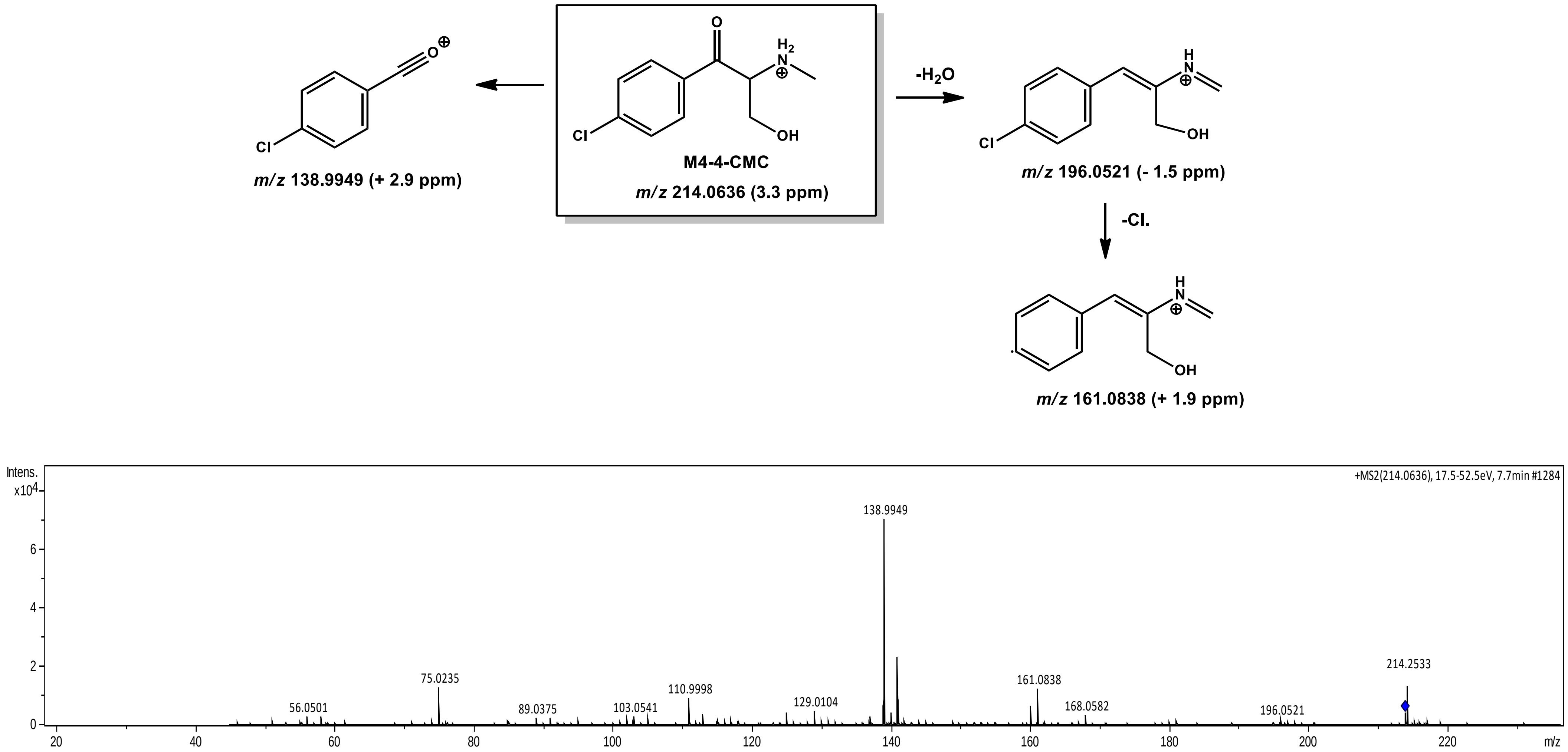

Figure S18: Tandem mass spectrum obtained for **M4-4-CMC** and proposed structures for the main fragment ions observed.

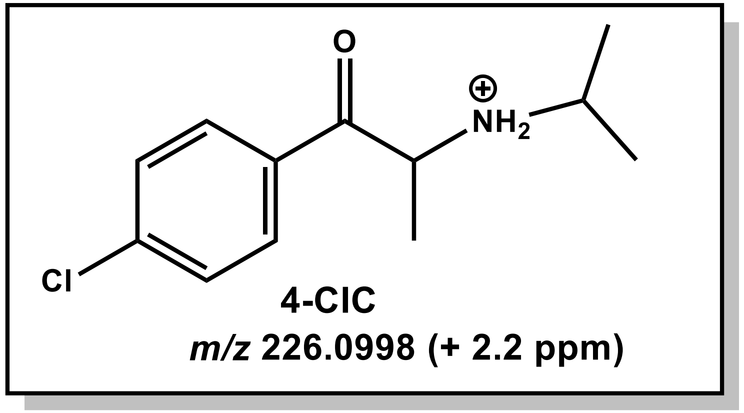

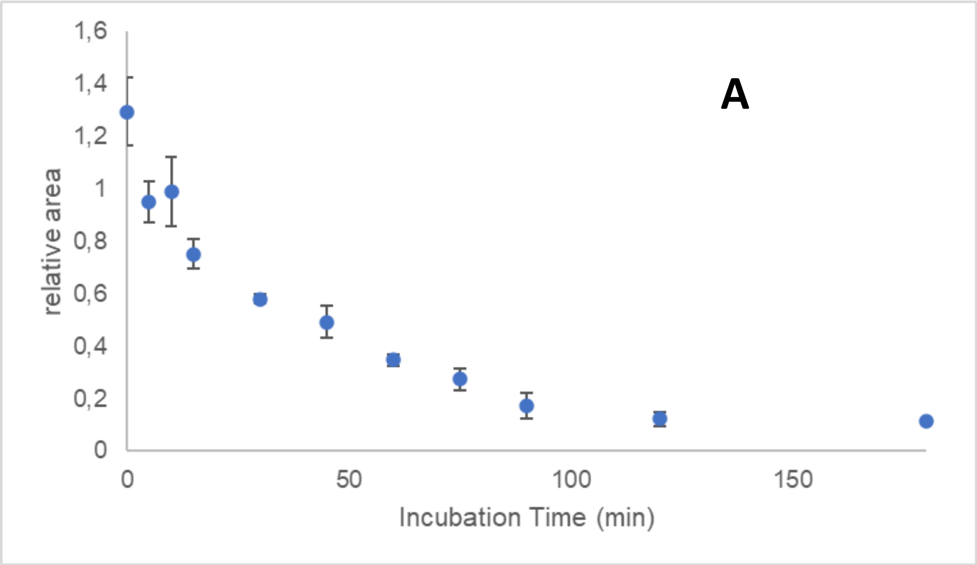

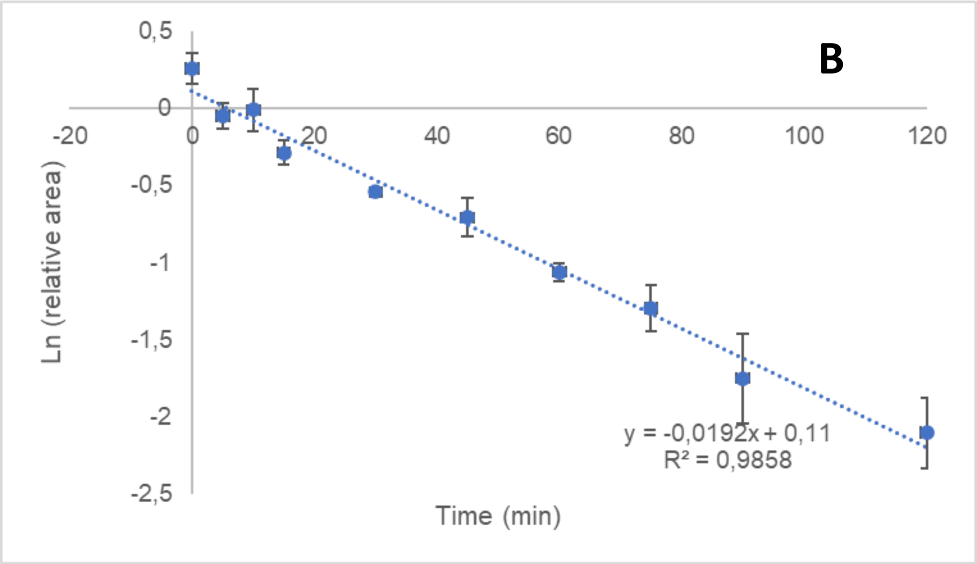

Figure S19: A. Depletion Plot; and **B.** Ln (relative area) *vs* Time (min) obtained for **4-ClC** incubations in HLM.

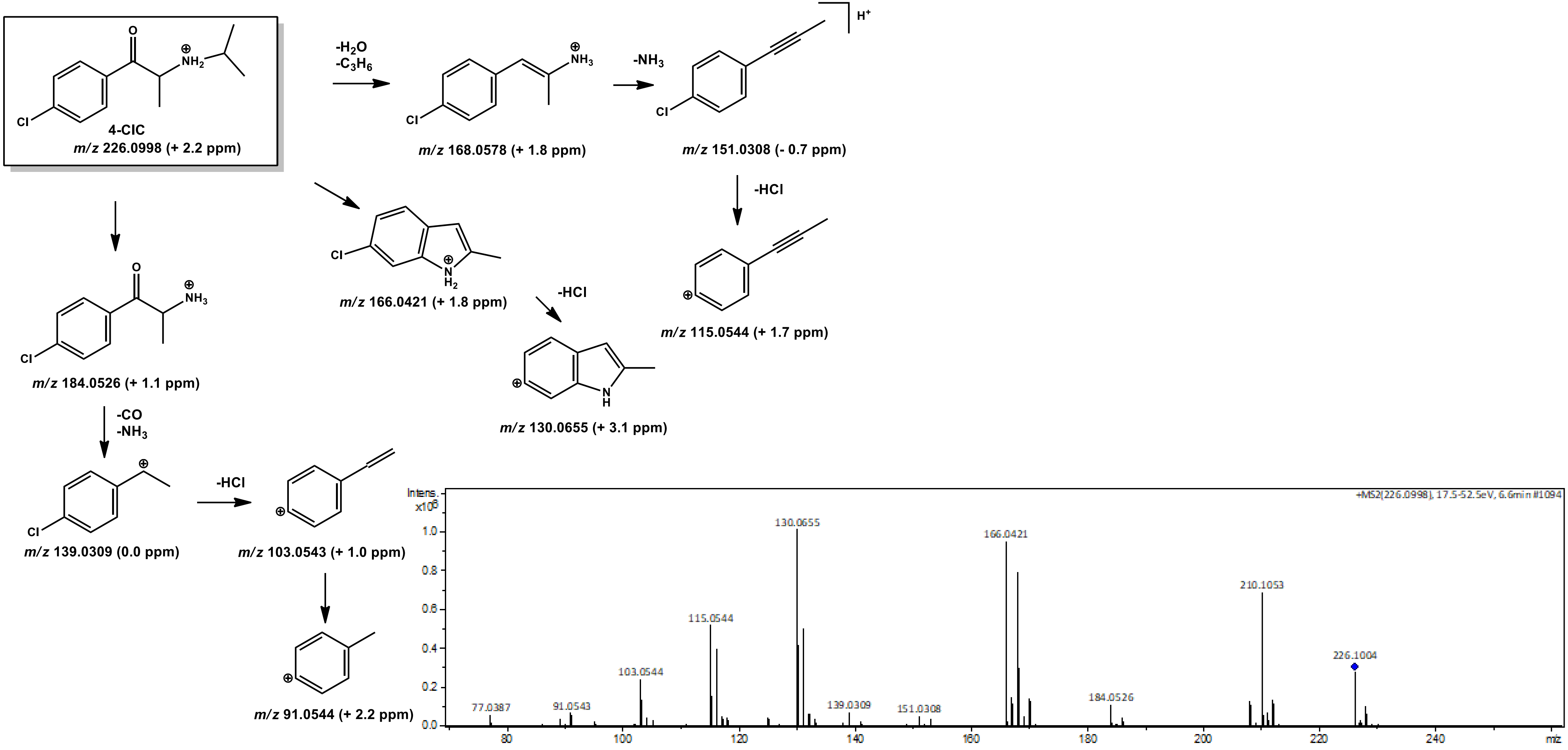

Figure S20: Tandem mass spectrum obtained for **4-ClC** and proposed structures for the main fragment ions observed.

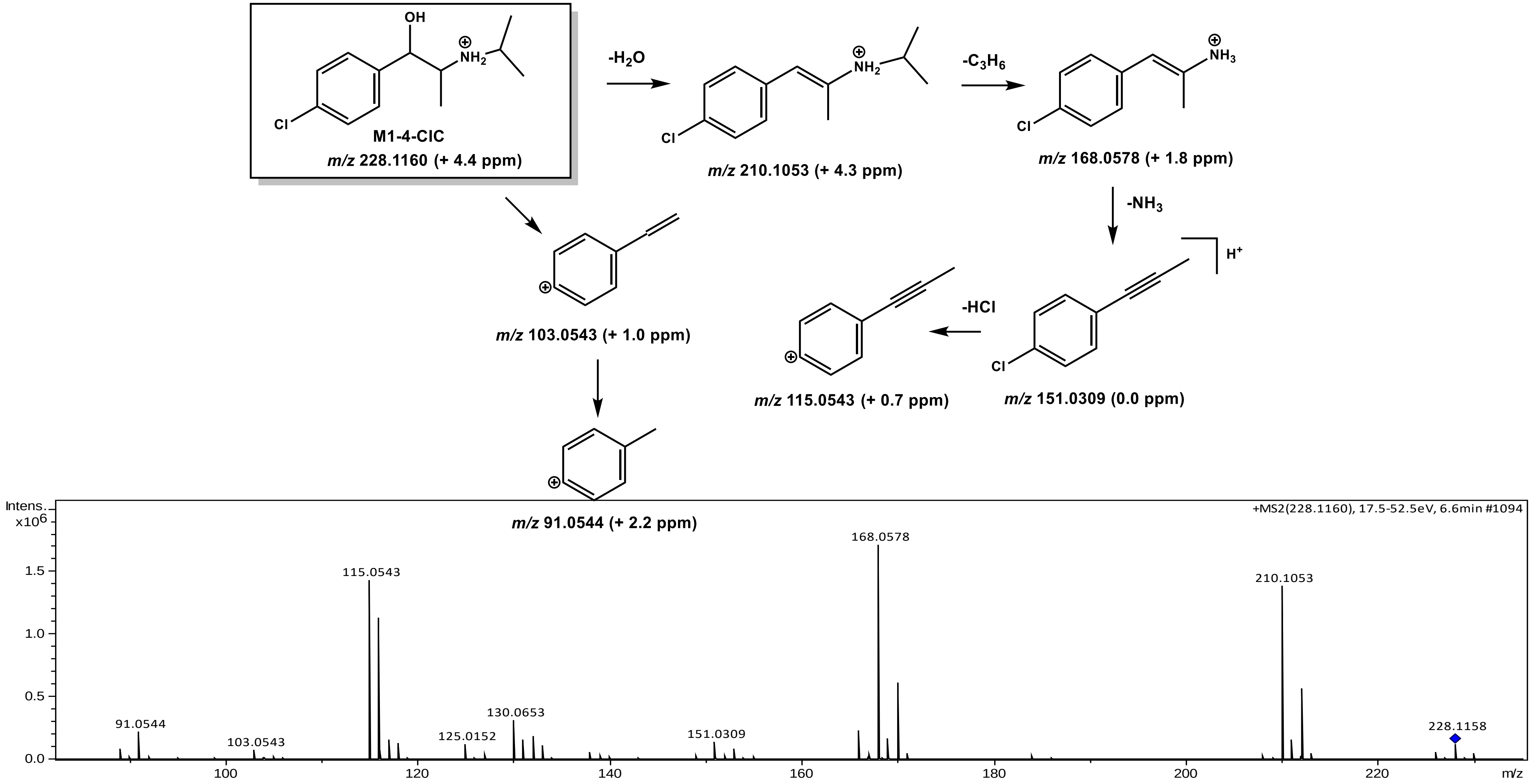

Figure S21: Tandem mass spectrum obtained for **M1-4-ClC** and proposed structures for the main fragment ions observed.

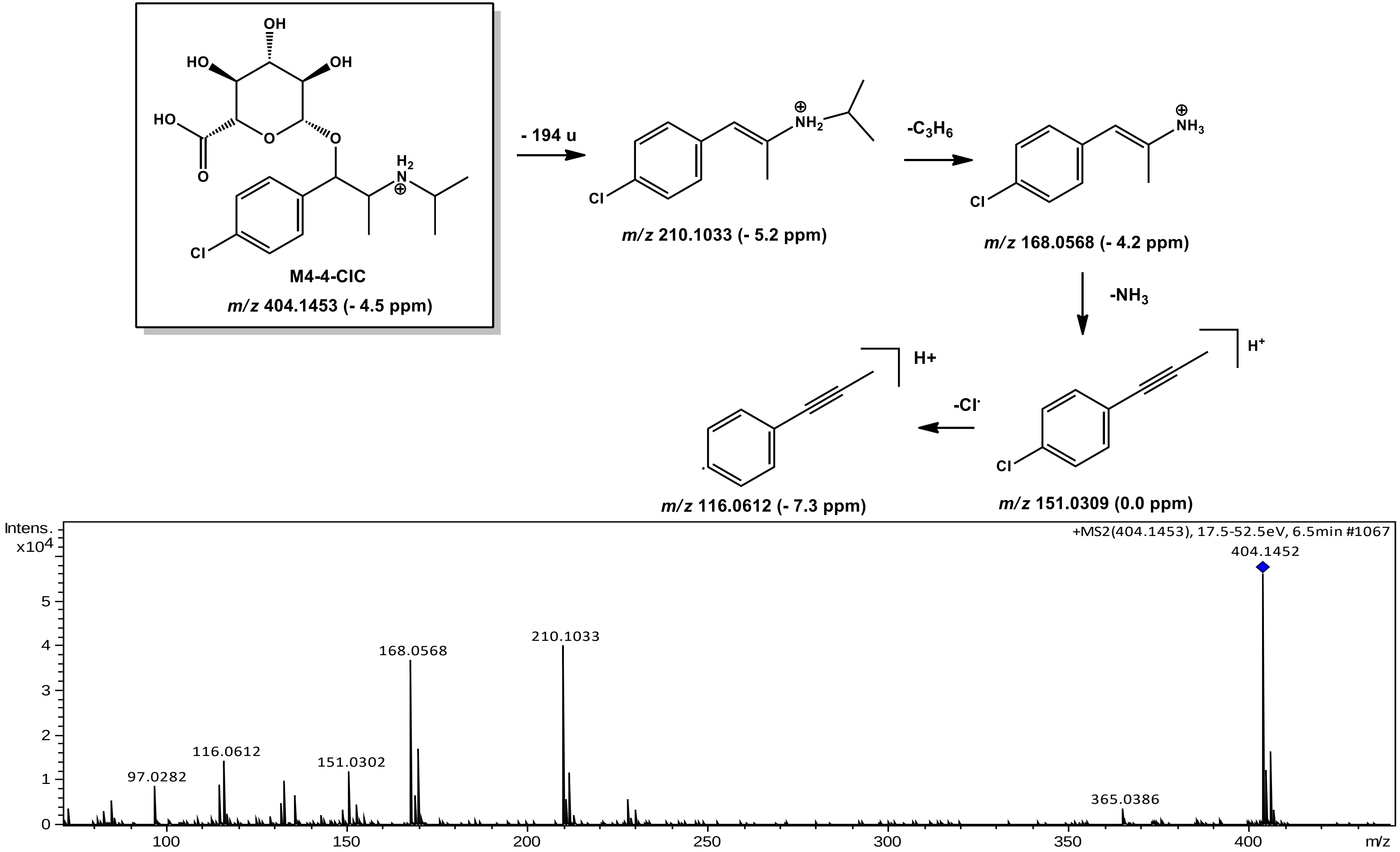

Figure S22: Tandem mass spectrum obtained for **M4-4-ClC** and proposed structures for the main fragment ions observed.

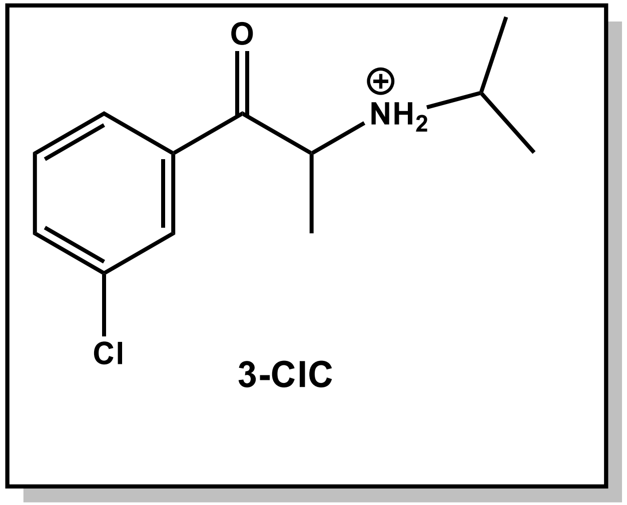

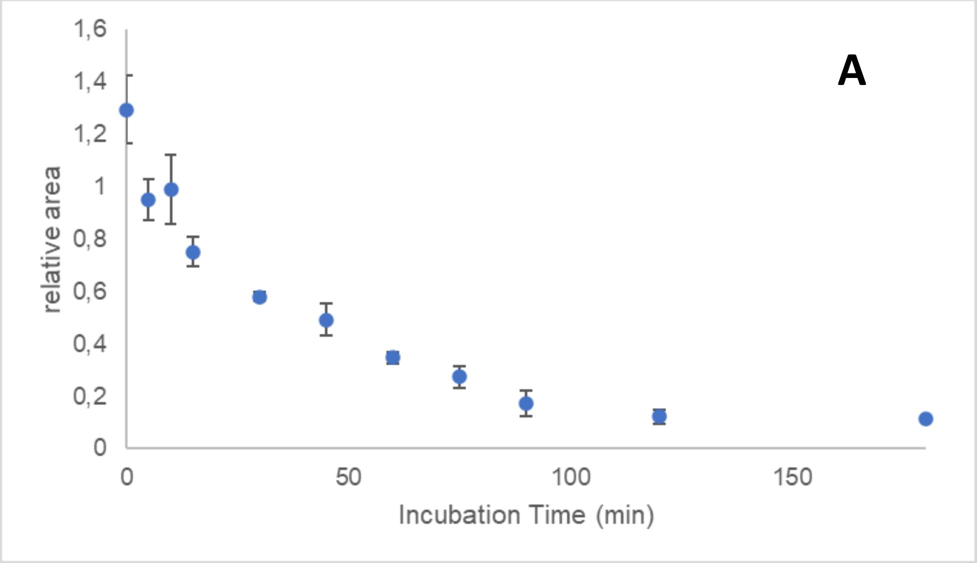

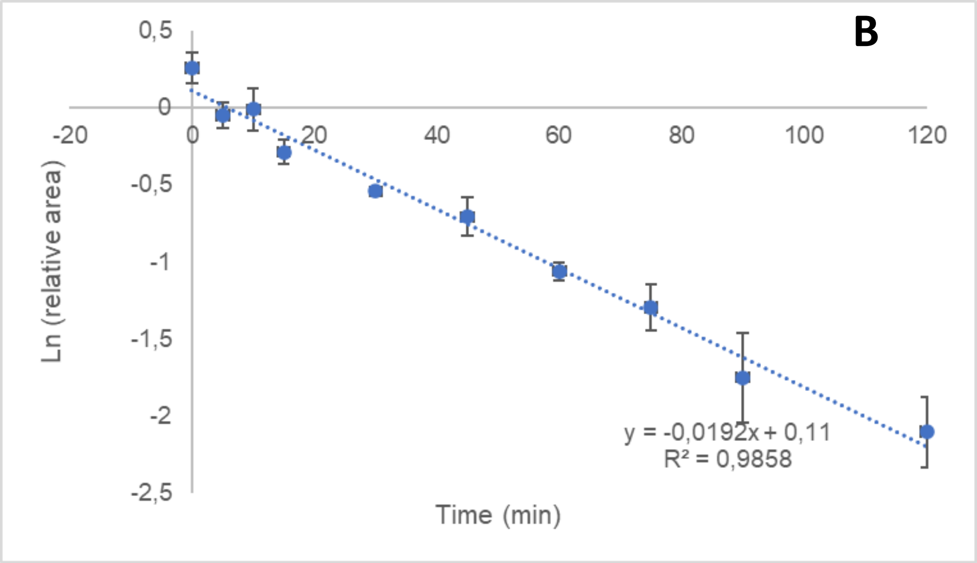

Figure S23: A. Depletion Plot; and **B.** ln (relative area) *vs* Time (min) obtained for **3-ClC** incubations in HLM.

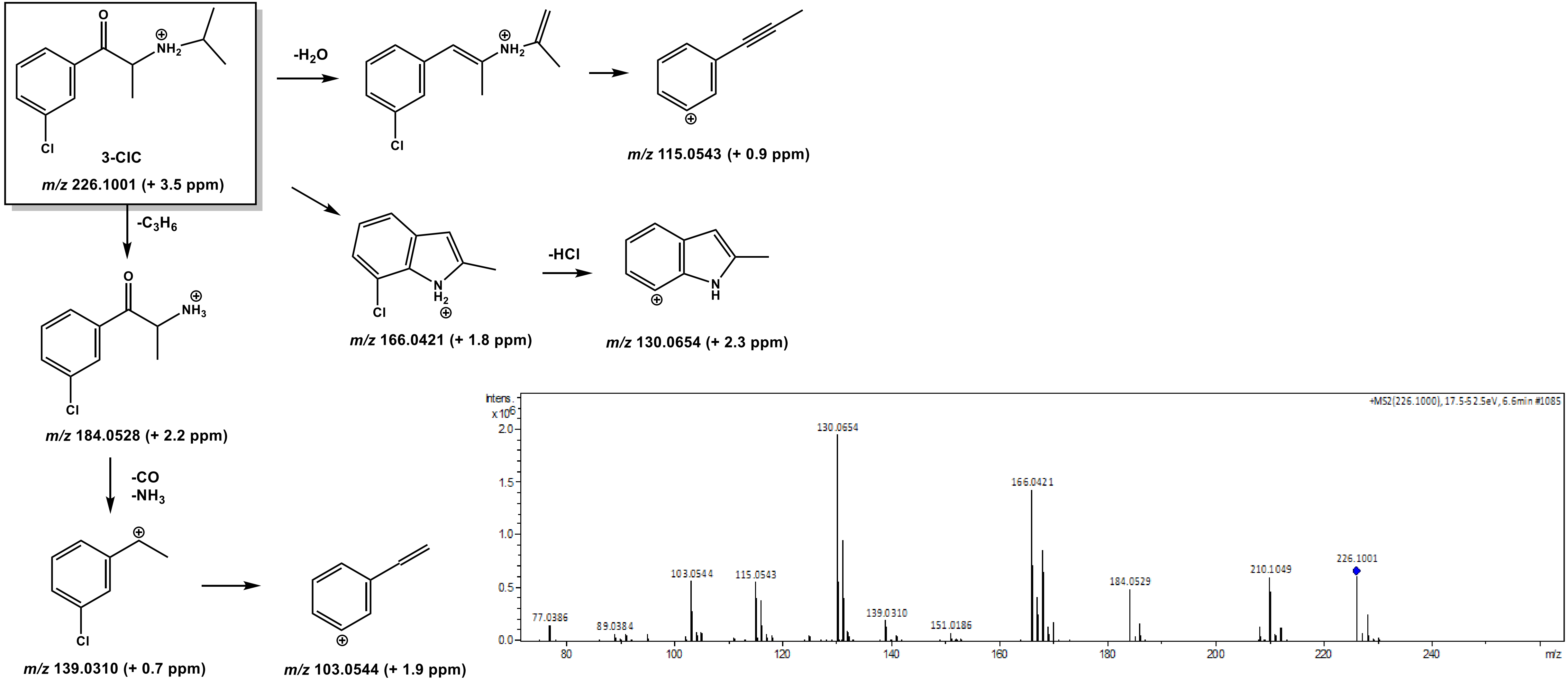

Figure S24: Tandem mass spectrum obtained for **3-ClC** and proposed structures for the main fragment ions observed.

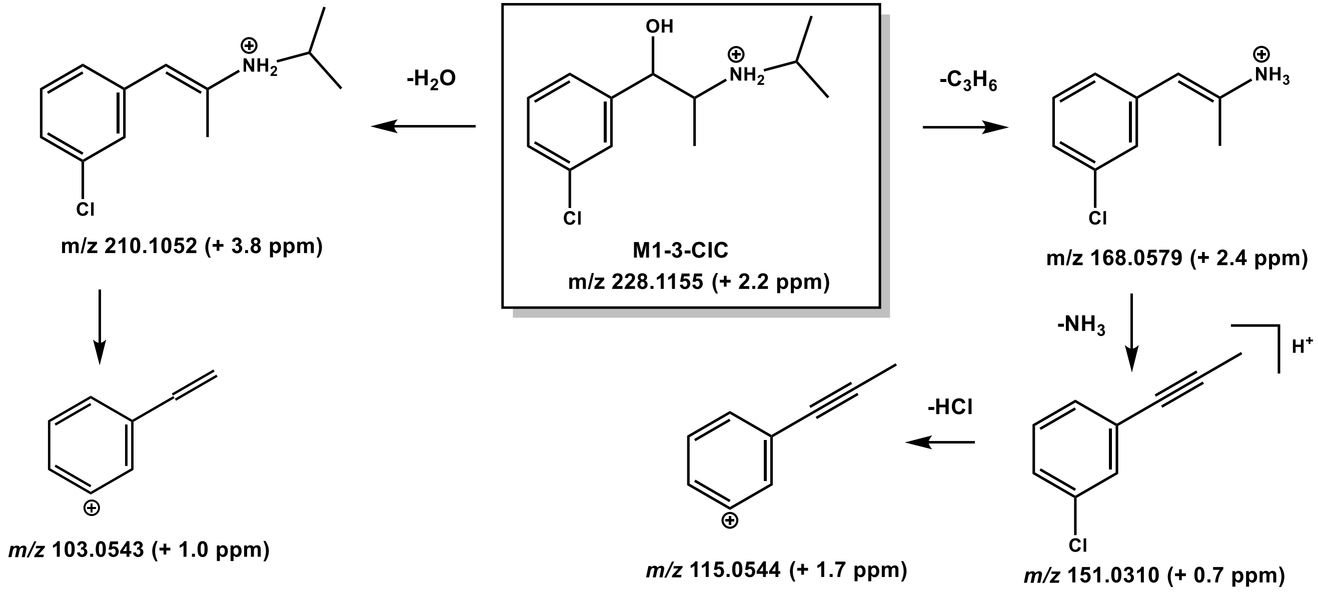

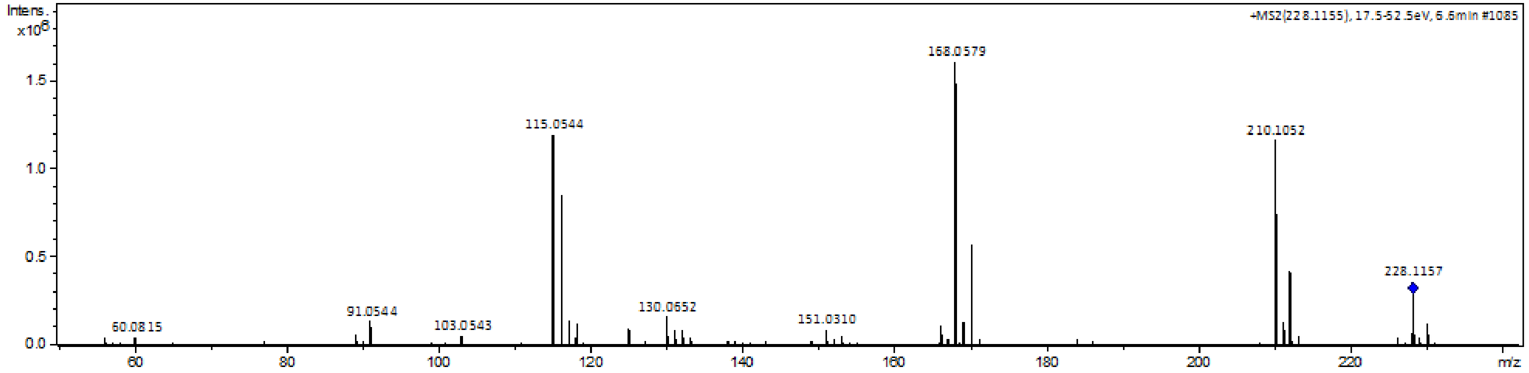

Figure S25: Tandem mass spectrum obtained for **M1-3-ClC** and proposed structures for the main fragment ions observed.

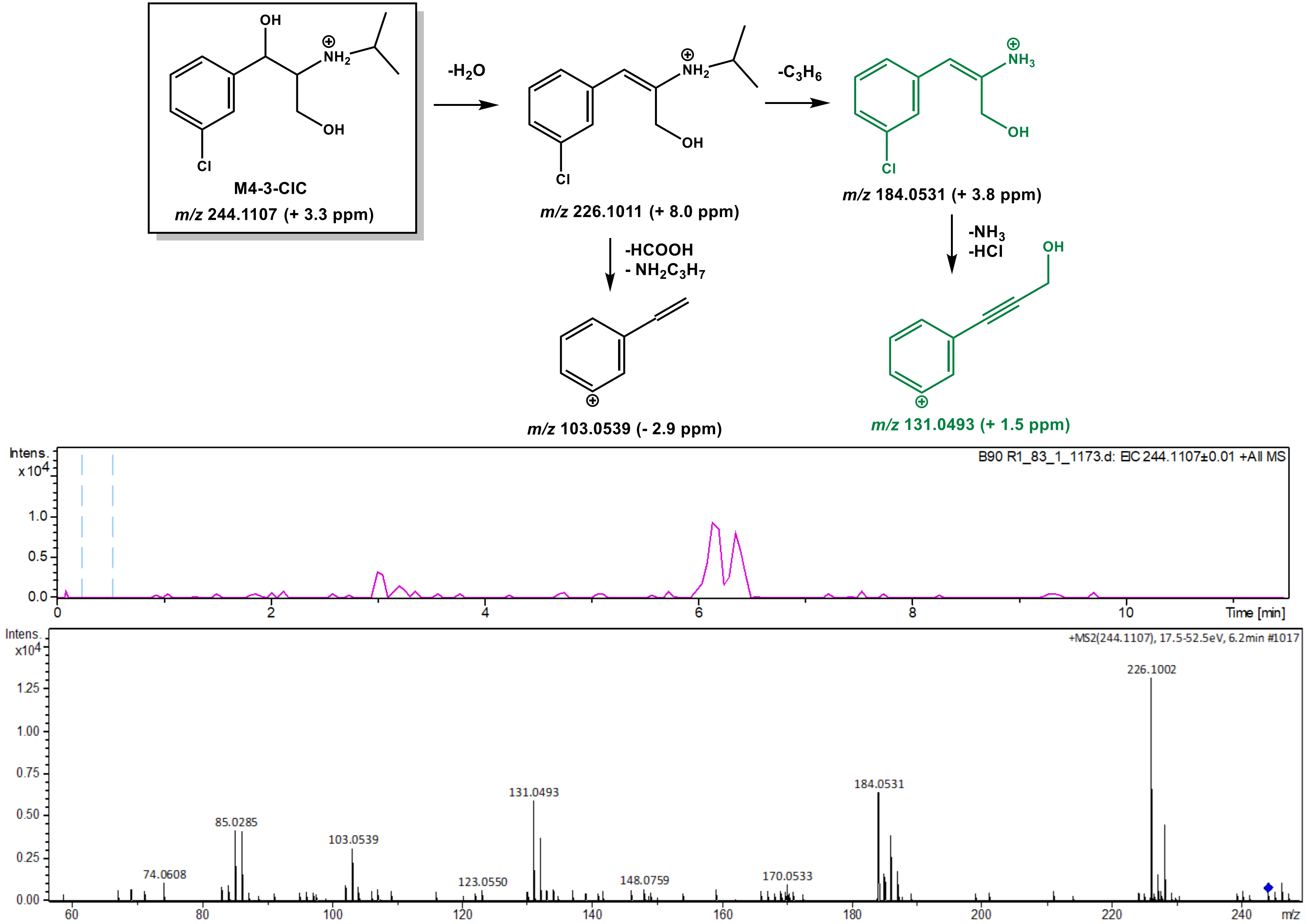

Figure S26: Tandem mass spectrum obtained for **M4-3-ClC** and proposed structures for the main fragment ions observed.

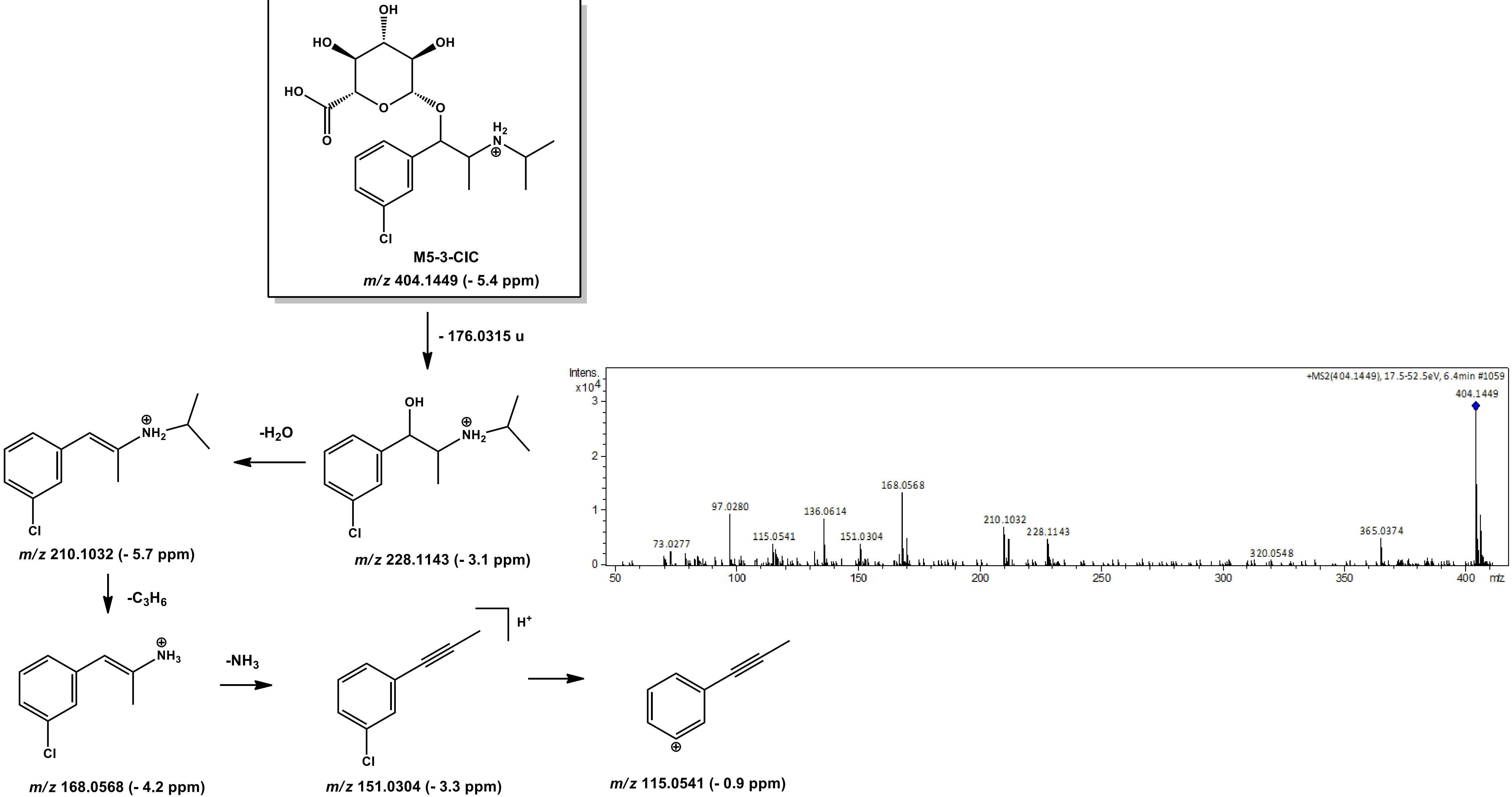

Figure S27: Tandem mass spectrum obtained for **M5-3-ClC** and proposed structures for the main fragment ions observed.

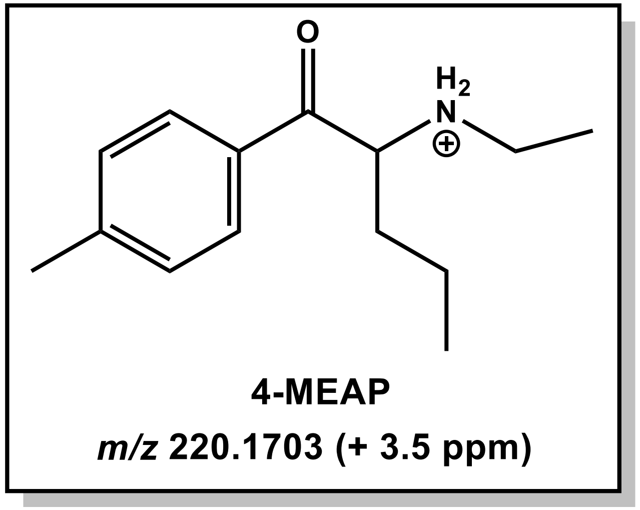

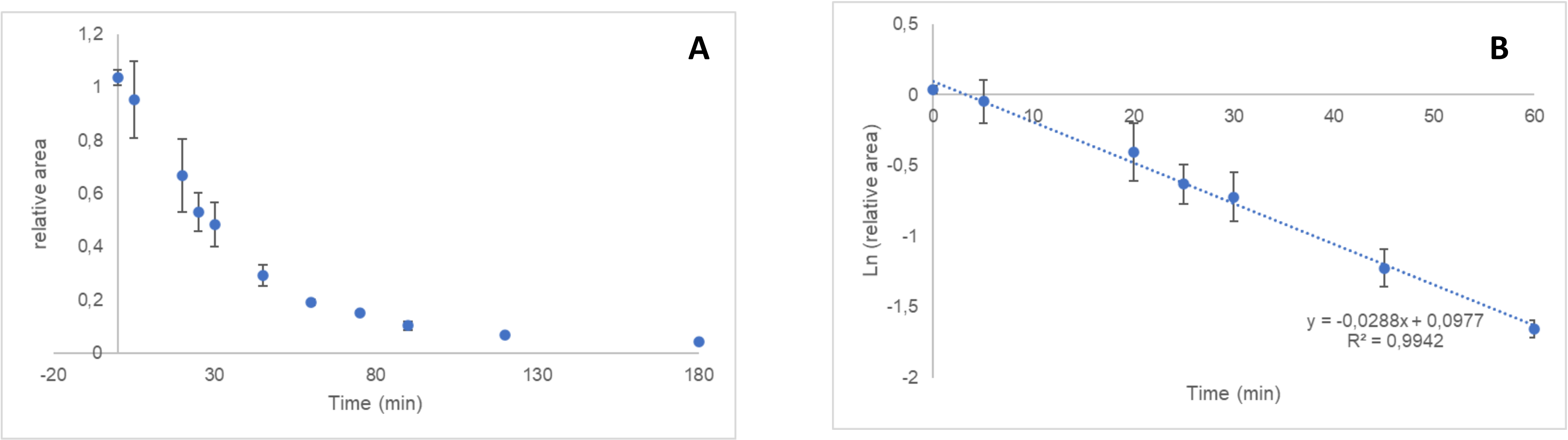

Figure S28: A. Depletion Plot: incubation time vs relative area; and **B.** Ln (relative area) *vs* time (min) obtained for **4-MEAP** incubations in HLM.

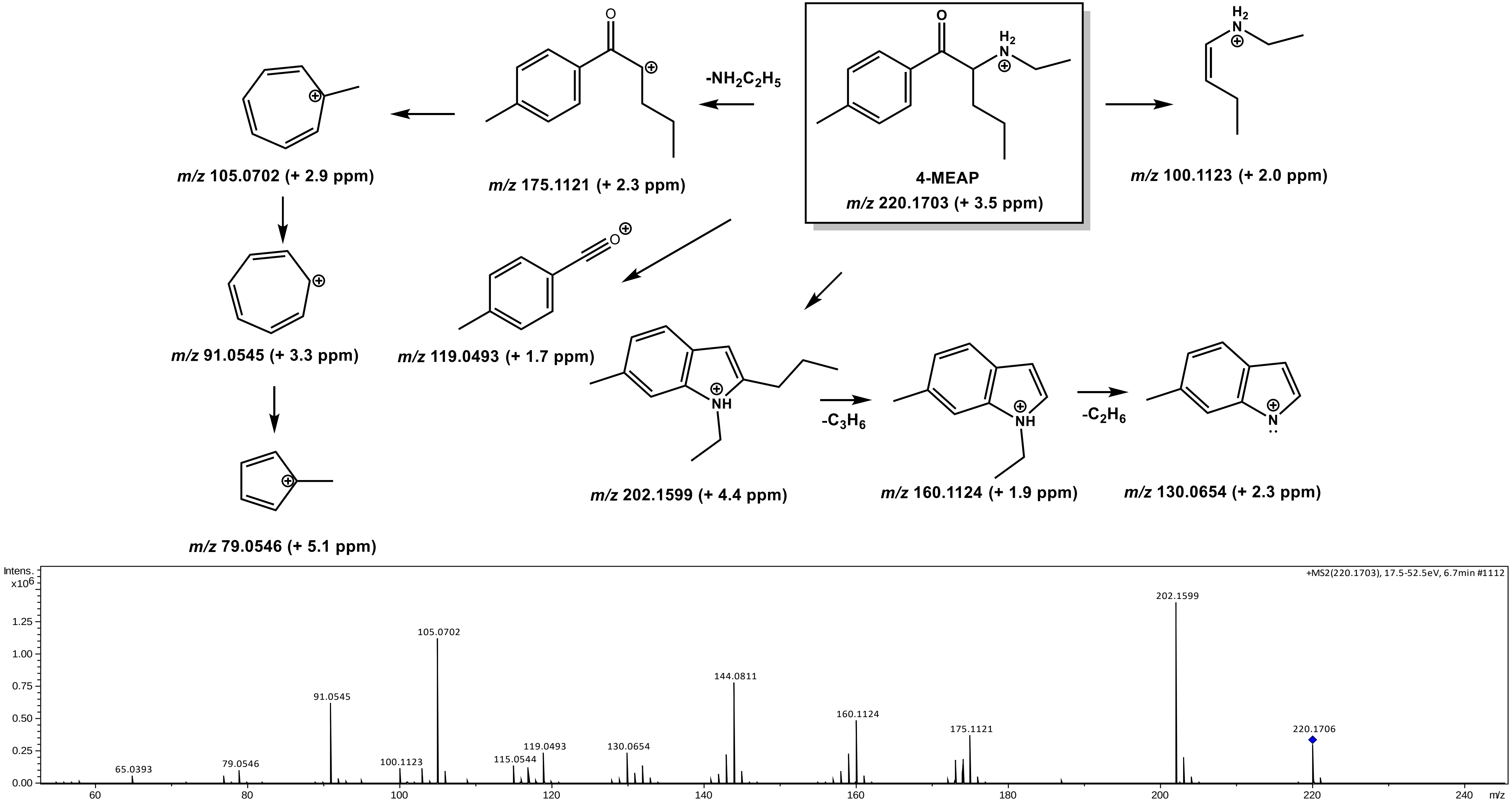

Figure S29: Tandem mass spectrum obtained for **4-MEAP** and proposed structures for the main fragment ions observed.

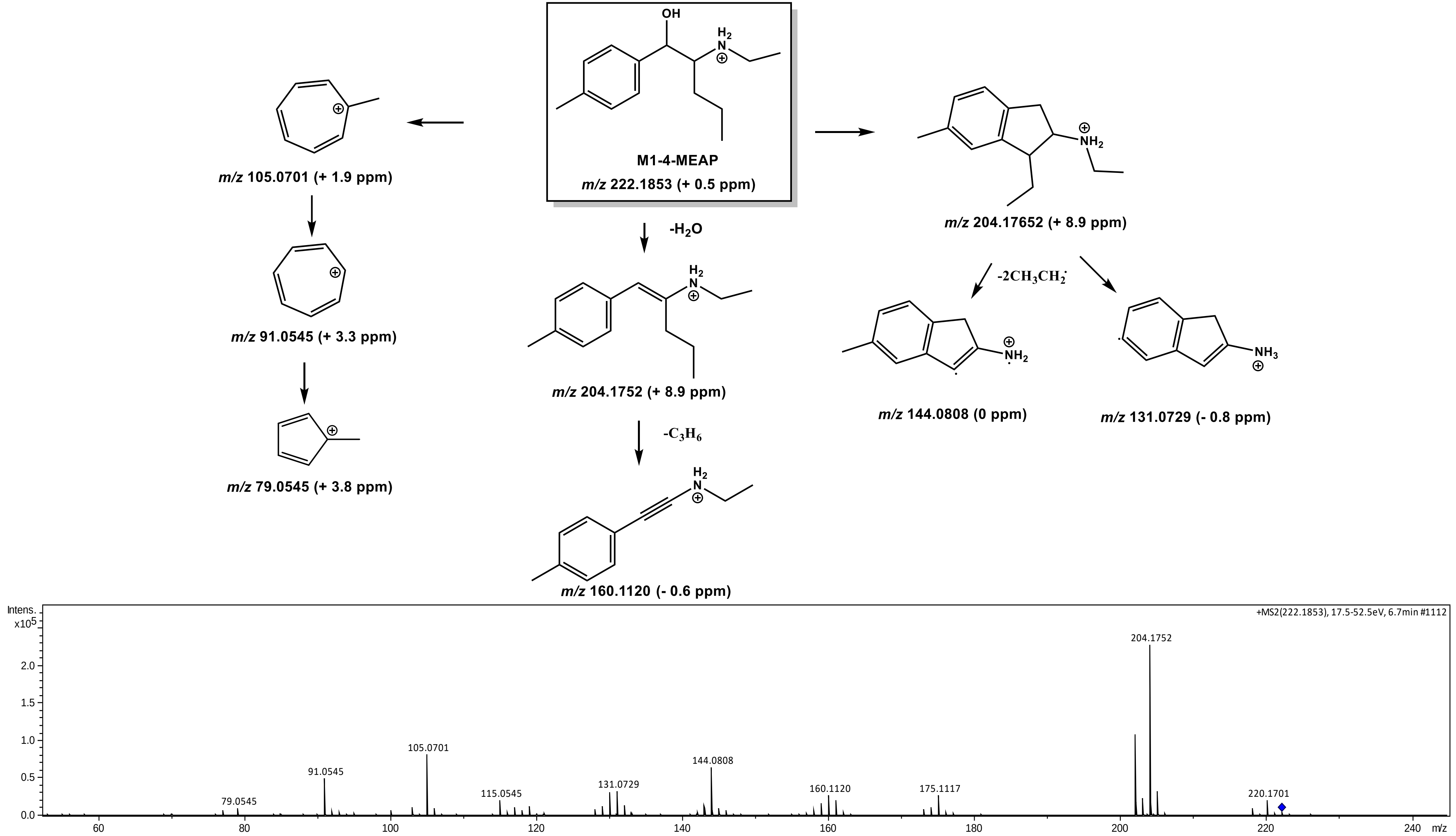

Figure S30: Tandem mass spectrum obtained for **M1-4-MEAP** and proposed structures for the main fragment ions observed.

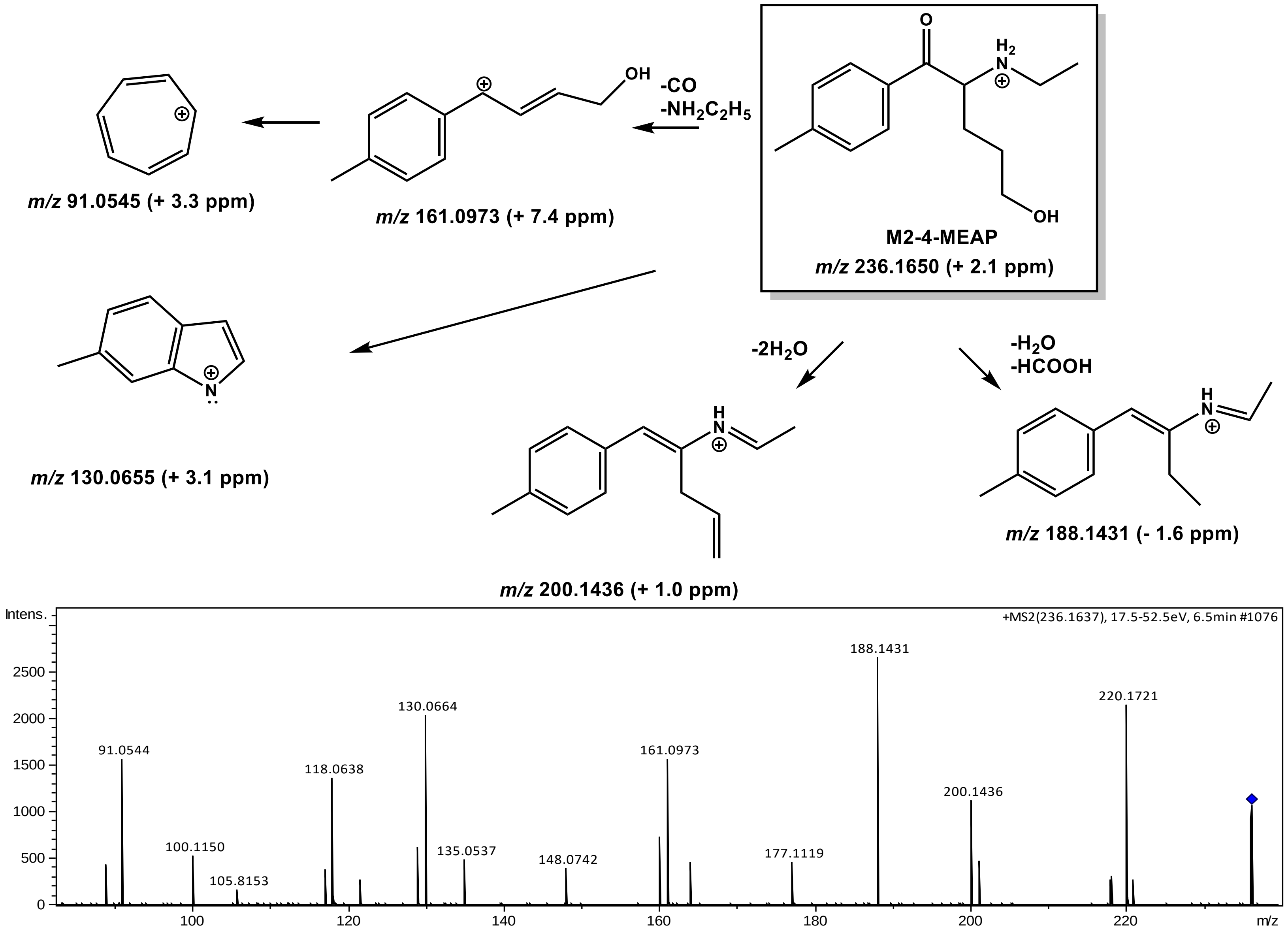

Figure S31: Tandem mass spectrum obtained for **M2-4-MEAP** and proposed structures for the main fragment ions observed.

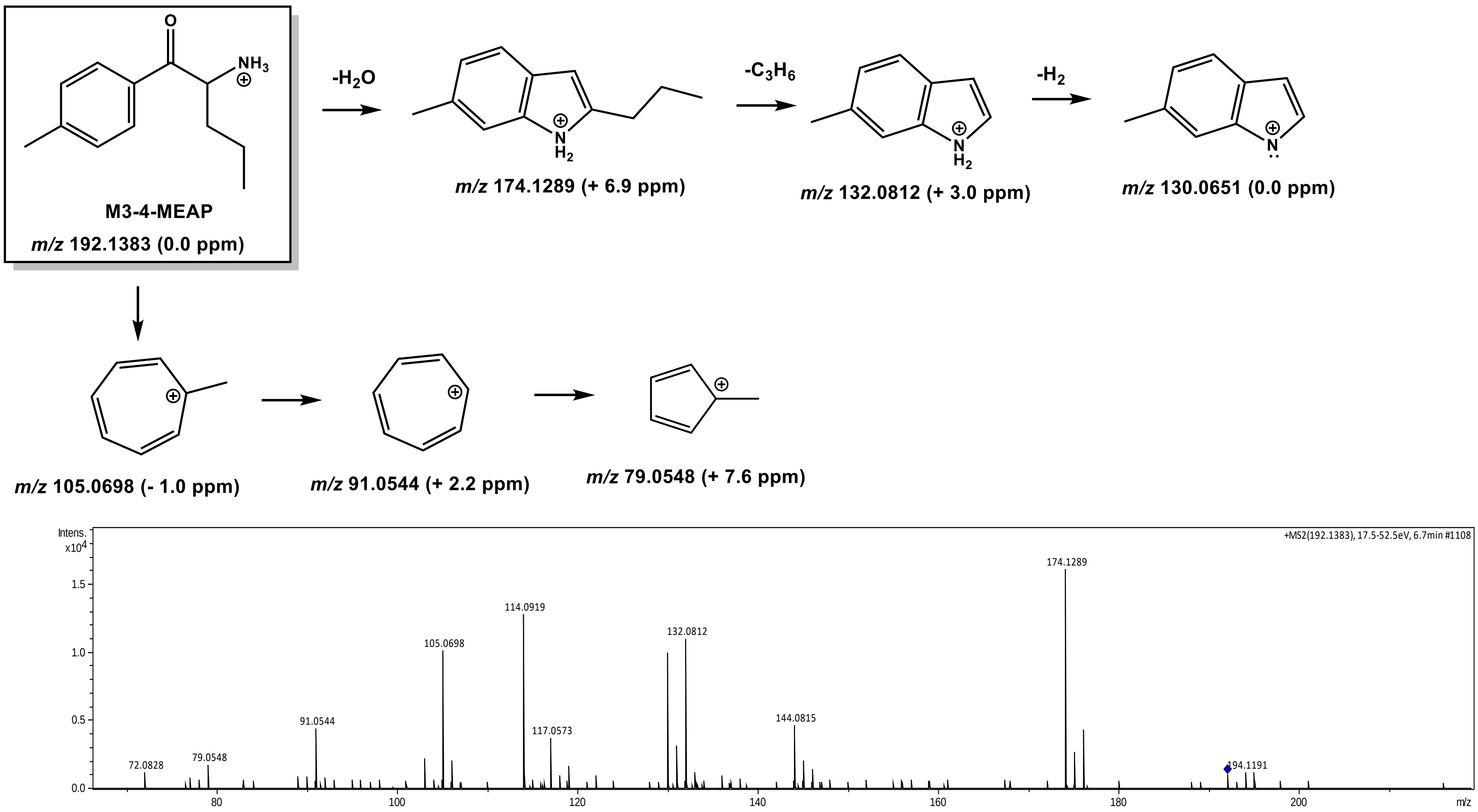

Figure S32: Tandem mass spectrum obtained for **M3-4-MEAP** and proposed structures for the main fragment ions observed.

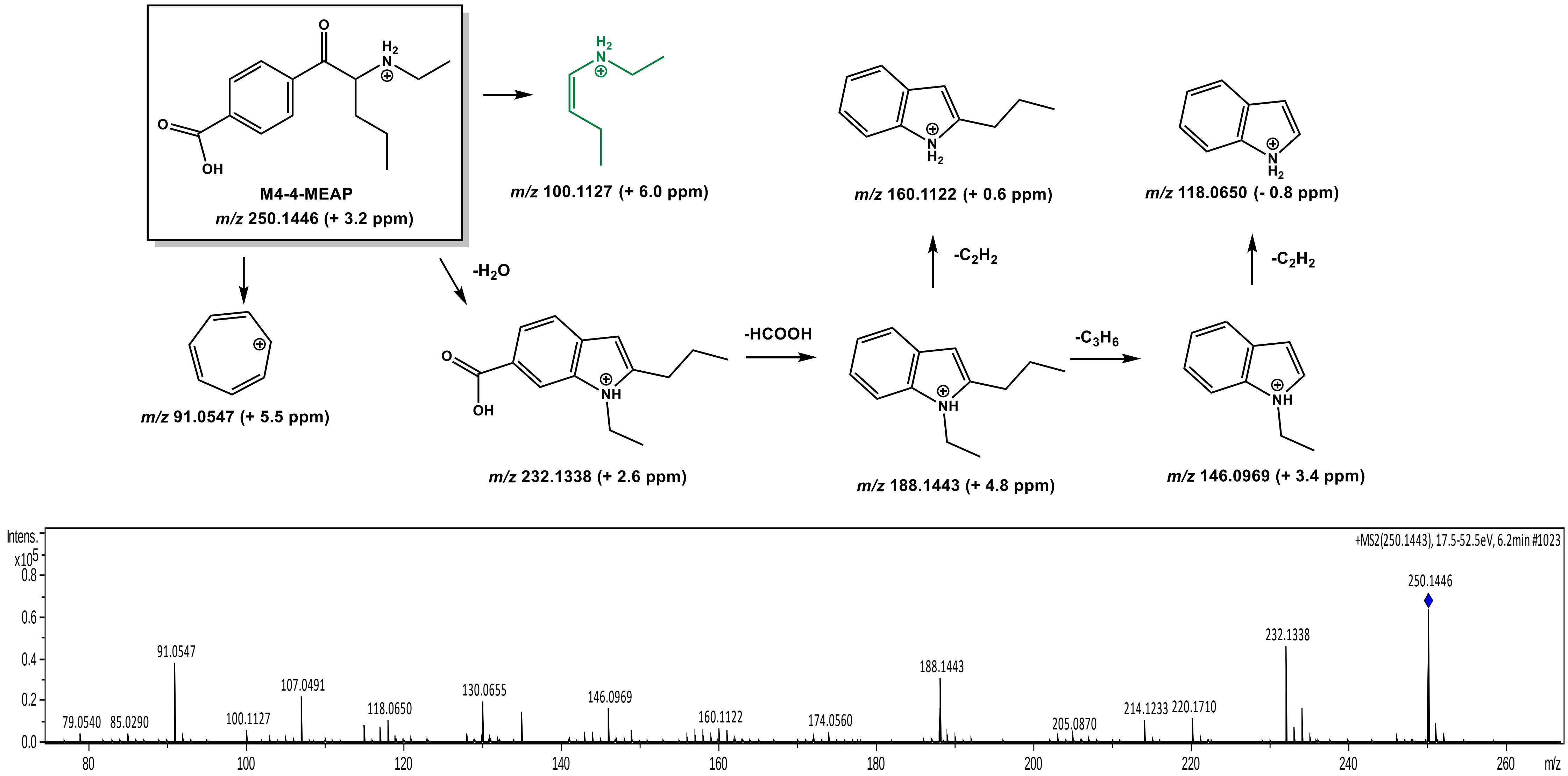

Figure S33: Tandem mass spectrum obtained for **M4-4-MEAP** and proposed structures for the main fragment ions observed.

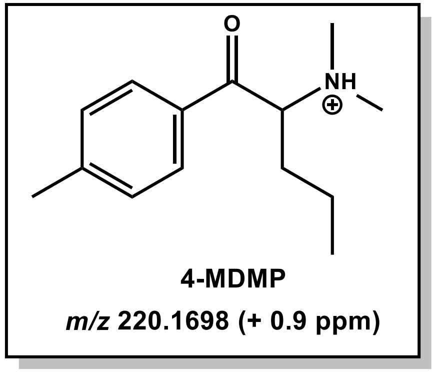

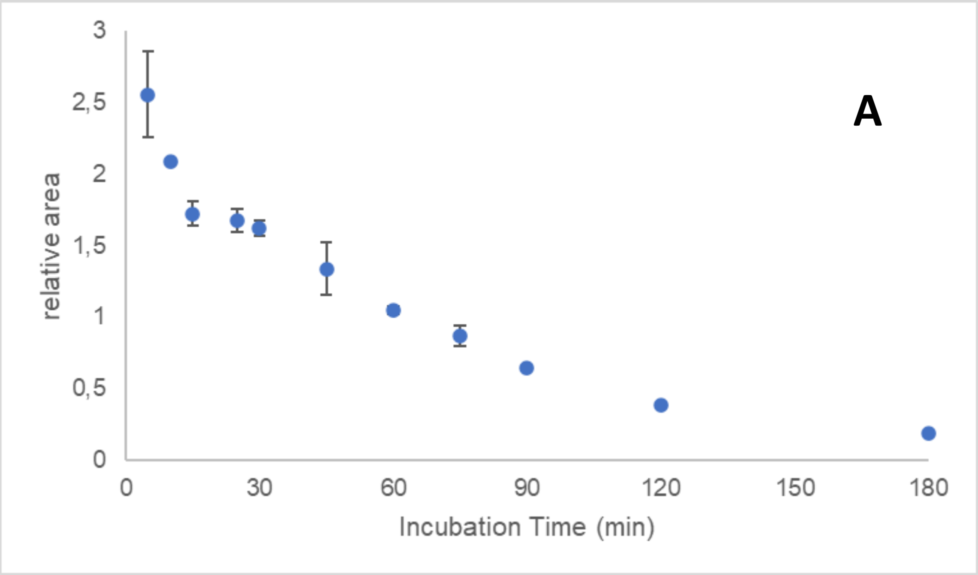

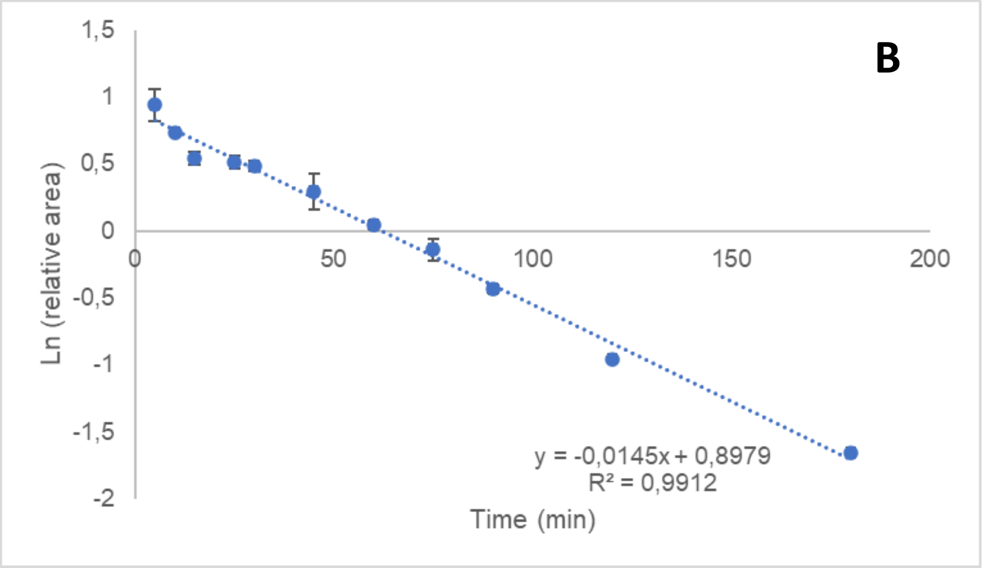

Figure S34: A. Depletion Plot; and **B.** Ln (relative area) *vs* Time (min) obtained for **4-MDMP** incubations in HLM.

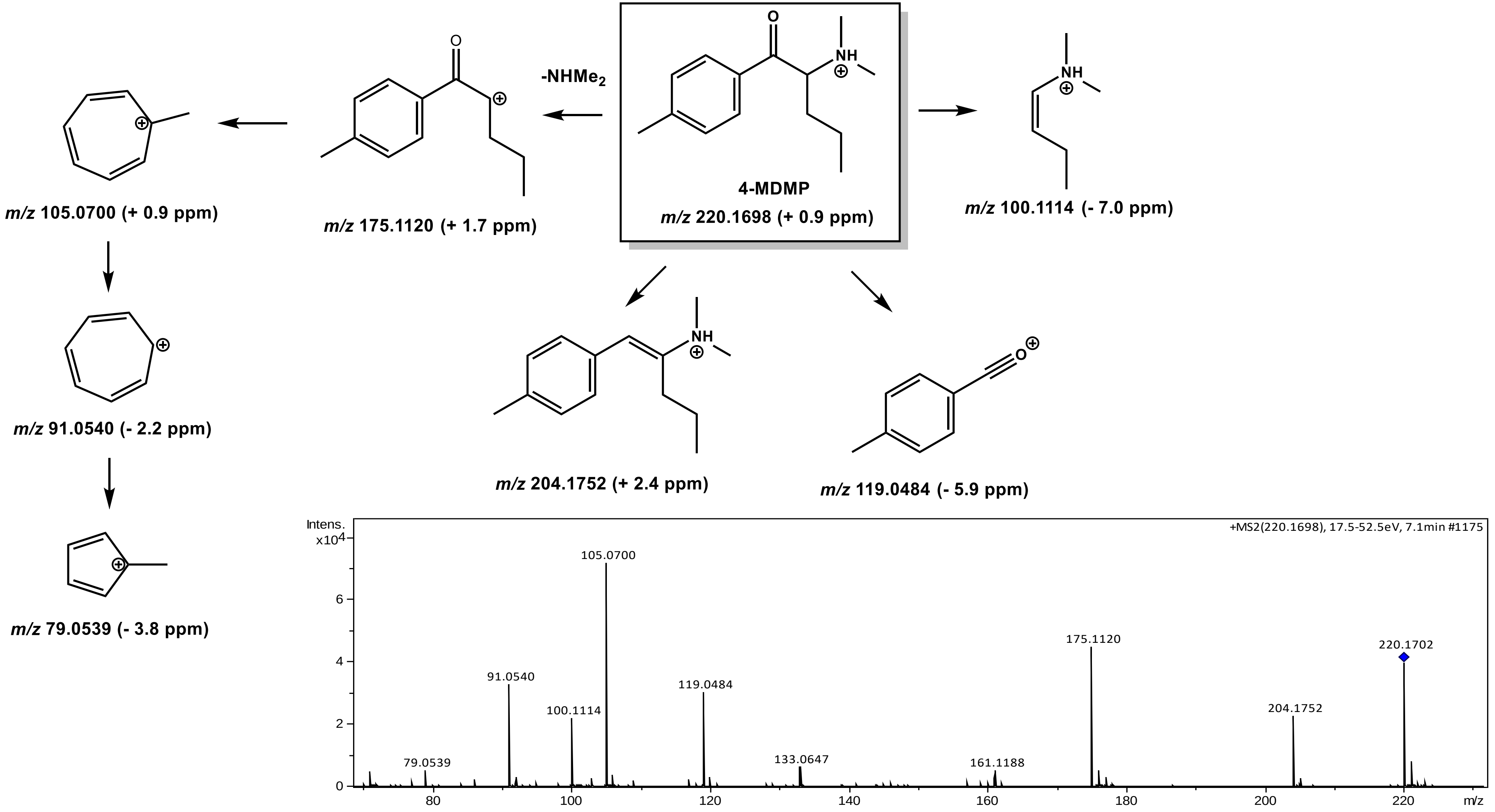

Figure S35: Tandem mass spectrum obtained for **4-MDMP** and proposed structures for the main fragment ions observed.

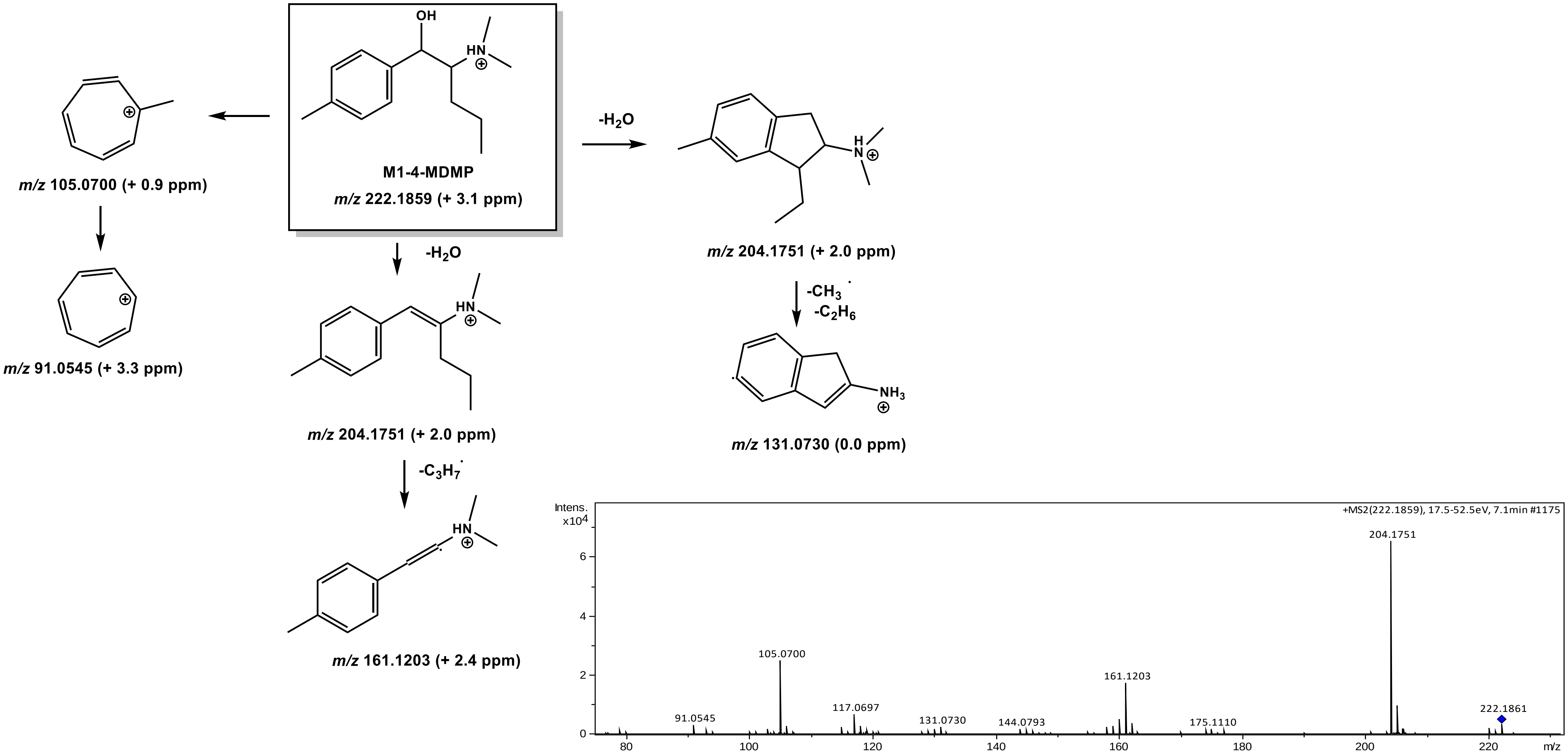

Figure S36: Tandem mass spectrum obtained for **M1-4-MDMP** and proposed structures for the main fragment ions observed.

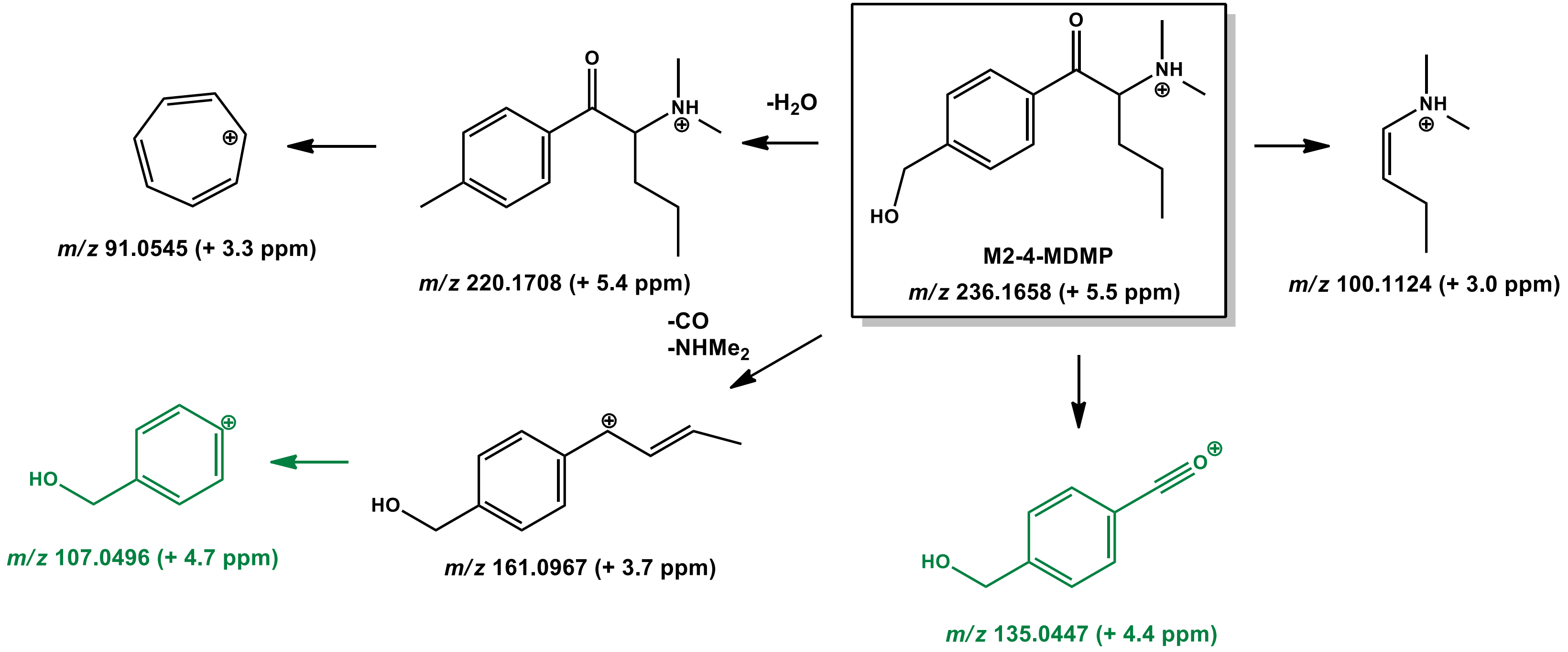

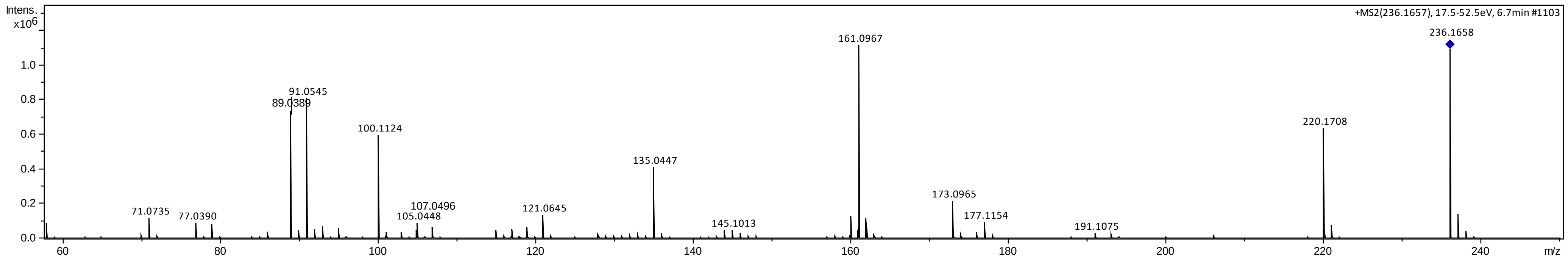

Figure S37: Tandem mass spectrum obtained for **M2-4-MDMP** and proposed structures for the main fragment ions observed. Highlighted in green are the diagnostic ions

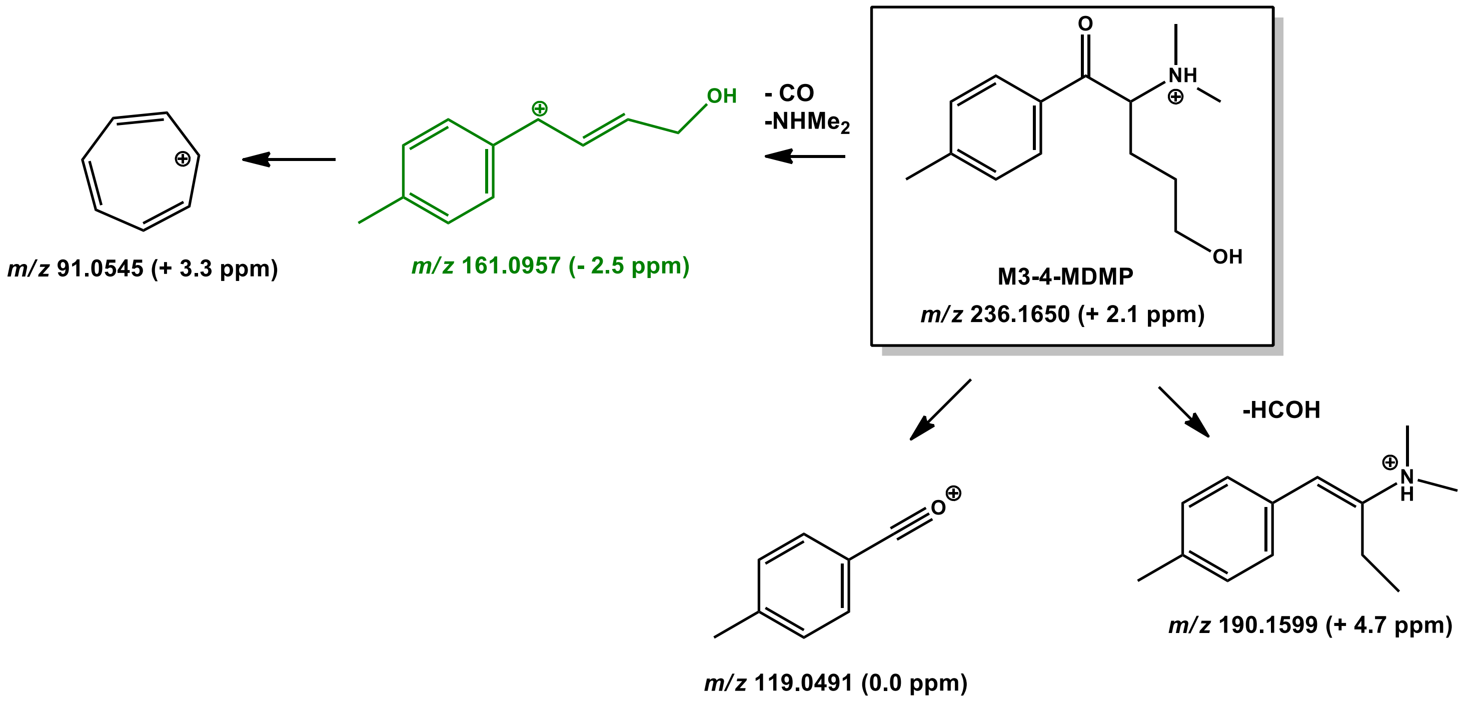

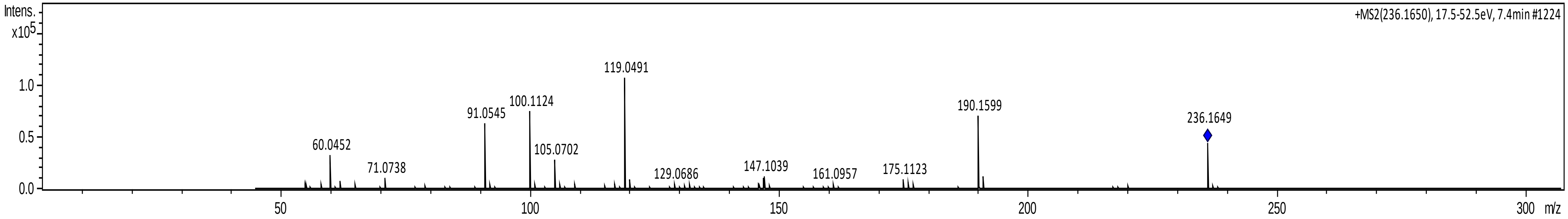

Figure S38: Tandem mass spectrum obtained for **M3-4-MDMP** and proposed structures for the main fragment ions observed. Highligted in gree is the diagnostic ion

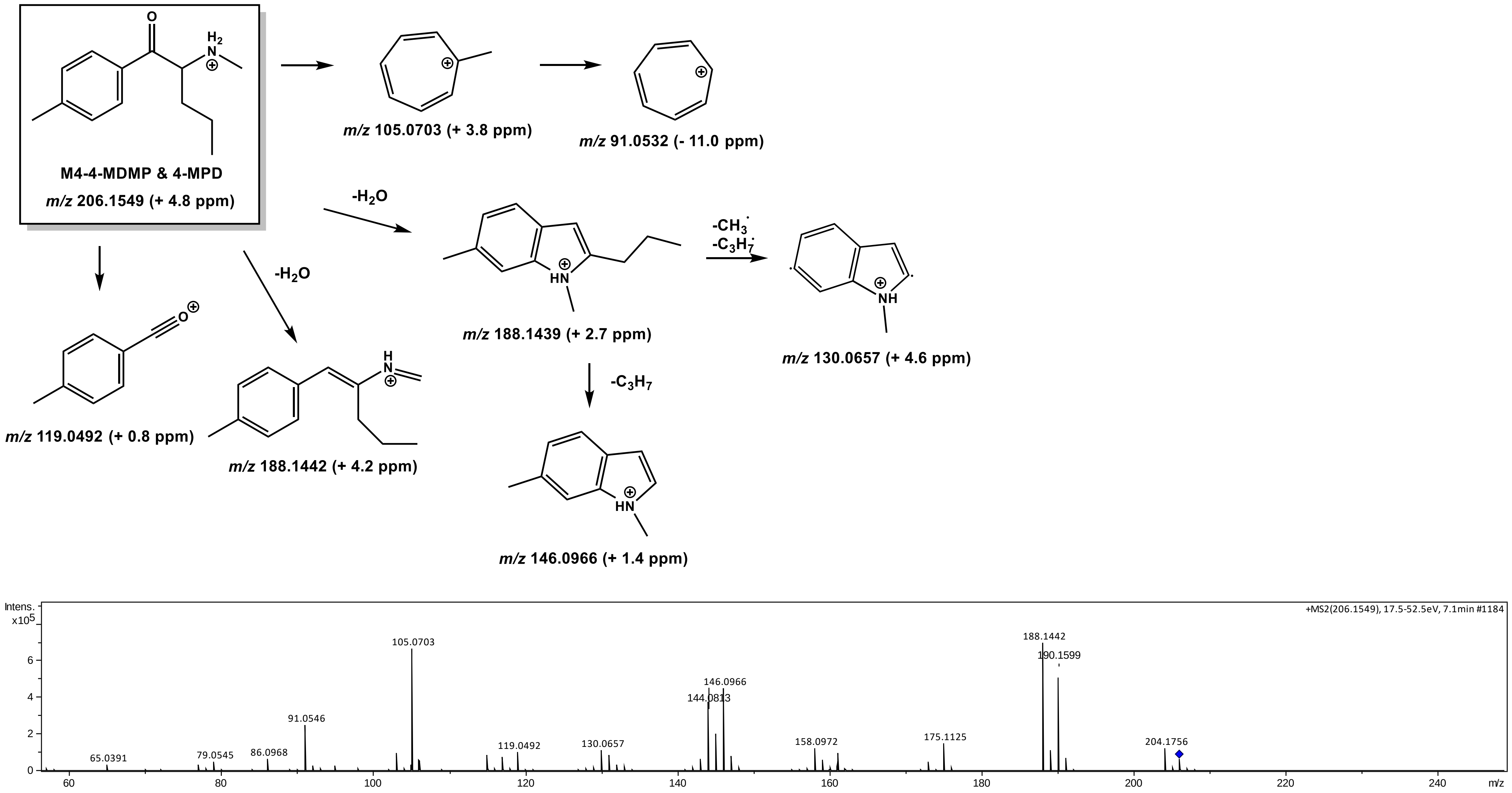

Figure S39: Tandem mass spectrum obtained for **M4-4-MDMP** and proposed structures for the main fragment ions observed.

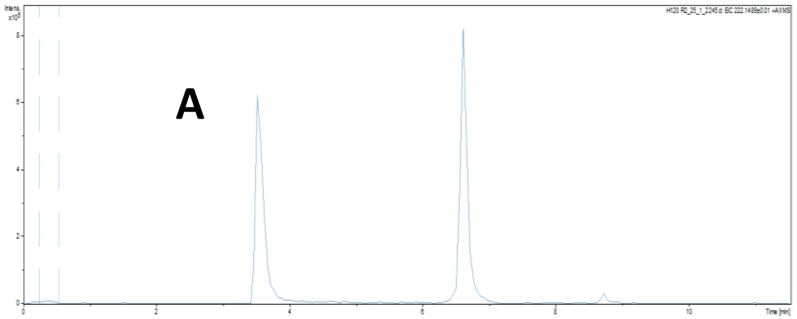

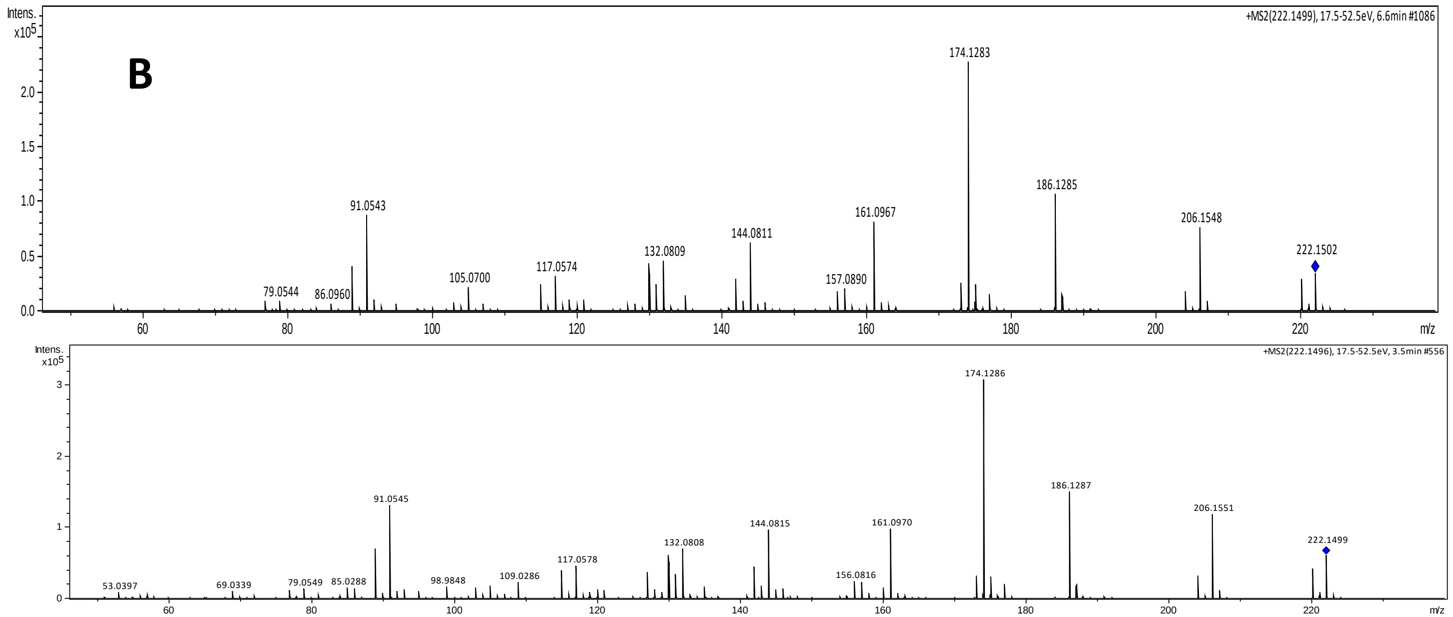

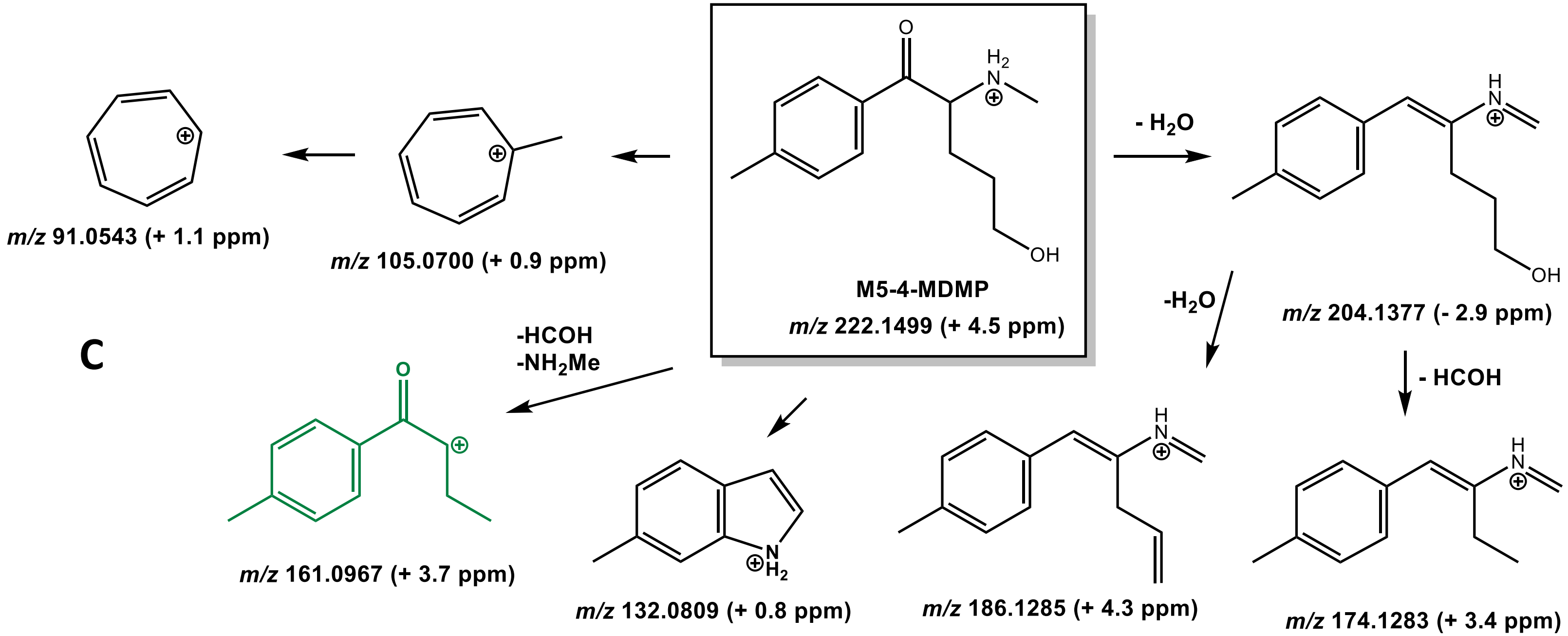

Figure S40: A. Extracted ion chromatogram; **B.** Tandem mass spectrum obtained for **M5-4-MDMP;** and **C**. Proposed structures for the main fragment ions observed.

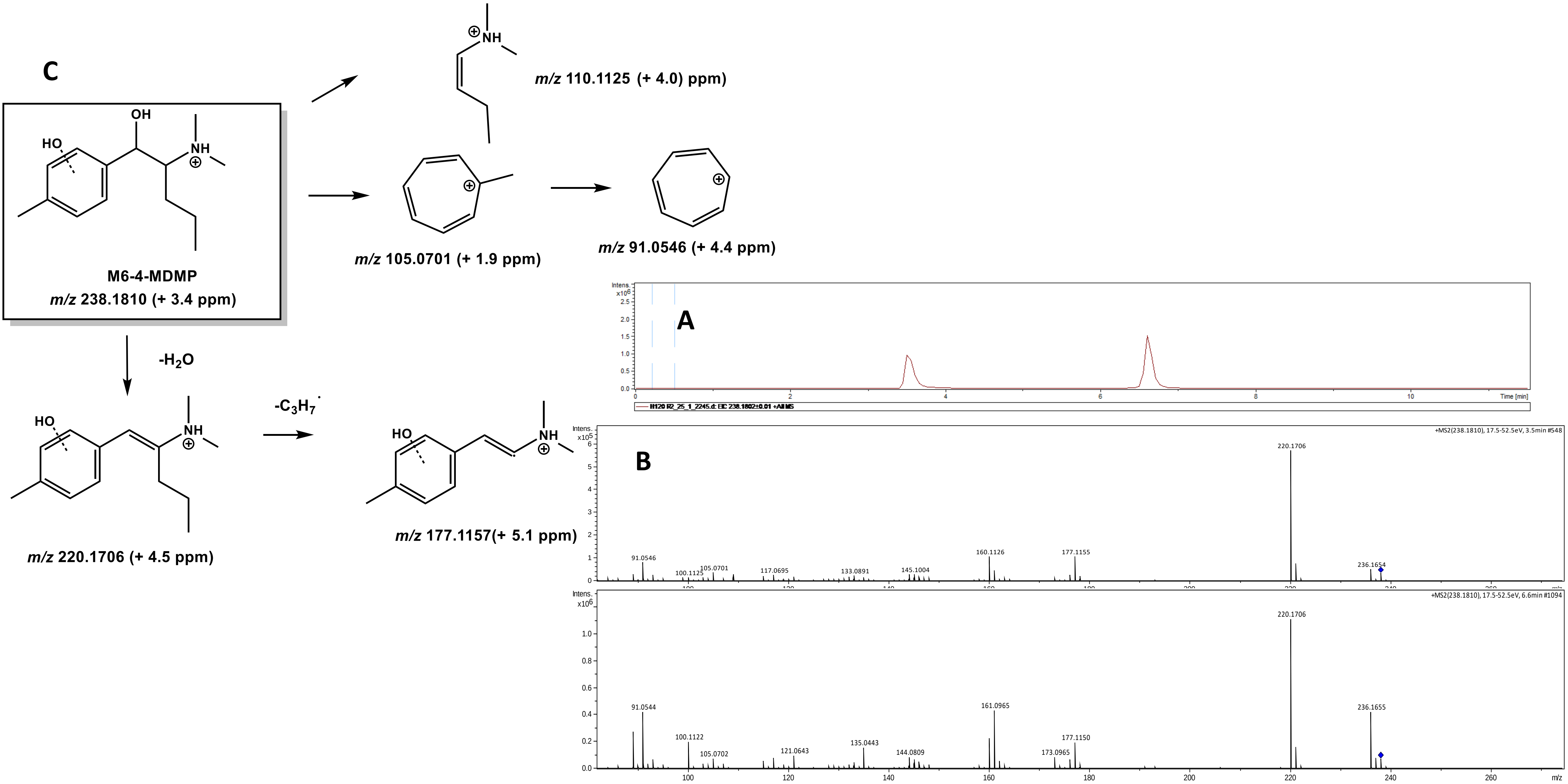

Figure S41: A. Extracted ion chromatogram at *m/z* 238.1810; **B.** Tandem mass spectra obtained for the isobaric ions **M6-4-MDMPa and M6-4-MDMPa;** and **C.** proposed structures for the main fragment ions observed.

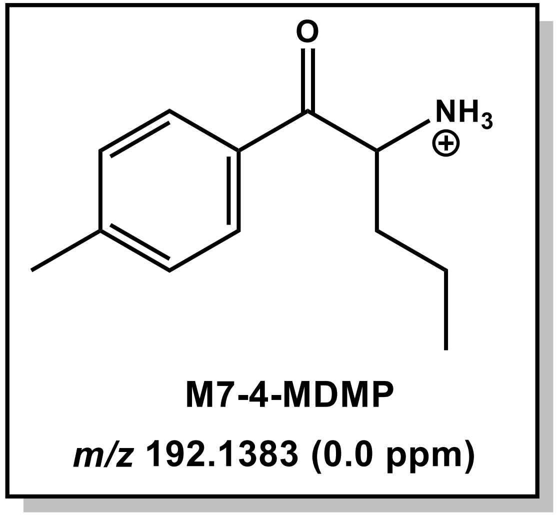

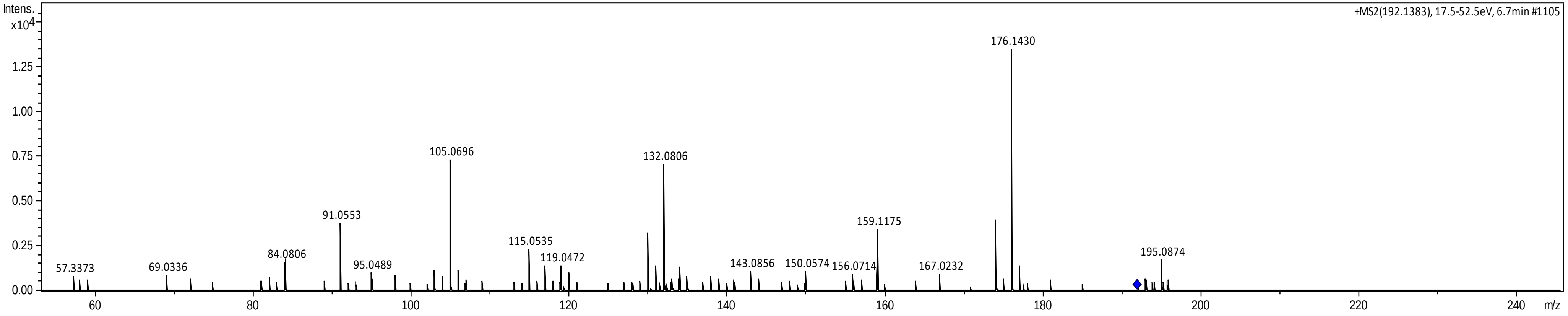

Figure S42: Tandem mass spectrum obtained for **M7-4-MDMP** and proposed structures for the main fragment ions observed.

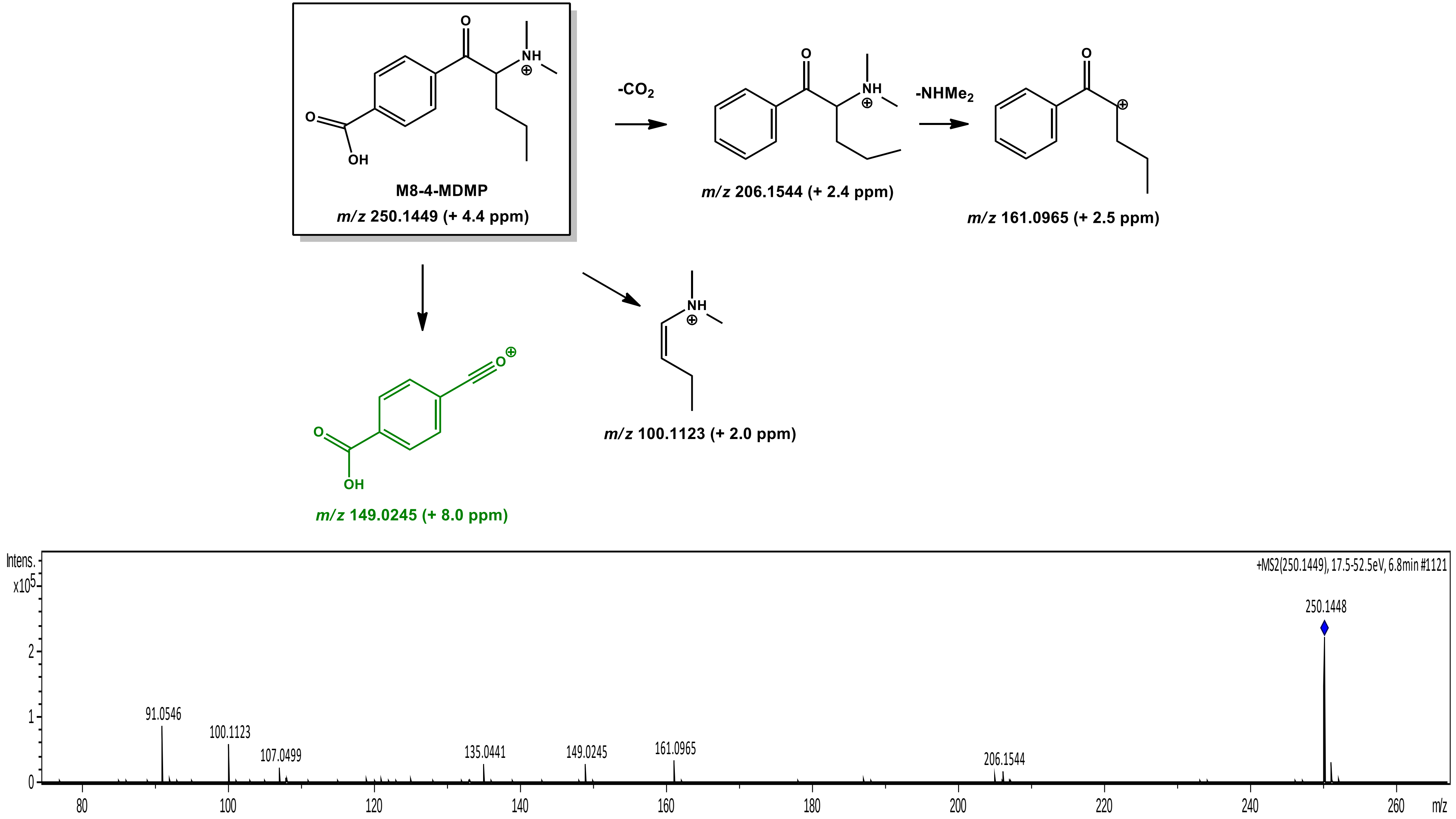

Figure S43: Tandem mass spectrum obtained for **M8-4-MDMP** and proposed structures for the main fragment ions observed.

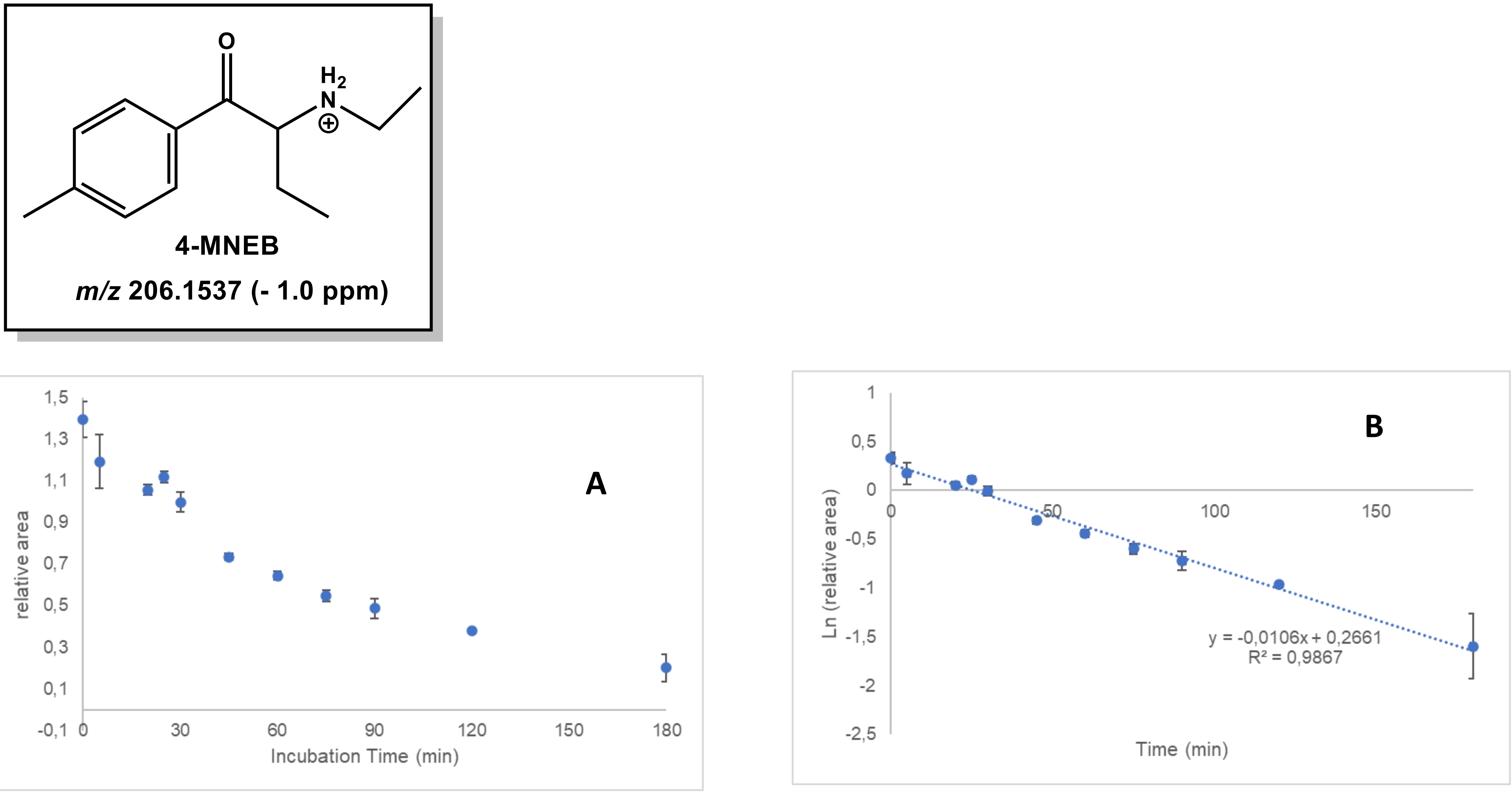

Figure S44: A. Depletion Plot; and **B.** Ln (relative area) *vs* Time (min) obtained for **4-MNEB** incubations in HLM.

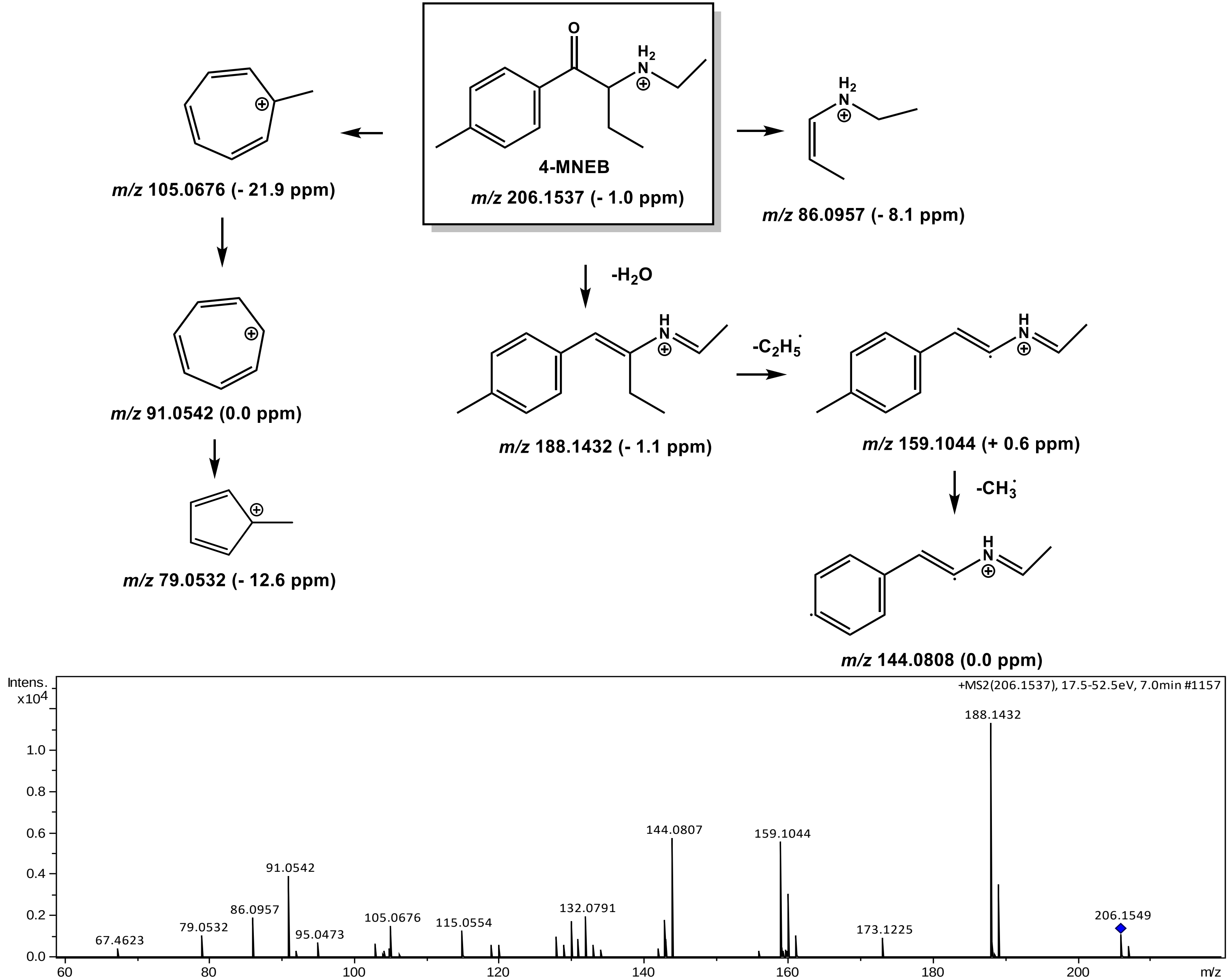

Figure S45: Tandem mass spectrum obtained for **4-MNEB** and proposed structures for the main fragment ions observed.

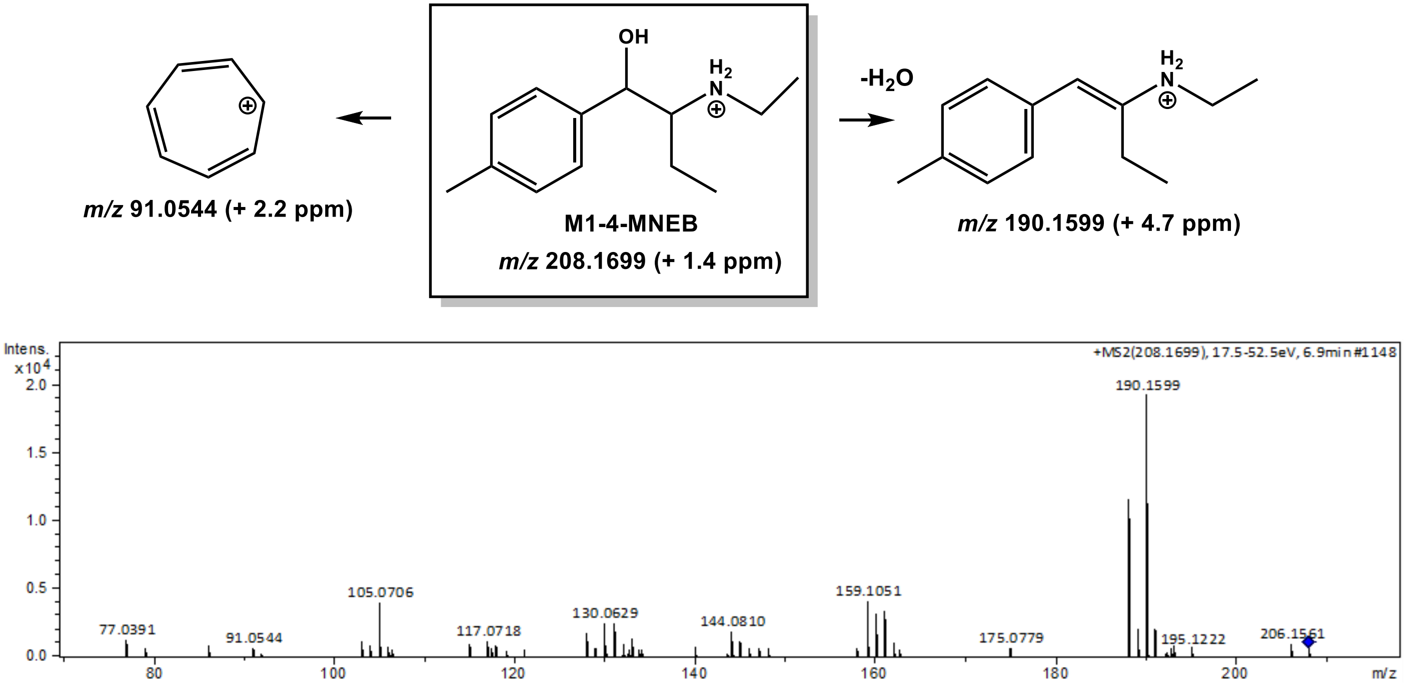

Figure S46: Tandem mass spectrum obtained for **M1-4-MNEB** and proposed structures for the main fragment ions observed.

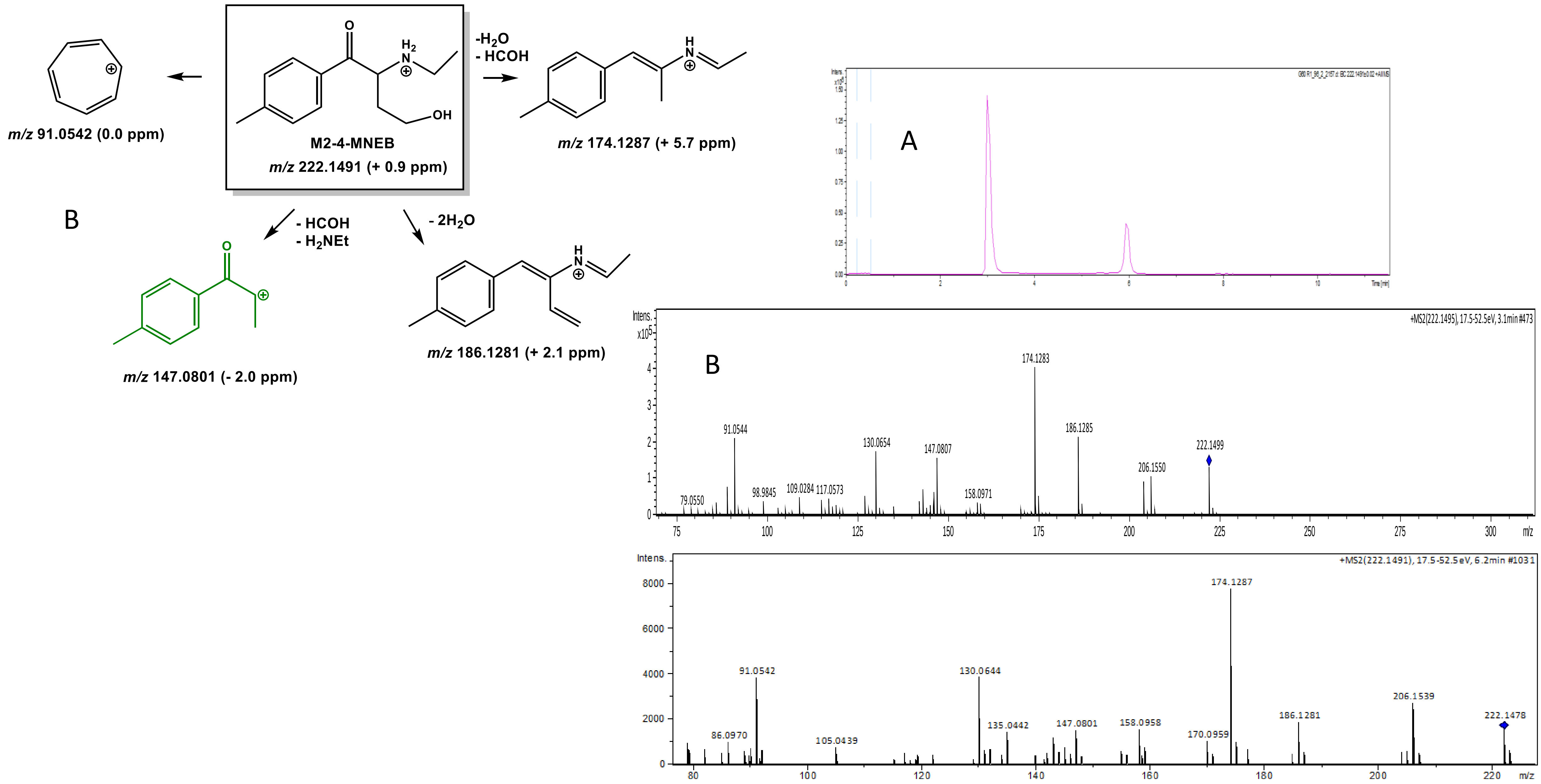

Figure S47: A. Extracted ion chromatogram; **B.** Tandem mass spectrum obtained for **M2-4-MNEB;** and **C.** proposed structures for the main fragment ions observed.

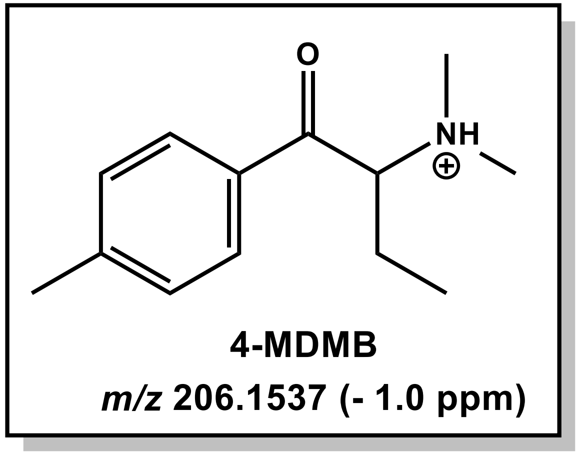

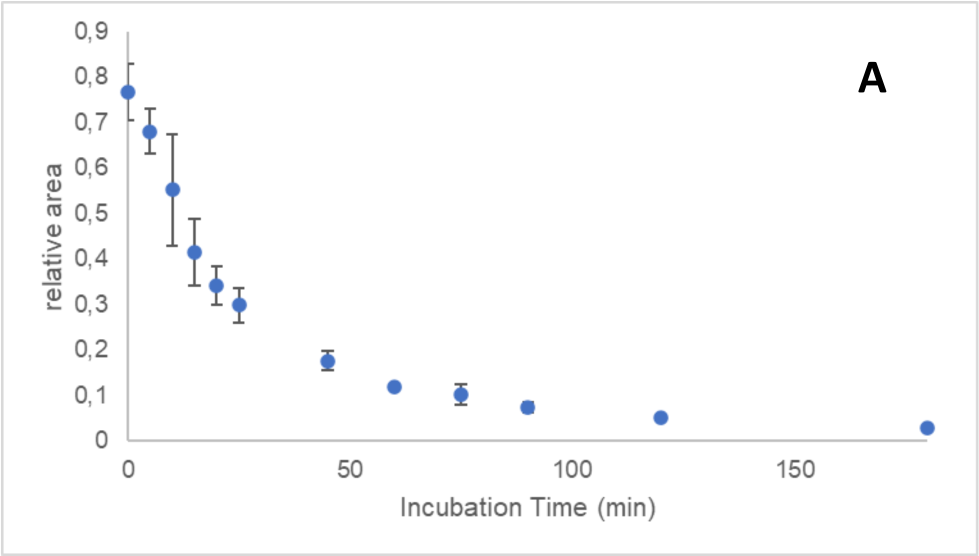

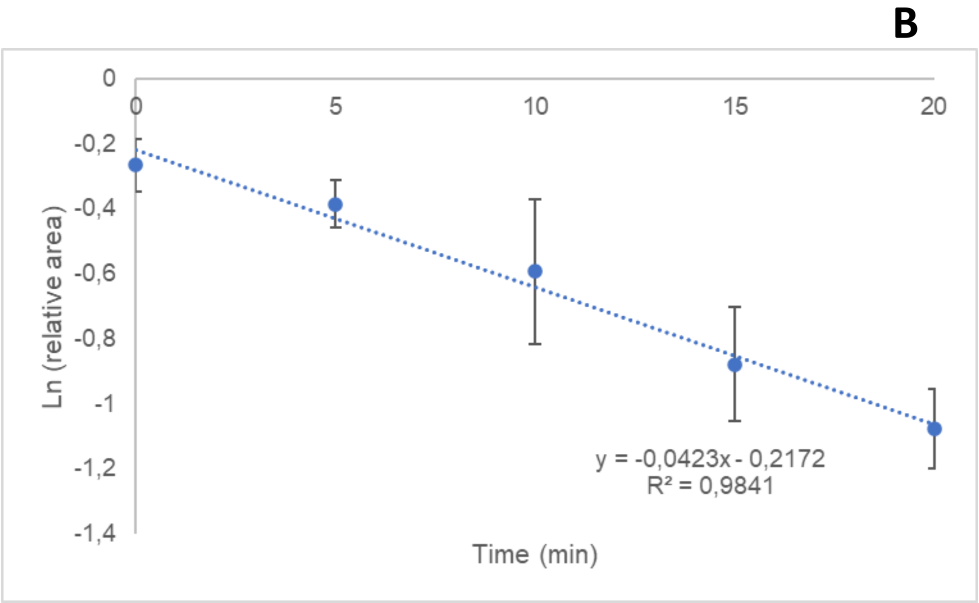

Figure S48: A. Depletion Plot; and **B.** ln (relative area) *vs* Time (min) obtained for **4-MDMB** incubations in HLM.

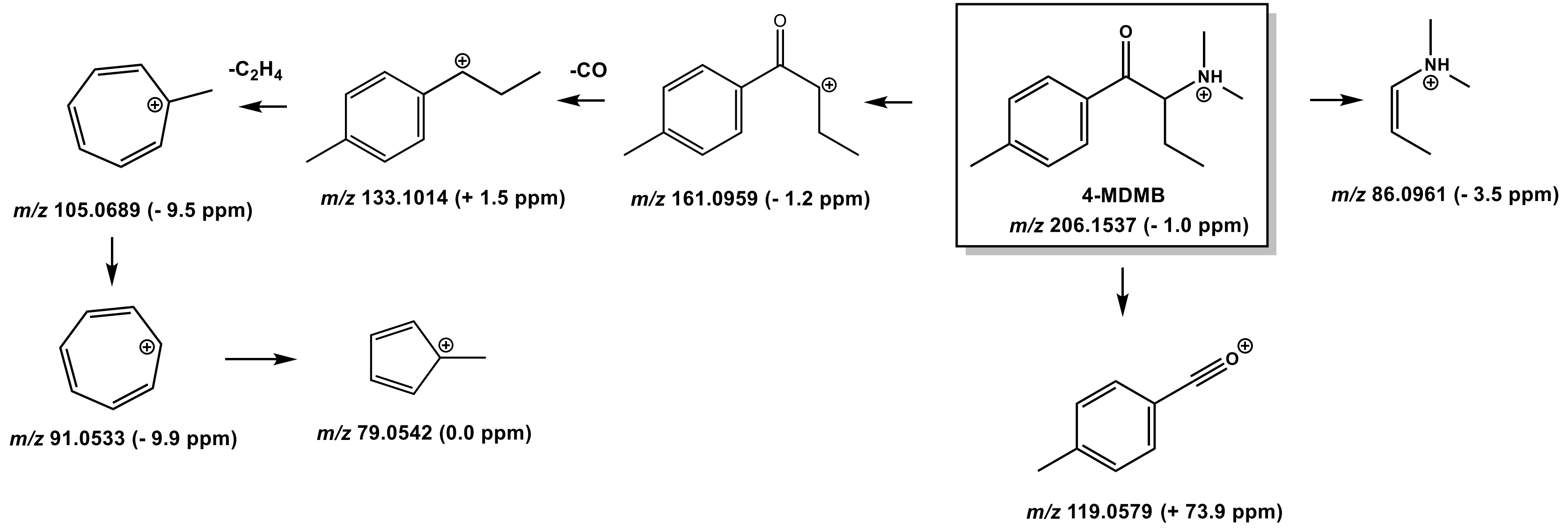

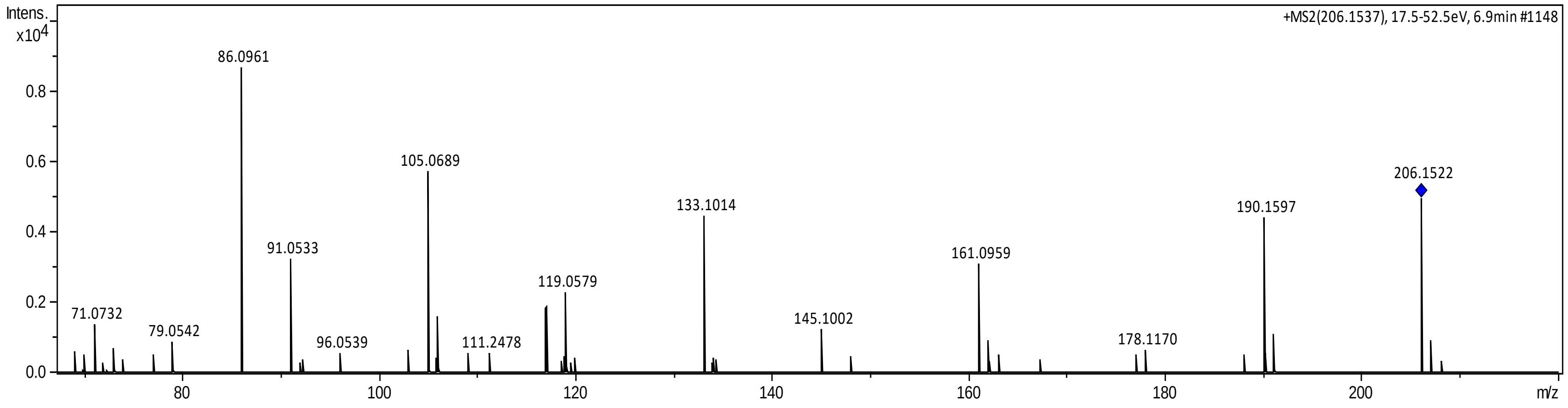

Figure S49: Tandem mass spectrum obtained for **4-MDMB** and proposed structures for the main fragment ions observed.

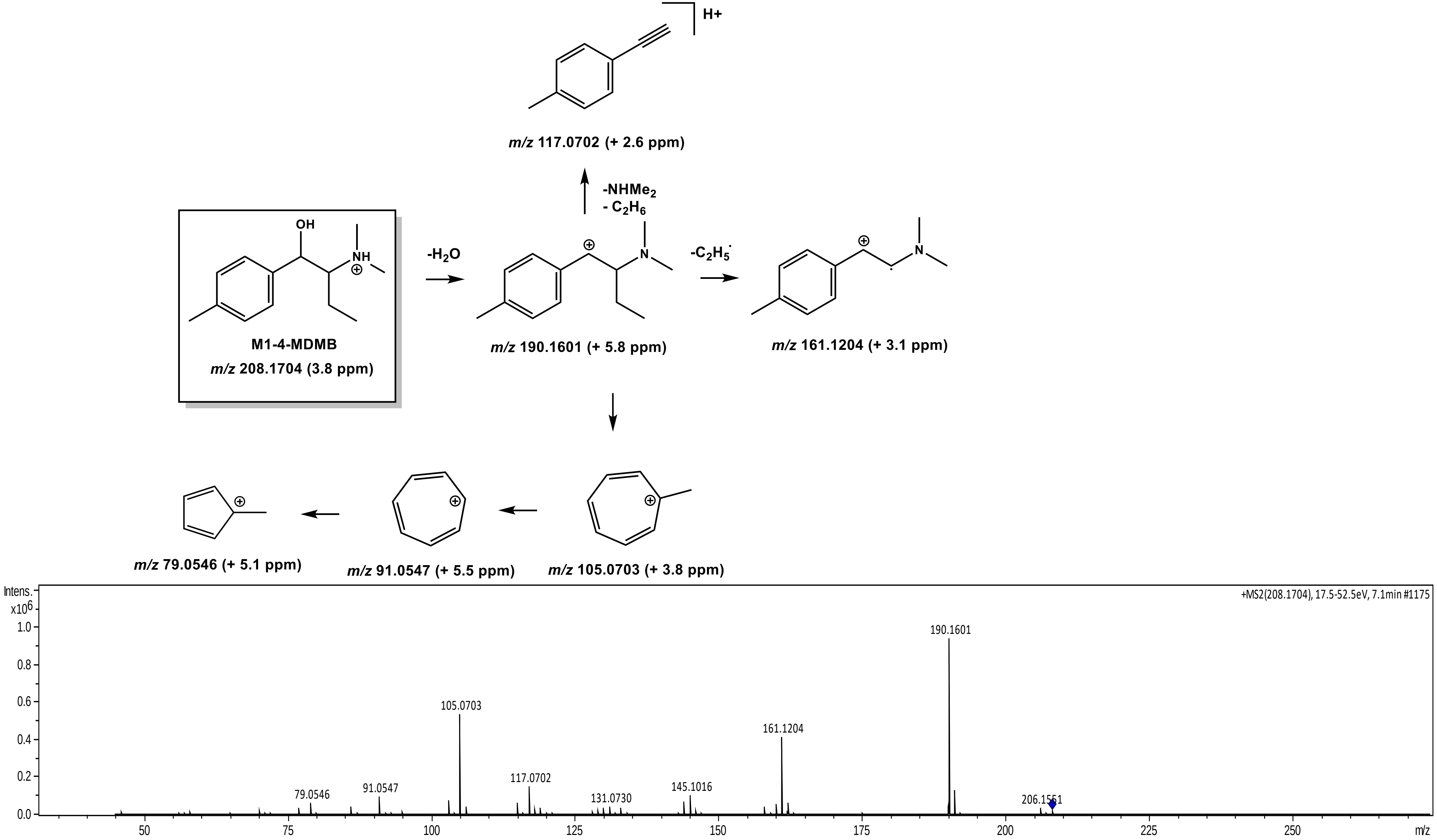

Figure S50: Tandem mass spectrum obtained for **M1-4-MDMB** and proposed structures for the main fragment ions observed.

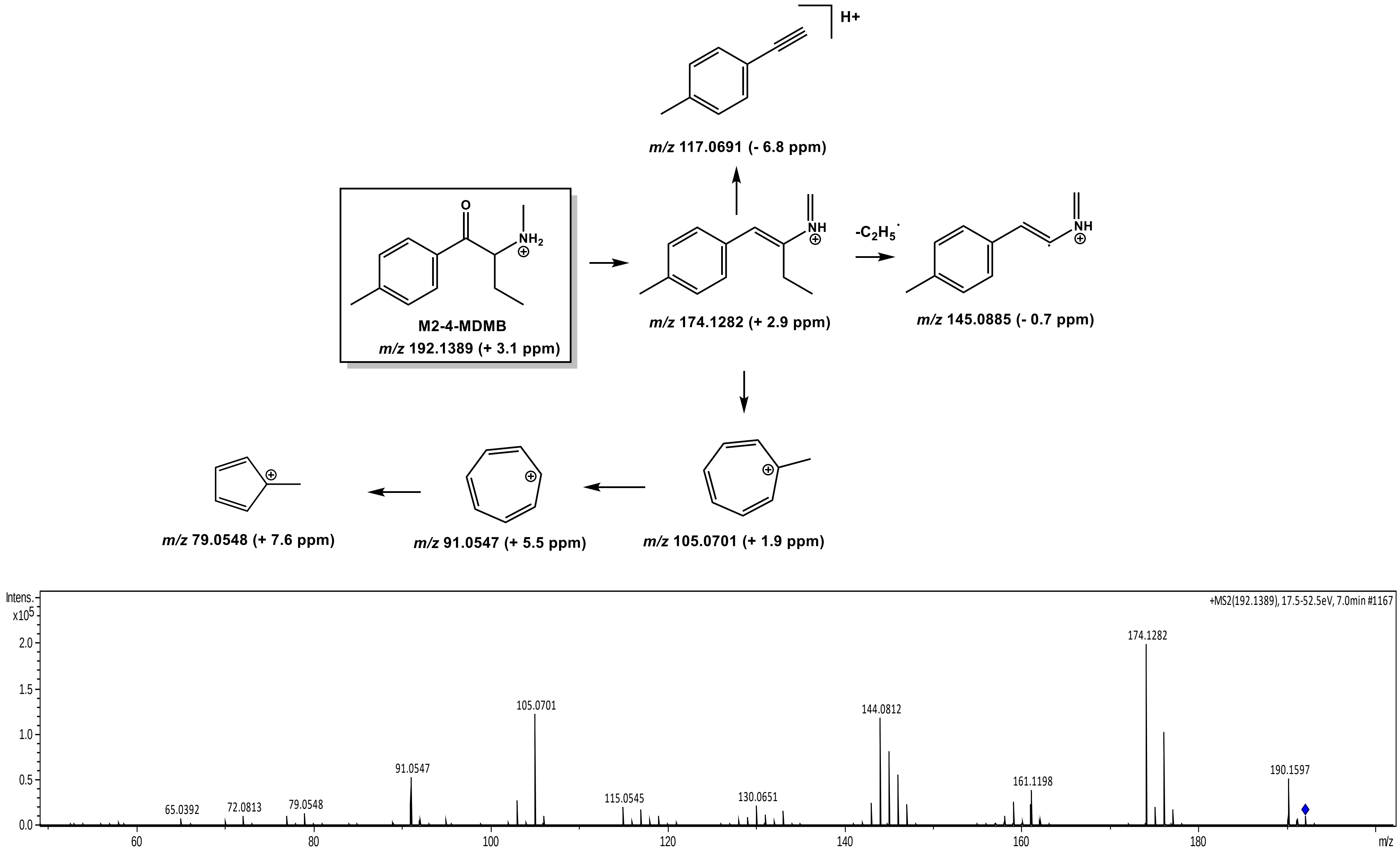

Figure S51: Tandem mass spectrum obtained for **M2-4-MDMB** and proposed structures for the main fragment ions observed.

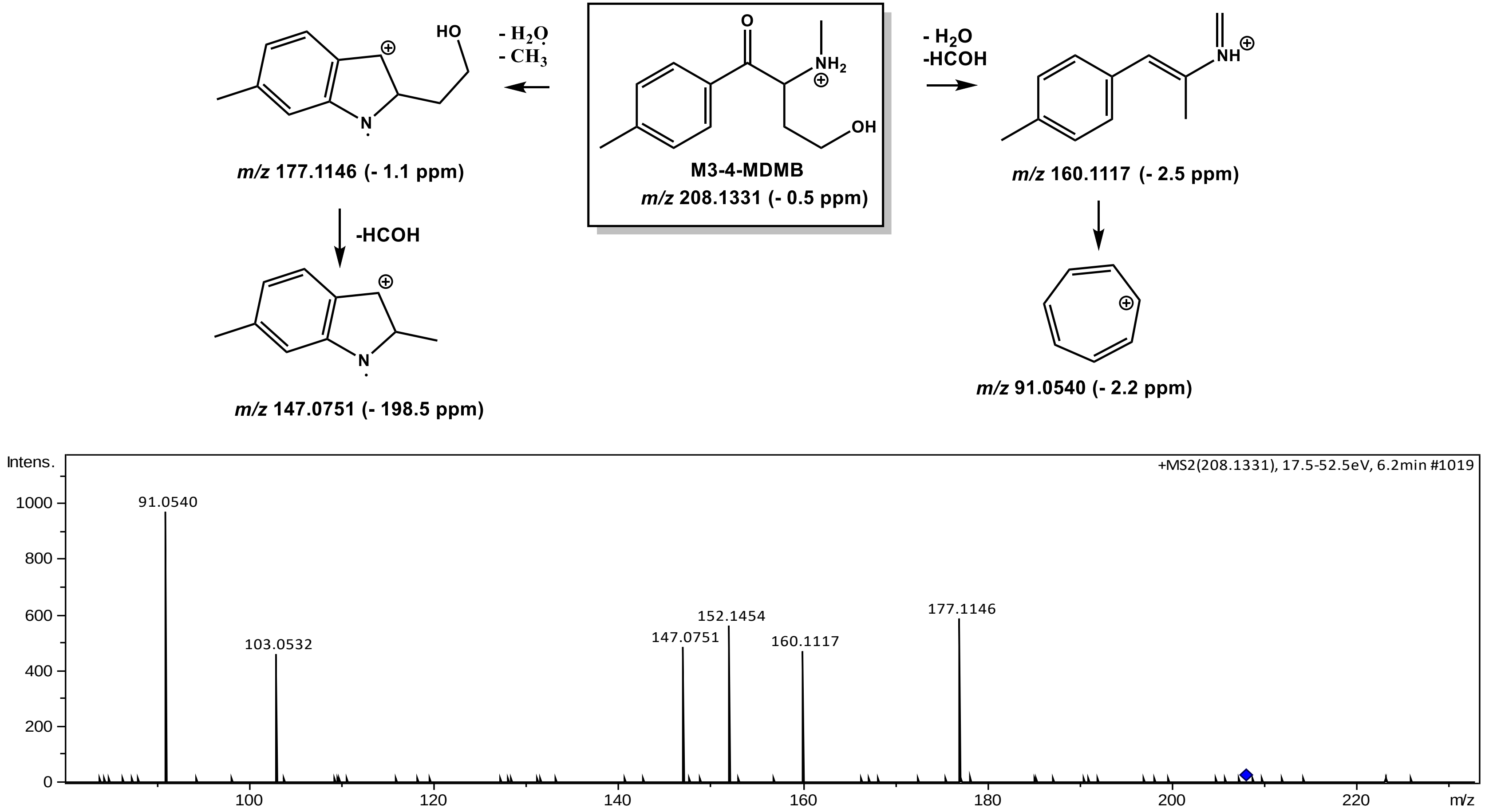

Figure S52: Tandem mass spectrum obtained for **M3-4-MDMB** and proposed structures for the main fragment ions observed.

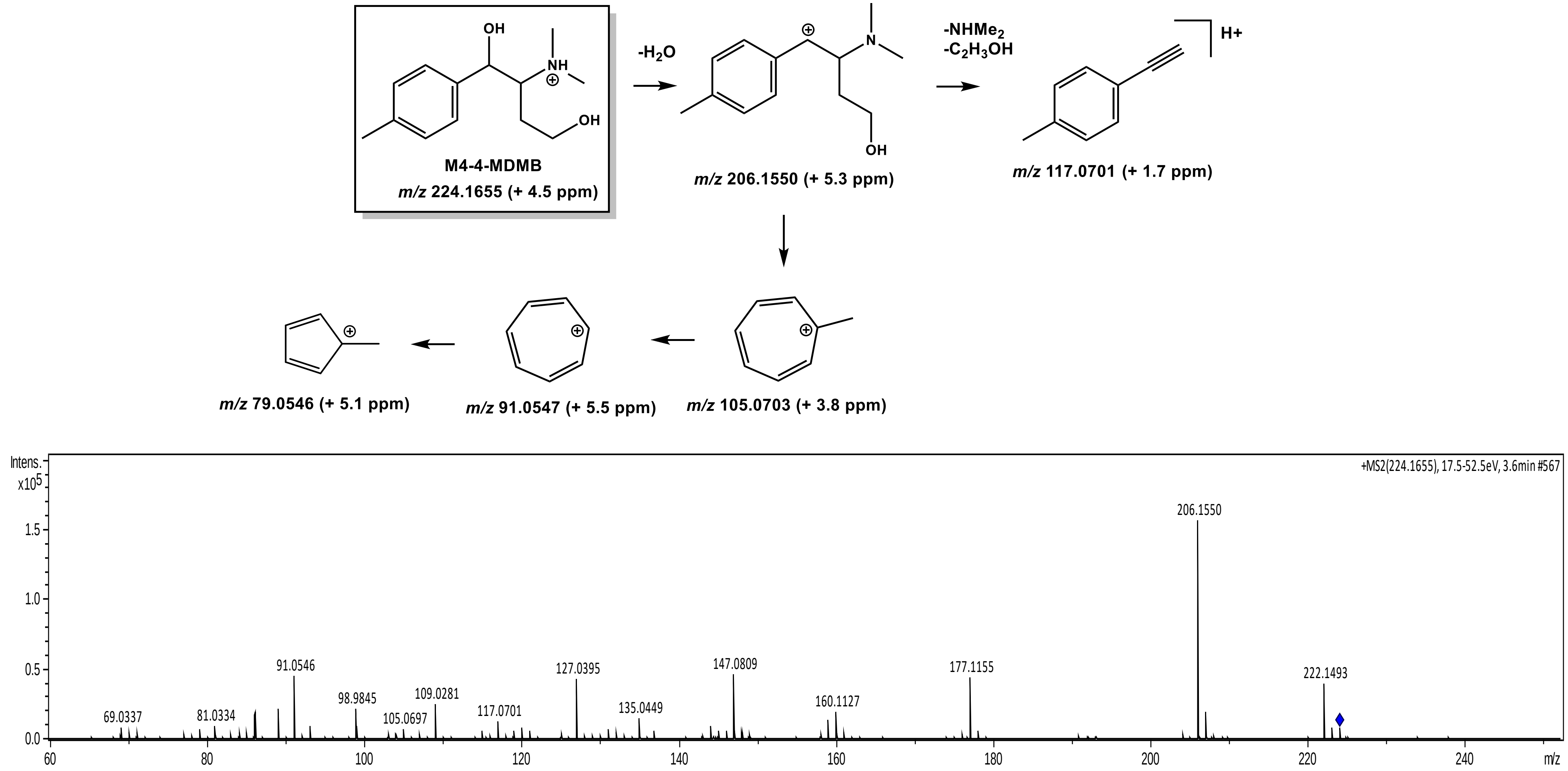

Figure S53: Tandem mass spectrum obtained for **M4-4-MDMB** and proposed structures for the main fragment ions observed.

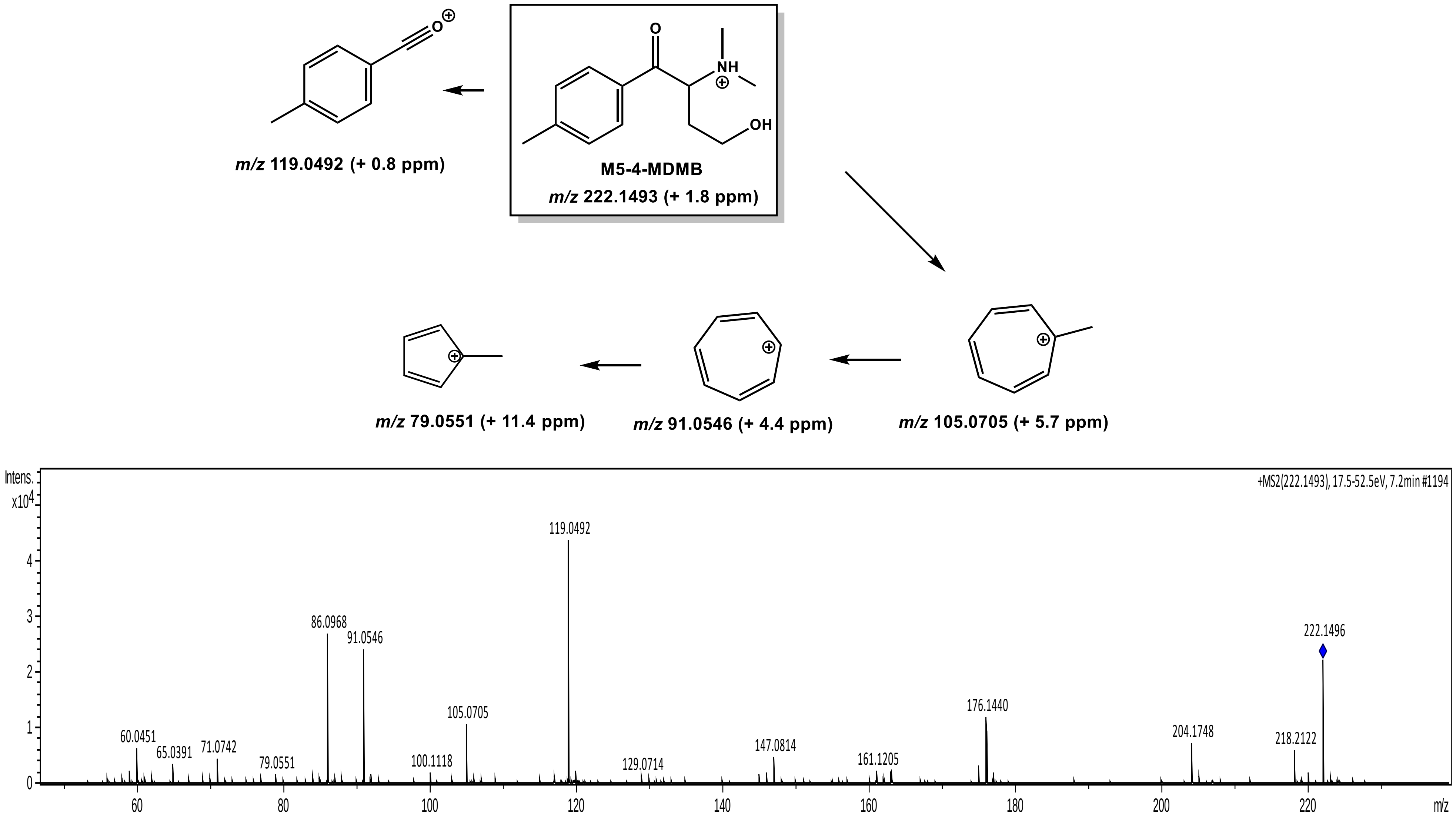

Figure S54: Tandem mass spectrum obtained for **M5-4-MDMB** and proposed structures for the main fragment ions observed.

Table S1: NMR spectroscopic data of chloro-cathinones obtained in DMSO-d6.

	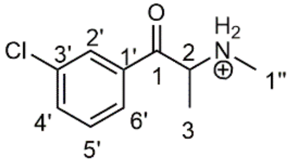		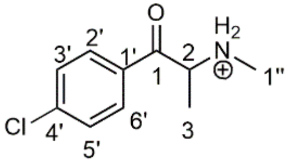		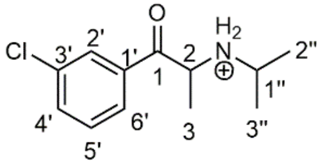		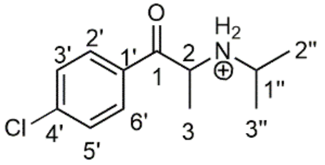		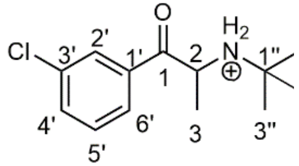	
	**3-CMC**		**4-CMC**		**3-ClC**		**4-ClC**		**Buproprion**	
**Position**	^{13}C NMR*	^{1}H NMR**	^{13}C NMR*	^{1}H NMR**	^{13}C NMR*	^{1}H NMR**	^{13}C NMR*	^{1}H NMR**	^{13}C NMR*	^{1}H NMR**
**1**	195.6	-	195.5	-	195.3	-	195.3	-	195.5	-
**2**	58.3	5.21 (1H; m)	58.3	5.17 (1H; m)	54.6	5.27 (1H; m)	54.6	5.25 (1H; m)	53.0	5.32 (1H; m)
**3**	15.2	1.44 (3H; d; 7.0)	15.3	1.44 (3H; d; 7.0)	15.8	1.47 (3H; d; 7.1)	15.9	1.47 (3H; d; 7.1)	18.1	1.52 (3H; d; 7.0)
**1’**	134.9	-	131.7	-	134.2	-	131.5	-	134.1	-
**2’**	128.4	8.07 (1H; s)	130.8	8.06 (1H; d; 8.2)***	128.5	8.20 (1H; brs)	131.1	8.17 (1H; d; 8.6)***	128.7	8.27 (1H; brs)
**3’**	134.2	-	129.4	7.69 (1H; d; 8.2)***	134.7	-	129.4	7.69 (1H; d; 8.6)***	134.3	-
**4’**	134.4	7.82 (1H; d; 7.6)	139.7	-	134.4	7.83 (1H; d; 8.0)	139.8	-	134.8	7.86 (1H; d; 8.0)
**5’**	131.3	7.64 (1H; d; 7.9)	129.4	7.69 (1H; d; 8.2)***	131.2	7.65 (1H; d; 7.9)	129.4	7.69 (1H; d; 8.6)***	131.3	7.66 (1H; d; 7.9)
**6’**	127.5	8.00 (1H; d; 8.0)	130.8	8.06 (1H; d; 8.2)***	127.6	8.10 (1H; d; 8.0)	131.0	8.17 (1H; d; 8.6)***	127.8	8.17 (1H; d; 7.8)
**1’’**	30.6	2.59 (3H; s)	30.7	2.58 (3H; s)	48.1	3.35 (1H; m)	48.1	3.36 (1H; m)	58.17	-
**2’’**	-	-	-	-	18.9	1.31 (3H; d; 6.5)***	18.9	1.30 (3H; d; 6.4)***	25.99	1.31 (3H; brs)***
**3’’**	-	-	-	-	18.95	1.31 (3H; d; 6.5)***	19.0	1.30 (3H; d; 6.4)***	25.99	1.31 (3H; brs)***
**4’’**	-	-	-	-	-	-	-	-	25.99	1.31 (3H; brs)***
**NH**	-	9.51 (2H; brs)	-	9.45 (2H; brs)	-	9.04 (1H; brs)	-	9.03 (1H; brs)	-	8.63 (1H; brs)
						9.43 (1H; brs)	-	9.45 (1H; brs)	-	9.60 (1H; brs)

*^{13}C NMR signals are reported in δ (ppm); ** ^{1}H NMR signals are reported by the following order: δ (ppm) (integration; multiplicity; *J* (Hz).; *** overlapping signals.

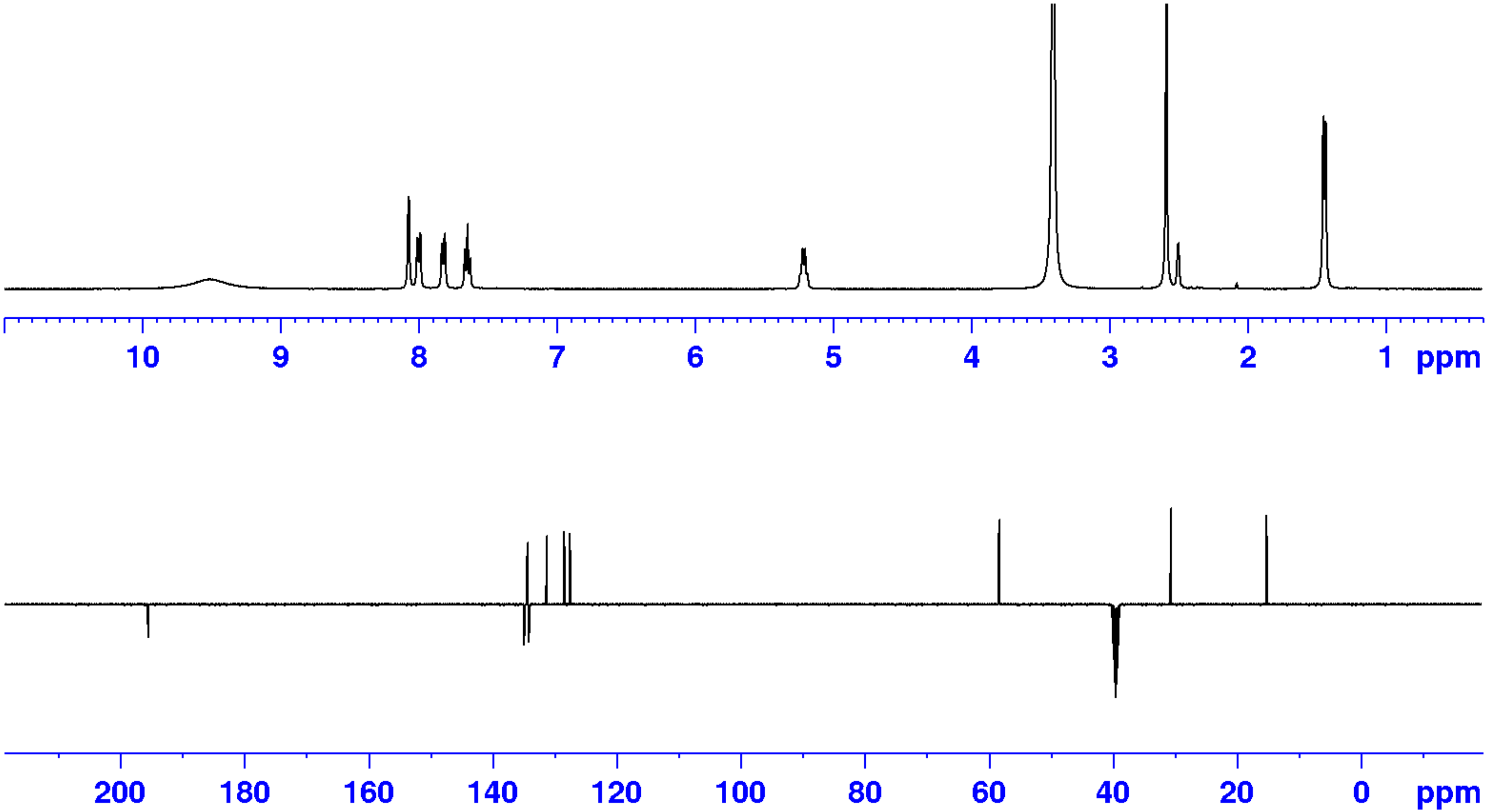

Figure S55: 3-CMC ^{1}H NMR and ^{13}C NMR spectra, recorded in DMSO-d6.

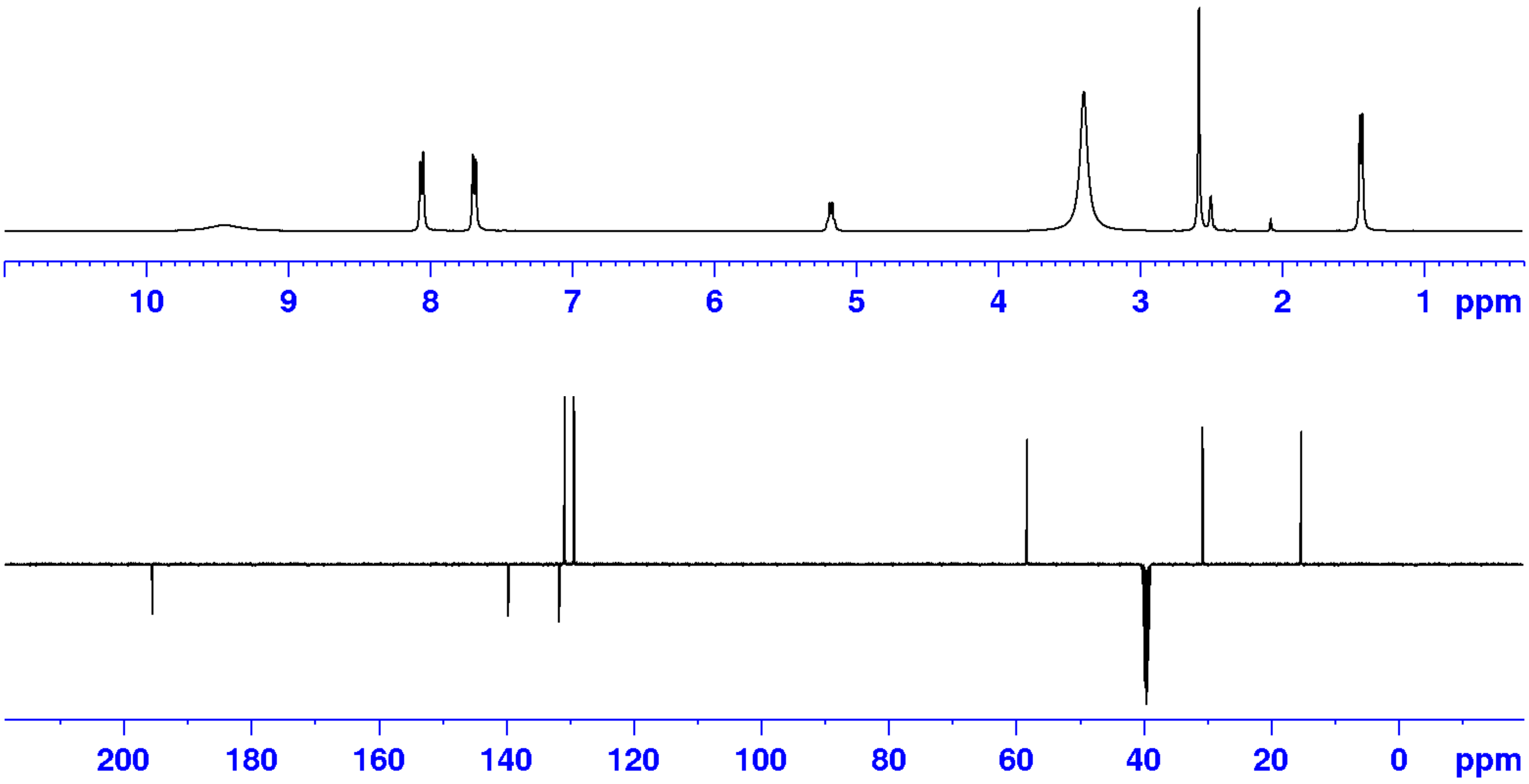

Figure S56: 4-CMC ^{1}H NMR and ^{13}C NMR spectra, recorded in DMSO-d6.

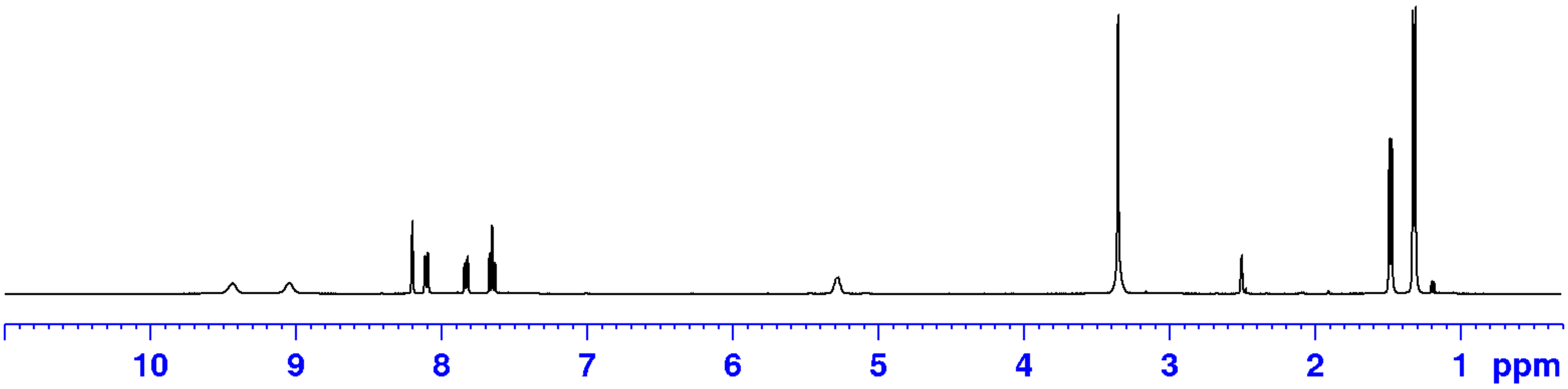

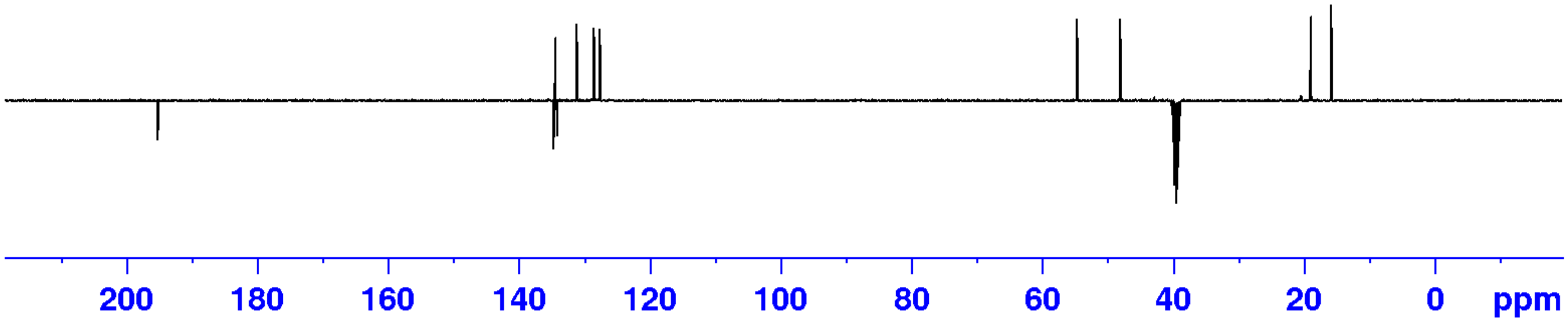

Figure S57: 3-ClC ^{1}H NMR and ^{13}C NMR spectra, recorded in DMSO-d6.

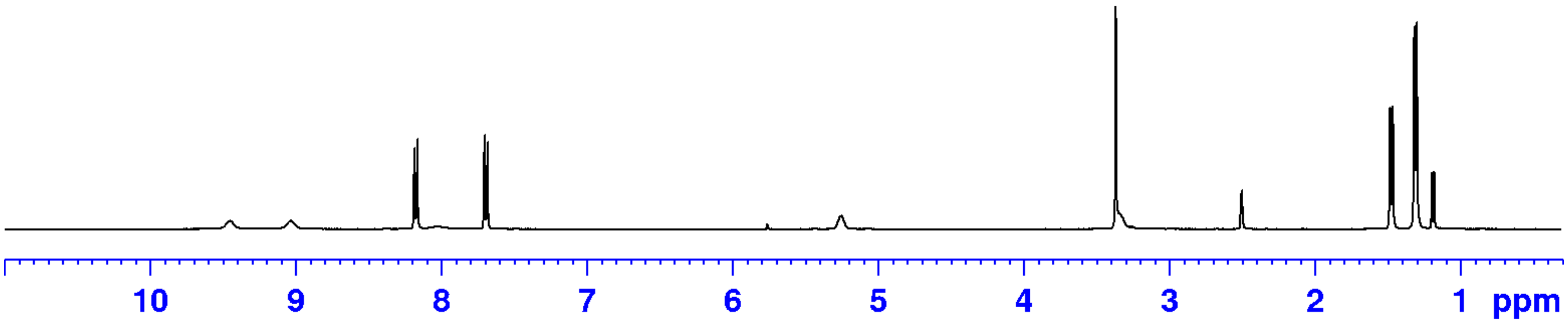

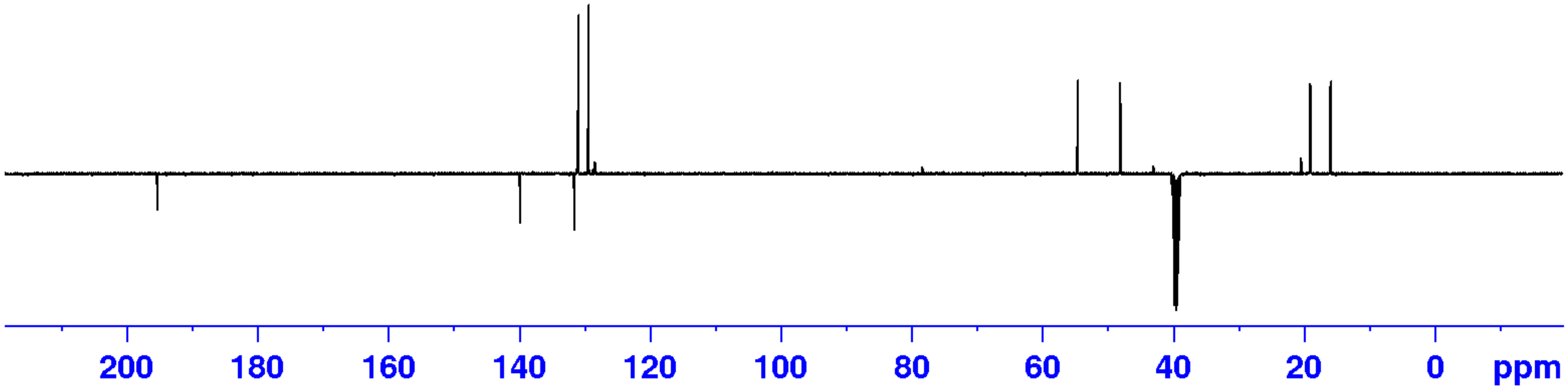

Figure S58: 4-ClC ^{1}H NMR and ^{13}C NMR spectra, recorded in DMSO-d6.

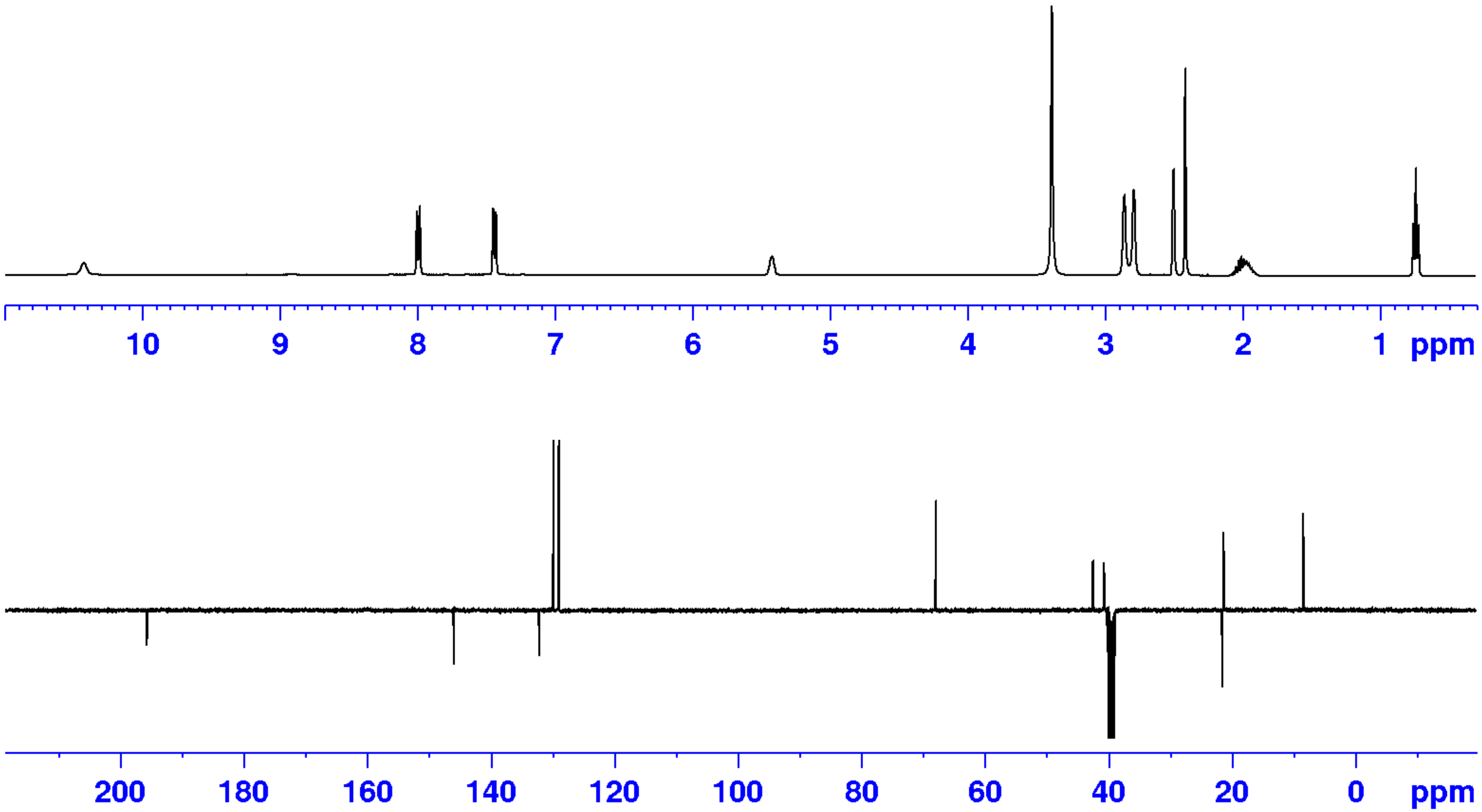

Figure S59: 4-MDMB ^{1}H NMR and ^{13}C NMR spectra, recorded in DMSO-d6.

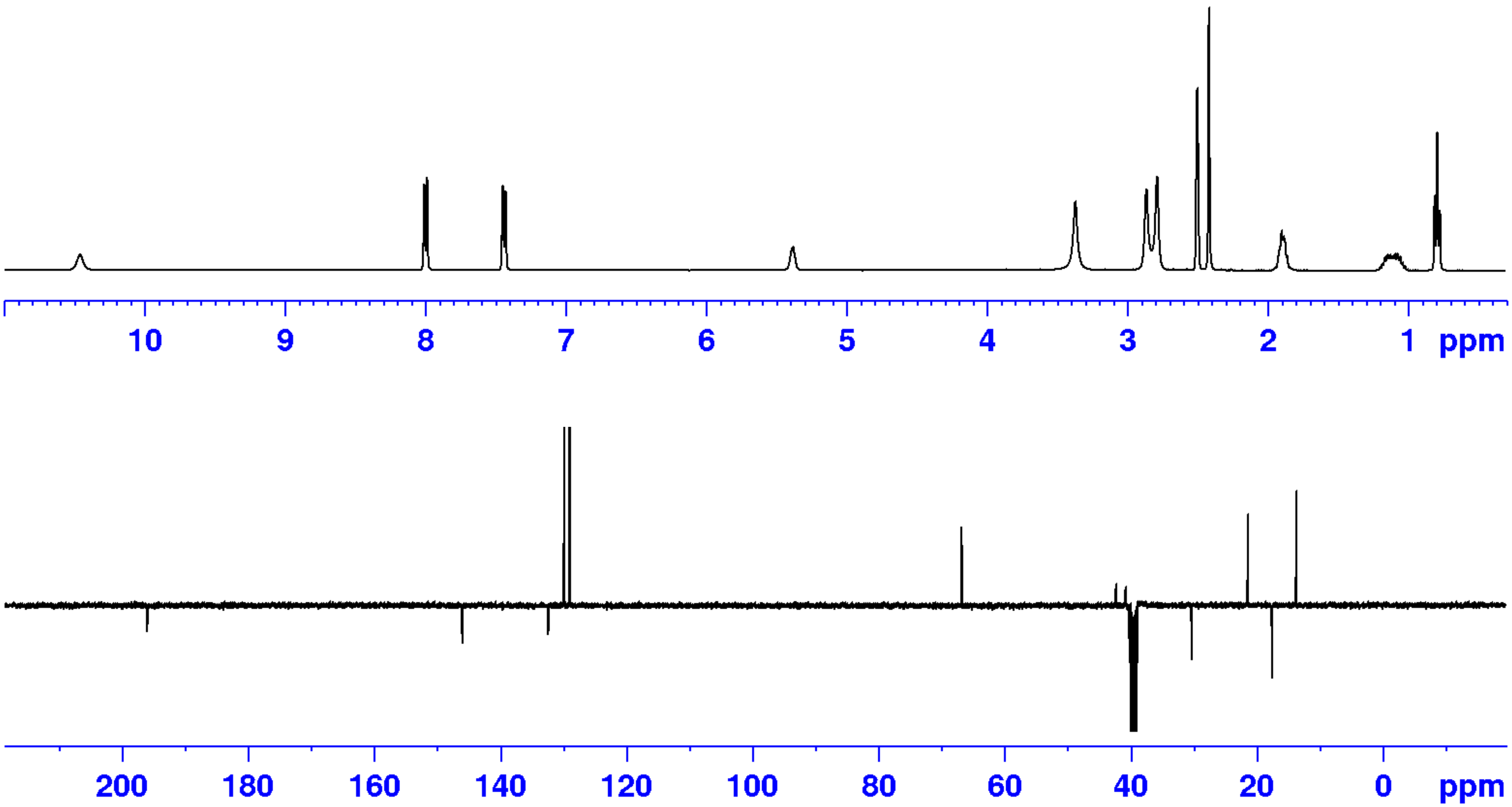

Figure S60: 4-MDMP ^{1}H NMR and ^{13}C NMR spectra, recorded in DMSO-d6.

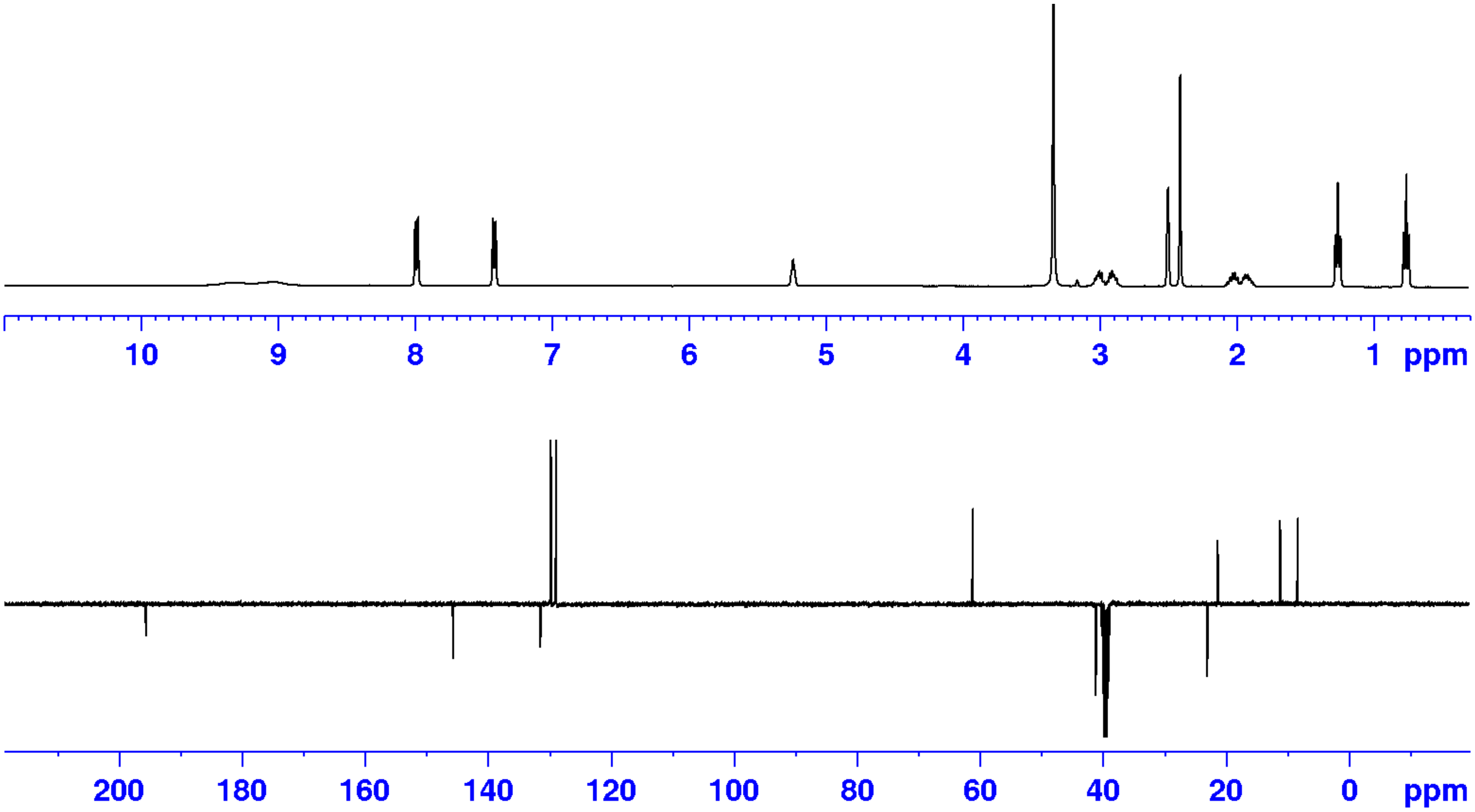

Figure S61: 4-MNEB ^{1}H NMR and ^{13}C NMR spectra, recorded in DMSO-d6.

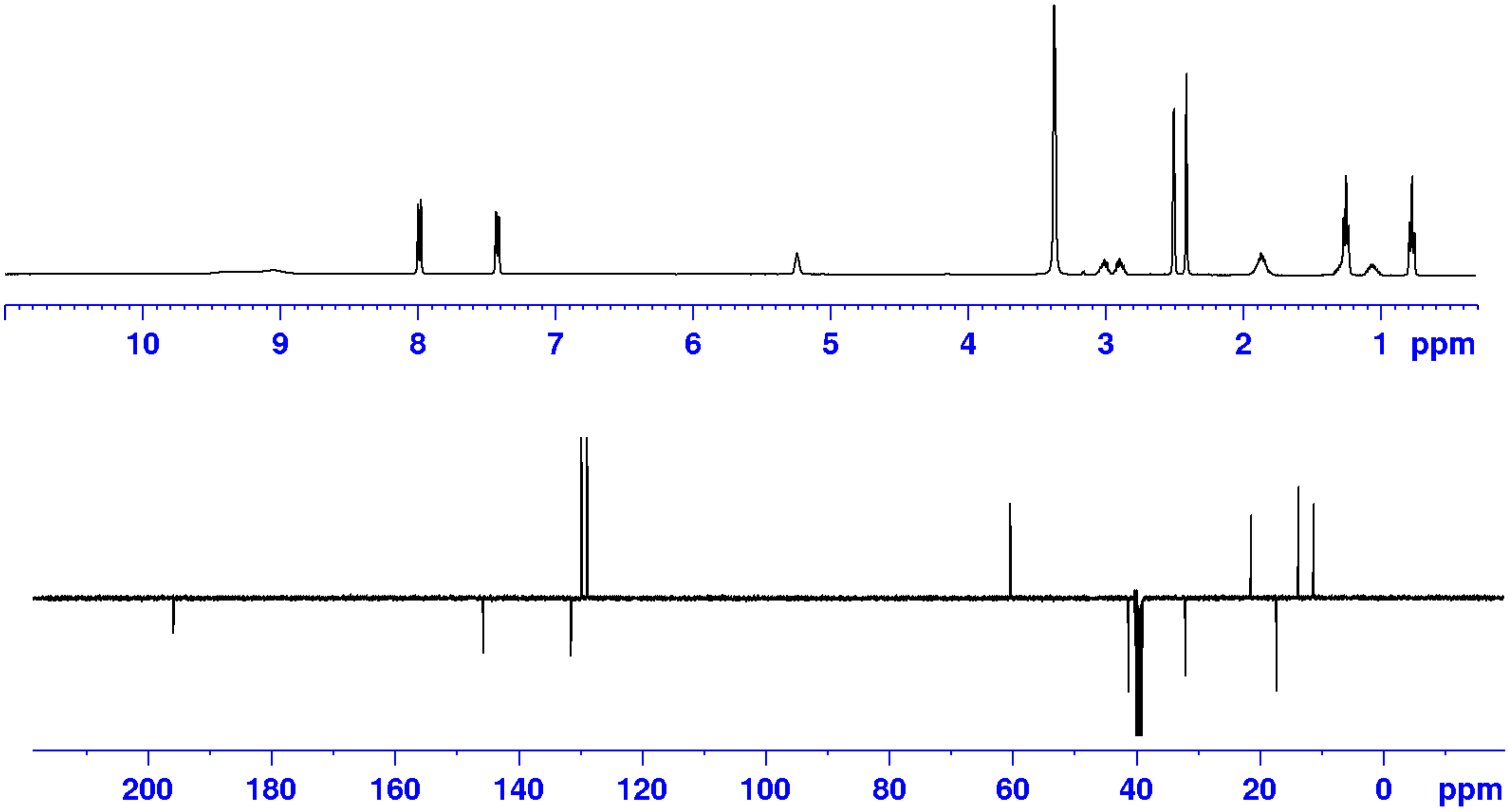

Figure S62: 4-MEAP ^{1}H NMR and ^{13}C NMR spectra, recorded in DMSO-d6.
